# Structural genomics approach to investigate deleterious impact of nsSNPs in conserved telomere maintenance component 1

**DOI:** 10.1038/s41598-021-89450-7

**Published:** 2021-05-13

**Authors:** Arunabh Choudhury, Taj Mohammad, Nikhil Samarth, Afzal Hussain, Md. Tabish Rehman, Asimul Islam, Mohamed F. Alajmi, Shailza Singh, Md. Imtaiyaz Hassan

**Affiliations:** 1 Department of Computer Science, Jamia Millia Islamia, Jamia Nagar, New Delhi, 110025 India; 2 Centre for Interdisciplinary Research in Basic Sciences, Jamia Millia Islamia, Jamia Nagar, New Delhi, 110025 India; 3 National Centre for Cell Science, NCCS Complex, Ganeshkhind, SP Pune University, Campus, Pune, 411007 India; 4 Department of Pharmacognosy, College of Pharmacy, King Saud University, Riyadh, 11451 Saudi Arabia

**Keywords:** Computational biology and bioinformatics, Genetics, Structural biology, Diseases

## Abstract

Conserved telomere maintenance component 1 (CTC1) is an important component of the CST (CTC1-STN1-TEN1) complex, involved in maintaining the stability of telomeric DNA. Several non-synonymous single-nucleotide polymorphisms (nsSNPs) in CTC1 have been reported to cause Coats plus syndrome and Dyskeratosis congenital diseases. Here, we have performed sequence and structure analyses of nsSNPs of CTC1 using state-of-the-art computational methods. The structure-based study focuses on the C-terminal OB-fold region of CTC1. There are 11 pathogenic mutations identified, and detailed structural analyses were performed. These mutations cause a significant disruption of noncovalent interactions, which may be a possible reason for CTC1 instability and consequent diseases. To see the impact of such mutations on the protein conformation, all-atom molecular dynamics (MD) simulations of CTC1-wild-type (WT) and two of the selected mutations, R806C and R806L for 200 ns, were carried out. A significant conformational change in the structure of the R806C mutant was observed. This study provides a valuable direction to understand the molecular basis of CTC1 dysfunction in disease progression, including Coats plus syndrome.

## Introduction

**Figure 1:**
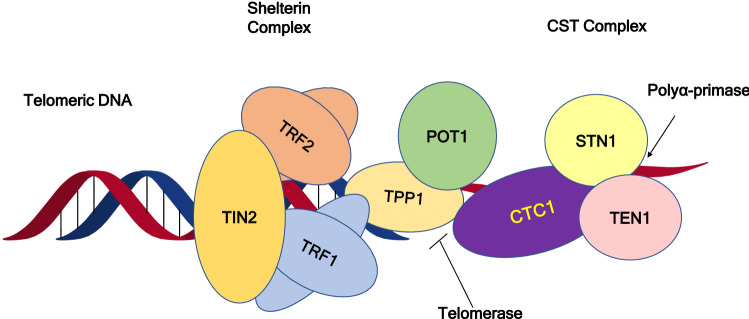
Schematic diagram of the shelterin and CST complexes bound to telomeric DNA. *TRF1* telomeric repeat binding factor 1; *TRF2* telomeric repeat binding factor 2; *TIN2* TRF1 interacting nuclear factor 2; *TPP1* protein encoded by adrenocortical dysplasia homolog (ACD) gene; *POT1* protection of telomerase; *CTC1* conserved telomere maintenance component 1; *STN1* suppressor of CDC thirteen homolog; *TEN1* telomere length regulation protein TEN1 homolog.

Unlike prokaryotic chromosomes, eukaryotic chromosomes are linear and are much larger in size. The ends of the eukaryotic chromosome are composed of a specialized protein-DNA complex called telomeres which maintains the stability of the chromosome ends^1^. The telomeres can be identified as DNA strand breaks by the recombination and repair systems of the cell, which can proceed to end-to-end chromosome fusion and genomic instability leading to apoptosis^2,3^. The mammalian telomeric DNA contains double-stranded 'TTAGGG' repeats followed by 3′ G-rich single-stranded overhangs, and a T-loop structure is formed^4^. The 3′ G overhang forms G-quadruplex, which protects the telomere and inhibits the telomerase-dependent telomere extension^5^. The telomeres and the telomere-binding components shelterin suppress the unwanted DNA damage response and promote the complete replication of the human genome, thus preventing senescence, which is usually associated with significant telomere shortening^6,7^. The two telomere binding components are shelterin and CST (CTC1-STN1-TEN1). Shelterin localizes specifically to double and single-stranded telomeric DNA. It consists of six subunits, including, a telomeric repeat binding factor 1 (TRF1), telomeric repeat binding factor 2 (TRF2), TRF1- interacting nuclear factor 2 (TIN2), adrenocortical dysplasia homolog (ACD, now referred as TPP1), protection of telomerase (POT1), and repressor/activator protein (RAP1)^8,9^. Figure 1 illustrates the components of shelterin and CST complex.

The human CST complex is composed of three proteins, namely conserved telomere maintenance component 1 (CTC1), suppressor of CDC thirteen homolog (STN1) and telomere length regulation protein TEN1 homolog TEN1^10,11^. The CST complex plays a key role in the synthesis of telomeric C-strand^12^. Initially, the CST complex was identified to stimulate the DNA polymerase alpha (Polα) and have an essential role in telomere replication^13^. CTC1-TEN1 component of CST localizes to the single-stranded telomeric DNA and regulates the overextension of G-overhang by regulating the telomerase activity. The TEN1 protein stabilizes the interaction of CTC1-TEN1^14,15^. The CST complex is involved in restarting the replication process after fork stalling during the replication stress^16^. The telomeric G-rich part is very susceptible to G-rich repeats (G-quadruplexes or G4), which creates problems for the telomere replication machinery^17^, and the CST complex helps in removing the G4 regions^18^. The CST complex also binds to the 3′ end of the telomeres and regulates the DNA polymerase-α mediated syntheses of C-strand^19^. The misregulation of CST can affect the telomere length and it can lead to the formation of 3′ overhangs^20^.

**Figure 2:**
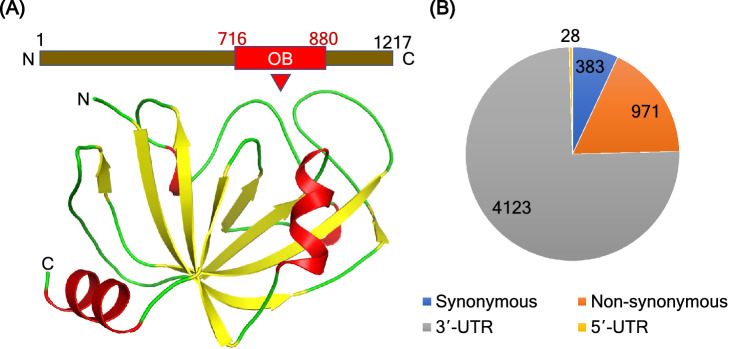
Structural features and mutational distribution in CTC1. (**A**) Structure of the C-terminal OB-fold of CTC1 protein (lower panel) and its position in the protein sequence (upper panel). Figure was drawn using PyMOL (https://pymol.org/2). (**B**) Representation of the number of SNPs in CTC1 using dbSNP database.

CTC1 is composed of 1217 amino acid residues (UniProtKB ID: Q2NKJ3). The homolog of CTC1, Cdc13 is found in yeast *(Saccharomyces cerevisiae*) has four OB folds^21^. OB folds consist of β-barrels, comprising of 5 highly coiled β-sheets^22^. The CTC1 protein contains three OB folds; whereas, the STN1 and TEN1 contain only one OB-fold each^12,23^. The N-terminal OB folds 1 and 2 of CTC1 form a tandem repeat and is required to bind the CST complex to ssDNA, whereas the C-terminal OB-fold forms complex with STN1 and TEN1. Though the human CTC1 protein is a homolog of yeast Cdc13, the current evidence suggests that the human CTC1 does not employ telomerase to the telomeres like Cdc13^24,25^. It is suggested that CTC1 is involved in downregulating the telomerase activity, possibly through its interaction with TPP1, which is also known as a telomerase activity terminator^23,26,27^. A few years back, the structure of the C- terminal OB-fold of the CTC1 protein was characterized (PDB ID: 5W2L) (Fig. 2A). This crystal structure starts from residue 716 to 880, and it revealed classical OB-fold with extended loops^28^. Deletion of this region decreases the STN1-TEN1 complex by 20%. The knockdown of the C-terminal OB-fold increases the length of the G-overhang. This human CTC1 OB-fold consists of three loops from residues (i) 745–759, (ii) 777–795 and (iii) 824–849^22,29–31^. Recently, a full-length structure of the CTC1 complex with STN1-TEN1 was also determined using cryoEM with a resolution of 3.0 Å (PDB ID: 6W6W). The structure consists of seven OB-folds (OB-A, OB-B, OB-C, OB-D, OB-E, OB-F and OB-G)^12^.

Several naturally occurring mutations of the CTC1 protein cause Coats plus (CP) syndrome and Dyskeratosis congenita (DC) or bone marrow syndrome^27,32,33^. CP syndrome is an inherited condition characterized by an eye disorder called Coats disease plus abnormalities of the brain, bones, gastrointestinal system, and other body parts. DC is characterized by fingernails and toenails that grow poorly or are abnormally shaped (nail dystrophy); changes in skin coloring (pigmentation), especially on the neck and chest. Almost 20 mutations are found in CTC1, which inhibit the protein from binding to single-stranded DNA or interact with polymerase-α or binding to the STN1-TEN1 complex^34,35^. The shortening of telomere and development of CP syndrome are found to be associated with each other. Mutations in the *CTC1* gene are responsible for CP, and their association with human disease is typically biallelic^36–38^.

All the non-synonymous SNPs (nsSNPs) are not structurally or functionally affecting, but many missense mutations are deleterious to human health^39^. One-third of the non-synonymous mutations are suggested to be deleterious from experimental studies^40^. Experimental studies done by Shastrula et al. suggest that the naturally occurring mutation R840W and V871M are more stabilizing^28^. The increased stability due to R840W is because of the introduction of hydrophobic tryptophan in the side chain. For V871M, it is due to the introduction of hydrophobic methionine, which increases the contact with surrounding residues. Taking the opportunity into consideration and the fact that CTC1 plays a crucial role in telomere maintenance and several non-synonymous mutations cause diseases, we intended to predict the effects of some nsSNPs on CTC1 using state-of-the-art computational methods^41–43^. We have taken the 971 mutations of the whole protein for sequence analysis and 126 mutations that lie in the C-terminal OB-fold of CTC1 for the complete study. The present study will offer an in-depth analysis of 126 nsSNPs and their effect on the structure and function of CTC1 protein.

## Materials and methods

### Retrieval of data

The sequence of human CTC1 protein was retrieved from the UniProt database (UniProt ID: Q2NKJ3) in FASTA format. A list of nsSNPs was prepared from the data available in dbSNP^44^, HGMD^45^, ClinVar^46^ and Ensembl^47^ databases, and literature search through PubMed. The duplicate nsSNPs were removed from the list. The crystal structure of the C-terminal OB-fold domain of human CTC1 was downloaded from the Protein Data Bank^29^ (PDB ID: 5W2L) (Fig. 2A). Four major classes of SNPs in CTC1 protein obtained from dbSNP and Ensembl are shown in Fig. 2B.

### Prediction of deleterious mutations using sequence-based tools

Deleterious or damaging SNPs in CTC1 were predicted through various tools that are available through different public domains. A brief description of the tools and methods used in sequence-based predictions is given below:

#### SIFT

Sorting Intolerant from Tolerant (SIFT) (http://sift.jcvi.org/) algorithm is used to determine whether the non-synonymous amino acid substitutions are deleterious or not based on sequence homology and physical properties of the amino acid. If the SIFT score is less than or equal to 0.05, then the mutation is not tolerable^48^. A total of 971 nsSNPs were retrieved for the human CTC1 protein. The effect of these nsSNPs on the protein was predicted using the SIFT tool.

#### PolyPhen-2

Polymorphism phenotyping-2 (PolyPhen-2) (http://genetics.bwh.harvard.edu/pph2/) is a sequence-based tool that accepts FASTA file format as input^49^. This tool considers the comparative and physical properties and estimates the damaging probability of the amino acid substitutions. It gives the Position-Specific Independent Count (PSIC) score for the mutant and then calculates the score deviation with the wild-type (WT). If the PSIC score is greater than 0.09, then the non-synonymous mutation is predicted as a deleterious mutation.

#### PROVEAN

Protein variation effect analyzer (PROVEAN) (http://provean.jcvi.org/) was used to identify the damaging missense mutations of CTC1 protein. It estimates the consequence of the mutations on the functionality of the protein^50^. PROVEAN score of less than − 2.5 for an nsSNP is considered deleterious, whereas nsSNPs with a score greater than − 2.5 are considered neutral. All the 971 missense mutations of the Human CTC1 protein were analyzed by the PROVEAN tool.

#### Mutation assessor

Mutation Assessor (http://mutationassessor.org/r3/) is a sequence-based tool that predicts the functional impact of an nsSNP on protein. The mutation assessor result is based on multiple sequence alignment and evolutionarily conserved residues^51^. The tool takes UniProt protein accession or NCBI Refseq protein ID as input for protein sequence and subsequently classifies the mutations as medium, low, or neutral for deleterious effects. The mutation assessor gives FI score for every non-synonymous mutation. If the FI score is more significant than 2.00, then the mutation is considered deleterious.

#### PON-P2

PON-P2 (http://structure.bmc.lu.se/PON-P2/) is a machine learning-based classifier for the classification of amino acid substitutions on human proteins^52^. It classifies the amino acid variants into pathogenic, neutral, and unknown categories. It can efficiently analyze large-scale variant datasets in less time. It also uses GO annotations and functional annotations, if available. PON-P2 takes nsSNP data in various formats. It requires amino acid substitution(s) and one of Ensembl gene identifiers or Entrez gene identifiers, UniProtKB/ accession ID, for identifier submission.

### Prediction of the destabilizing nsSNPs using structure-based tools

The tools and methods used in structure-based predictions are described below:

#### STRUM

STRUM (https://zhanglab.ccmb.med.umich.edu/STRUM/) is a tool which calculates the change in ΔΔ*G* between WT and mutant protein^53^. 3D models are generated starting from WT protein by the iterative threading assembly refinement (I-TASSER) simulations and gradient boosting regression method. Physics and knowledge-based energy functions are incorporated on the structures modelled by I-TASSER and used to train STRUM models. One of the unique features of STRUM is that it combines various methods like multiple sequence alignment; some structural profile scores and gives the sequence profile score, which shows the probability of the given amino acid at a mutant position being found in the ensemble of homologous proteins. This tool accepts the FASTA format file as well as the PDB files as an input format. A mutation is destabilizing if the STRUM score, i.e., ΔΔ*G*, is > 0.

#### MAESTROweb

MAESTRO (https://pbwww.che.sbg.ac.at/maestro/web) is a multi-agent stability prediction tool that estimates the free energy change on protein unfolding. It calculates the impact of a point mutation on the stability of the protein by calculating the free energy change (Δ*G*) between the WT and the mutant protein. MAESTRO takes PDB coordinate files as an input and applies machine learning techniques to calculate the Gibbs free energy change. The quality of the prediction decreases when modelled structures are used as input files. If the score for a mutation is less than 0, then the mutation alters the stability of the protein^54^.

#### SDM2

Site-Directed Mutator (SDM2) (http://marid.bioc.cam.ac.uk/sdm2) calculates the change in protein stability between the WT and the mutant protein^55^. It takes the PDB coordinate file as input and uses environment-specific amino acid substitution tables to estimate the point mutation's protein stability. The updated version of environment-specific amino acid substitution tables is based on new parameters like packing density and residue length. The tool was tested with 2690 amino acid substitutions from 132 different 3D structures of proteins. If the ΔΔ*G* is > 0 for a given non-synonymous SNP, SDM2 predicts it as a destabilizing nsSNP.

#### mCSM

mCSM is a novel tool to evaluate non-synonymous mutations that uses a graph-based approach to predict destabilizing mutations. The predictive models are trained with the environment derived from the atomic distance patterns of different residues. According to mCSM, the mutational impact of each amino acid residue is linked with atomic distance patterns surrounding that residue. These distant patterns describe the mutated residue's nature in the WT protein. This tool gives a better understanding of the mutations associated with diseases for a range of proteins. A mutation has an altering effect on a protein structure if the mCSM score (ΔΔ*G*) is less than 0^56^.

#### DUET

DUET (http://biosig.unimelb.edu.au/duet/) is an integrated tool to study the effect of nsSNPs on protein stability. It uses Support Vector Machine and integrates the scores of mCSM and SDM and gives the combined value of ΔΔ*G*. DUET combines secondary structure and pharmacophore vector (used by mCSM) and residue relative solvent accessibility (used by SDM) and integrates it with supervised learning. The accuracy of the combined method is verified with the experimental thermodynamic data present in the training dataset. The input for DUET is the PDB structure file along with single point mutation, and this tool gives DUET score as well as mCSM and SDM score in the result^57^.

### Identification of Pathogenic nsSNPs

#### PMut

PMut (http://mmb.irbbarcelona.org/PMut) is used to predict the nsSNPs which are associated with the disease phenotype. The manually curated datasets obtained from Swiss-Prot is used to train the neural network-based classifier of PMut and predicted physicochemical properties and sequence conservation are used as prominent features. The updated version has enabled people to generate their predictors for specific families of proteins. It also gives access to the repository of the pre-estimated predictions. PMut score for a pathogenic single point mutation is more significant than 0.05^58^.

#### MutPred2

MutPred2 (http://mutpred.mutdb.org) is a web-based tool that classifies an amino acid substitution as disease-causing or neutral. MutPred2 uses a machine learning-based approach to predict the pathogenicity of a mutation and gives the molecular mechanism of pathogenicity. It also predicts the impact of a mutation in 50 different protein properties. If the MutPred2 score is greater than 0.5 then the mutation is pathogenic^59^.

### Analysis of packing density and accessible surface area

Apart from predicting deleterious mutations, the SDM2 tool also calculates the relative side-chain solvent accessibility (RSA), residue depth and residue-occluded packing density (OSP) for the WT and mutant proteins. The newly updated version of SDM2 includes these features mentioned above. It uses environment-specific amino acid substitution tables to estimate residue depth, RSA and OSP for the protein variants. Lee and Richards's method has been used to calculate RSA. For the analysis of structural stability, residue depth and OSP are also very prominent properties of the protein structure^60^.

### Analysis of aggregation propensity

Solubility based on Disorder and Aggregation (SODA) (http://protein.bio.unipd.it/soda/) is a tool to calculate the aggregation, disorder, helix and strand propensity which arise due to the mutations. This tool takes protein sequence or the PDB format structure fila as an input. SODA predicts different variations like insertion, deletion, substitution and duplication using the PASTA 2.0, ESpritz-NMR and Fells methods. SODA gives a final score based on the difference of solubility between the WT and mutant protein^61^.

### Analysis of noncovalent interactions

The Arpeggio server calculates the number of interatomic interactions of a protein structure^62^. About 15 types of interatomic interactions can be calculated by Arpeggio. PDB format structure files can be submitted to this server for interaction analysis. Arpeggio assigns atom types to each atom using OpenBabel via SMARTS queries. It finds the nearest-neighbor atoms within a 5Å radial cutoff and a structural interaction fingerprint (SIFt) is given to each pairwise interatomic contact. It gives the number of interactions and provides downloadable tabular data showing different covalent and noncovalent interactions. The interactions can also be visualized through this tool.

### Analysis of conserved residues

**Figure 3:**
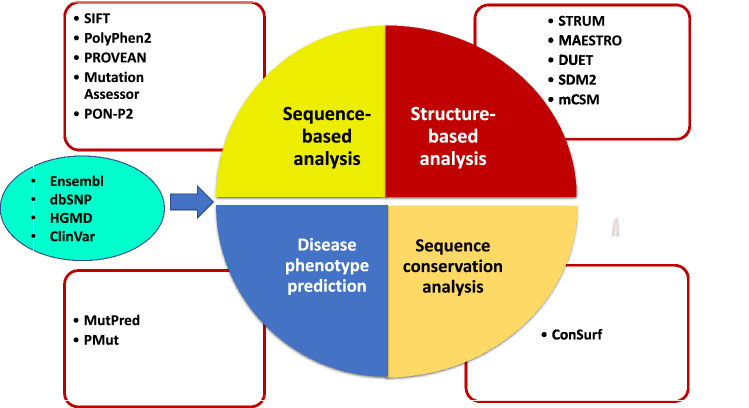
Overview of the used methods to predict the pathogenic mutations of the CTC1 protein at the sequence, structue and function levels.s

ConSurf tool (https://consurf.tau.ac.il/) was used to determine the degree of conservation of residues in a specific position using multiple sequence alignment^63^. The amino acid conservation is vital to understand evolution and the function and structure of a protein. The ConSurf tool uses the empirical Bayesian method or maximum likelihood (ML) to calculate the degree of conservation of each residue. The ConSurf score ranges between 1 and 9, where 1 is the score for most minor conserved positions, 5 is the score for intermediate conserved positions, and nine is for highly conserved positions. The ConSurf-DB also stores the pre-estimated scores for known PDB structures. The buried residues with a high degree of conservation are considered structural residues and the exposed residues with a high degree of conservation are considered functional residues. The computational approach and tools used in the mutational analysis are represented in Fig. 3.

### MD simulations

All-atom MD simulation for 200 ns was carried out on CTC1-WT, R806C, and R806L. MD simulation was performed under explicit solvent conditions at 300 K using the GROMACS 5.1.5 package while utilizing the GROMOS96 43a1 force field as described earlier^64–66^. The solvation was carried out in a cubic box filled with water with a dimension of 10 Å. Appropriate numbers of Na^+^ and Cl^−^ ions were added to all three systems for neutralization while utilizing the *genion* tool in Gromacs. Energy minimization was carried out using 1500 steps of the steepest descent method to remove any steric clashes in the systems. Equilibration was carried out at 300 K using the two-step ensemble process (NVT and NPT) for 100 ps. The final MD run on each system was carried for 200 ns and a leap-frog integrator was used for the production of the time-evolution trajectories.

## Result and discussion

A total of 971 reported nsSNPs were extracted from the dbSNP (http://www.ncbi.nlm.nih.gov/snp), the Human Gene Mutation Database (HGMD) (http://www.hgmd.cf.ac.uk), ClinVar (http://www.ncbi.nlm.nih.gov/clinvar/) and Ensembl (http://www.ensembl.org/) databases. Some of the nsSNPs are not present in these databases, including a literature search on PubMed. Out of the 971 nsSNPs, 126 were mapped in the OB-fold region of human CTC1 protein. The C-terminal OB region structure (residue 716–880) present in the PDB database (PDB ID: 5W2L) was selected for the structural analysis. The present study focuses on the sequence-based analysis of all the missense mutations and the structure-based analysis of mutations that are present in the OB region of the selected protein structure.

A multi-tier approach was employed to identify the structural and functional consequences of the non-synonymous mutations on the *CTC1* gene. Sequence-based and structure-based approaches have been employed to obtain the high confidence deleterious nsSNPs. All the nsSNPs were subjected to sequence-based analysis using five web-based tools: SIFT, PolyPhen2, PROVEAN, Mutation assessor, and PON-P2. For the structure-based approach, STRUM, MAESTRO-web, SDM2, mCSM and DUET were used to analyze the 126 nsSNPs in the OB-fold region of CTC1. Disease phenotype identification of the high confidence nsSNPs had been made using MutPred2 and PMut web server. Figure 3 depicts an overview of all the computational approaches used in this study. We have also employed other approaches, namely analysis of packing density and accessible surface area, analysis of aggregation propensity and degree of amino acid conservation of the OB-fold of the CTC1 protein.

### Identification of deleterious nsSNPs using sequence and structure-based approaches

**Figure 4:**
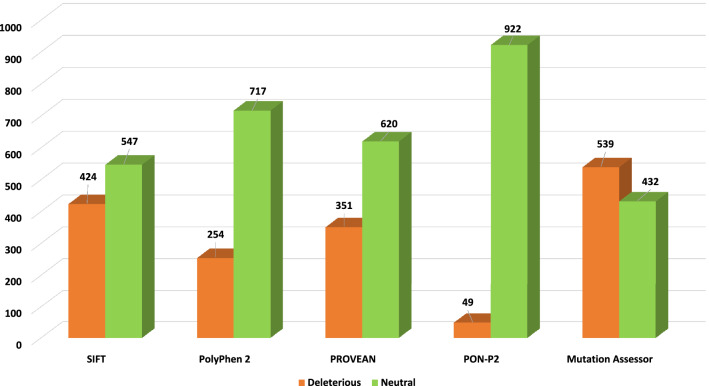
Distribution of deleterious and neutral nsSNPs predicted by sequence-based tools for the entire sequence of CTC1 protein. The vertical axis shows the number of mutations. The horizontal axis shows the sequence-based tools; the orange bar depicts the number of predicted deleterious mutations, and the green bar depicts the number of predicted non-deleterious mutations.

Multiple tools were used to predict deleterious mutations. Because using a single tool can provide some false positives. The use of more tools can eliminate false predictions, and it may provide more accurate results. For the sequence-based approach, SIFT, PolyPhen2, PROVEAN, Mutation assessor and PON-P2 were used (Fig. 4). The SIFT web tool considers the physical properties and classifies the nsSNPs into tolerated (non-damaging) and intolerant (damaging) mutations. A higher value of tolerance index implies a low functional impact of a mutation on the protein and vice versa^48^. PolyPhen2 is another tool that also uses the amino acid sequence to determine damaging mutations. PolyPhen2 quantifies the non-synonymous mutations in three categories: possibly damaging (score > 0.2 and < 0.96), probably damaging (score > 0.96), and benign (score < 0.2). To improve the confidence level, three other tools PROVEAN, Mutation assessor and PON-P2 were used. PROVEAN uses a clustering approach where BLAST hits from the query sequence are used to form clusters, of which around 30 are chosen to generate a prediction. A delta alignment score is calculated for each sequence in a cluster, and the score is averaged to generate a final and default PROVEAN score. PROVEAN score less than − 2.5 signifies a deleterious mutation. The mutation assessor uses conservation scores. Conserved regions are determined from multiple sequence alignment of the query sequences. A conservation score is generated for each region, which characterizes the functional impact of the substitution. A score of more than 3.5 is considered deleterious, a score between 2.0 and 3.5 is probably deleterious, and a score between 0.5 and 2.0 is considered normal. PON-P2 classifies the amino acid variants into pathogenic, neutral and unknown categories using evolutionary conservation and physical and biochemical properties of amino acid.

The disease-causing mutation also alters the stability of a protein. A protein is either in a folded or unfolded form. In thermodynamics, the energy difference (Gibbs free energy) between folded and unfolded (*G*_u_) protein can be calculated as Δ*G* = *G*_u_ − *G*_f_. The change of protein stability and free energy landscape is calculated as ΔΔ*G* = *G*_m_ − *G*_w_, where G_m_ is the mutant protein and G_w_ is the WT protein. More negative ΔΔ*G* value implies more stabilizing mutation and a positive ΔΔ*G* value depicts destabilizing mutations^53^.

Our study has used five different structure-based stability predictors: STRUM, MAESTROweb, SDM2, mCSM and DUET. All of the tools use the PDB structure file of the WT protein as an input. Using the atomic coordinates, they determines the stability of the variants by calculating the folding free energy. Most of these tools use a machine learning-based approach combining various functional genomics approaches and estimate impact of mutations on the structure and stability of protein^41,67,68^.

**Figure 5:**
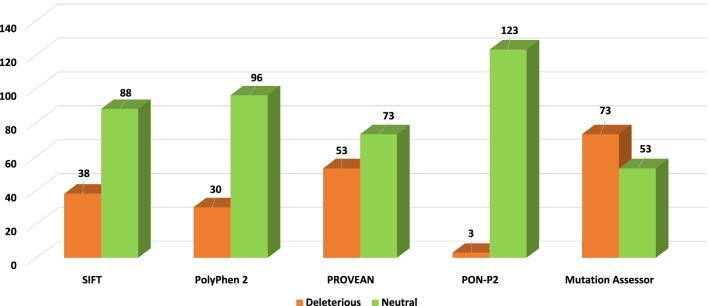
Distribution of deleterious and neutral nsSNPs predicted by sequence-based tools for the C-terminal OB-fold region of CTC1 protein. The vertical axis shows the number of mutations. The horizontal axis shows the sequence-based tools; the orange bar depicts the number of predicted deleterious mutations, and the green bar depicts the number of predicted non-deleterious mutations.

For the sequence-based approach, all the 971 nsSNPs of CTC1 were analyzed. SIFT, PolyPhen2, PROVEAN, PON-P2 and Mutation assessor predicted that out of the 971 missense mutations, 424 (43.66%), 254 (26.16%), 351 (36.15%), 49 (5.04%) and 539 (55.51%) were deleterious, respectively (Supplementary Table S1). Out of the 126 mutations which lie in the C-terminal OB-fold of the protein predicted deleterious mutations by SIFT, PolyPhen2, PROVEAN, PON-P2 and Mutation Assessor were 38 (30.16%), 30 (31.25%), 53 (42.06%), 3 (2.39%) and 73 (57.94%), respectively (Fig. 5).

**Figure 6:**
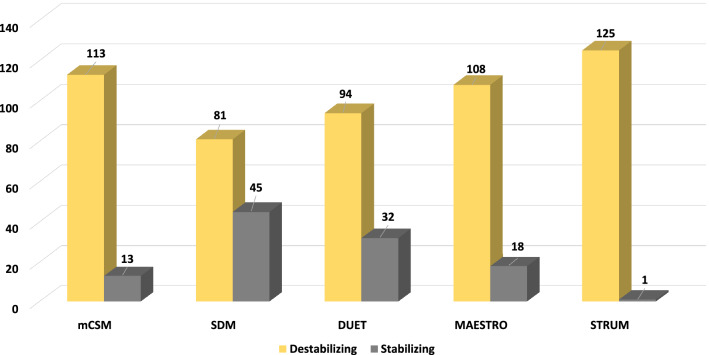
Distribution of destabilizing nsSNPs predicted by structure-based tools for the C-terminal OB-fold region of CTC1 protein. The vertical axis shows the number of mutations. The horizontal axis shows the structure-based tools; the yellow bar depicts the number of predicted destabilizing mutations and the grey bar depicts the number of predicted stabilizing mutations.

This study only focuses on the OB-fold region of human CTC1 protein, further analysis was done only for the missense mutations in this region. Out of the 126 nsSNPs of hCTC1 OB structure-based prediction by STRUM, MAESTROweb, SDM2, mCSM and DUET showed 125 (99.20%), 108 (85.71%), 81 (64.29%), 113 (89.68%) and 94 (74.6%) missense mutations as destabilizing mutations (Supplementary Table S2, Fig. 6). For further study, we have only collected those mutations which are predicted to be deleterious by three different sequence-based tools and four different structure-based tools to increase the confidence level. After filtering out by this approach, 75 (59.52%) mutations were collected are predicted as deleterious and destabilizing by both sequence-based and structure-based approaches. This 75 nsSNPs were then analyzed for disease phenotype association.

### Identification of pathogenic nsSNPs

We have predicted the disease association of non-synonymous mutations using the PMut and MutPred web servers. These two methods find the disease phenotypes and classify the mutations into pathogenic or benign based on the pathogenicity score. Out of a total of 75 high confidence nsSNPs obtained from sequence and structure-based analysis, PMut and MutPred predicted 12 (16%) and 23 (30.67%) nsSNPs as pathogenic, respectively. Out of 75 high confidence nsSNPs, only 11 mutations (S730R, S730G, R731W, R744G, G767R, F800C, R806C, R806L, W807C, R818L, and L860P) were identified as pathogenic from both the disease phenotype prediction tool. The further study focuses on these 11 mutations out of the 75 mutations (Supplementary Table S3–S4). MutPred predicted the pathogenicity of these variations. According to MutPred, the mutations (S730R, S730G, R806C, R806L, W807C, and R818L) showed loss of strand. The mutations S730R, R731W and R744G show gain of helix, strand and loop. The variations F806C, R806C, R806L, W807C, and R818L also alters the ordered interface of CTC1 protein. G767 shows a gain of ADP-ribosylation at that position.

### Analysis of evolutionarily conserved residues

**Figure 7:**
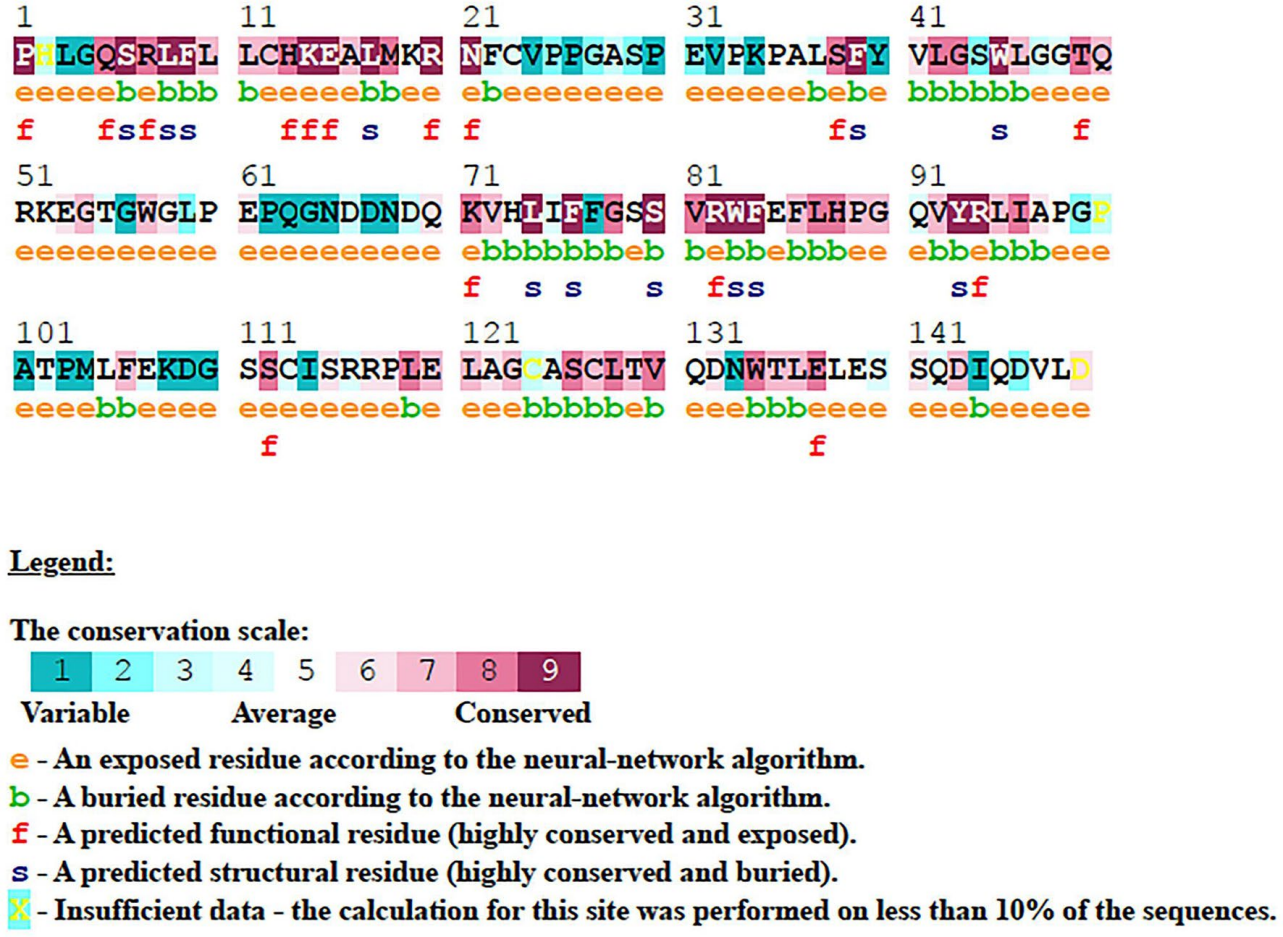
Sequence Conservation analysis of the C-terminal OB-fold region of CTC1 protein using ConSurf webserver.

Analyzing the conservation of amino acid residue in the protein structure can understand the importance of an amino acid residue and discloses its localized evolution^42,69^. The structural integrity of a protein also depends on the conserved residues. The tendency of an amino acid to mutate depends upon the degree of conservation. The OB-fold region of the human CTC1 protein was analyzed with the ConSurf tool for obtaining the degree of conservation of the residues. The ConSurf analysis revealed that amino acid residues between 728 to 745, 792 to 820 and 850 to 861 are highly conserved than the other residues. It was also revealed that most of the highly conserved residues are buried (Fig. 7).

### Analysis of aggregation propensity

The function of a protein is greatly influenced by its solubility. Insoluble parts of a protein try to form aggregates which can cause diseases like Alzheimer's, amyloidosis, and Parkinson's diseases^70,71^. SODA (Solubility based on Disorder and Aggregation) was used to calculate the solubility of the protein variants to find the disease association^61^. SODA calculates the aggregation, disorder, helix, and strand propensity which arise due to the mutations. Out of the 11 mutations obtained from disease phenotype prediction, five nsSNPs decrease the solubility of the protein, whereas the other six mutations increase the solubility of the protein (Table 1).

**Table 1:** Prediction of aggregation propensity of mutant CTC1 protein using SODA server.

Mutations	SODA	Impact
S730R	1.51	More soluble
S730G	2.43	More soluble
R731W	− 10.24	Less soluble
R744G	0.21	More soluble
G767R	− 4.19	Less soluble
F800C	12.64	More soluble
R806C	− 40.10	Less soluble
R806L	− 47.17	Less soluble
W807C	7.58	More soluble
R818L	− 16.54	Less soluble
L860P	6.91	More soluble

### Analysis of noncovalent interactions

Previous reports on the mutational analysis demonstrated that the effect of nsSNPs on the stability of the protein depends upon the changes in hydrophobic contacts. We have calculated the van der Waals, hydrogen bonding, electrostatic, and hydrophobic interactions in WT CTC1 and its mutants with the Arpeggio web server's help. An increase and decrease in the number of bonds show mutations in the local and global environment. The 11 nsSNPs found to be pathogenic were analyzed using the Arpeggio webserver (Table 2).

**Table 2:** Calculation of non-covalent interatomic interactions of wild-type and mutant CTC1 structures using the Arpeggio server.

Variant	van der Waals interaction	Hydrogen bonds	Ionic interactions	Aromatic contacts	Hydrophobic contacts
WT	97	117	18	29	387
S730R	101	115	20	31	399
S730G	98	117	18	31	388
R731W	106	119	16	51	409
R744G	95	116	15	31	382
G767R	105	117	18	33	390
F800C	100	118	18	20	339
R806C	97	117	18	31	386
R806L	98	117	18	31	386
W807C	97	117	18	25	366
R818L	102	114	14	31	393
L860P	102	118	18	31	375

Finally, out of 11 mutations, based on the solubility, aggregation propensity, SODA score and other harmful properties, R806C and R806L along with WT CTC1 were selected for MD simulation study. Both R806C and R806L showed less solubility, high aggregation propensity and were predicted to be deleterious by all of the structure-based tools with the SODA score of − 40.10 and − 47.17, respectively. Protein misfolding and aggregation are involved in many disease progression, including neurodegenerative diseases^72–78^. MutPred2 analysis also showed that both the mutations contribute to loss of strand and alters the ordered interface of CTC1.

### MD simulations

**Figure 8:**
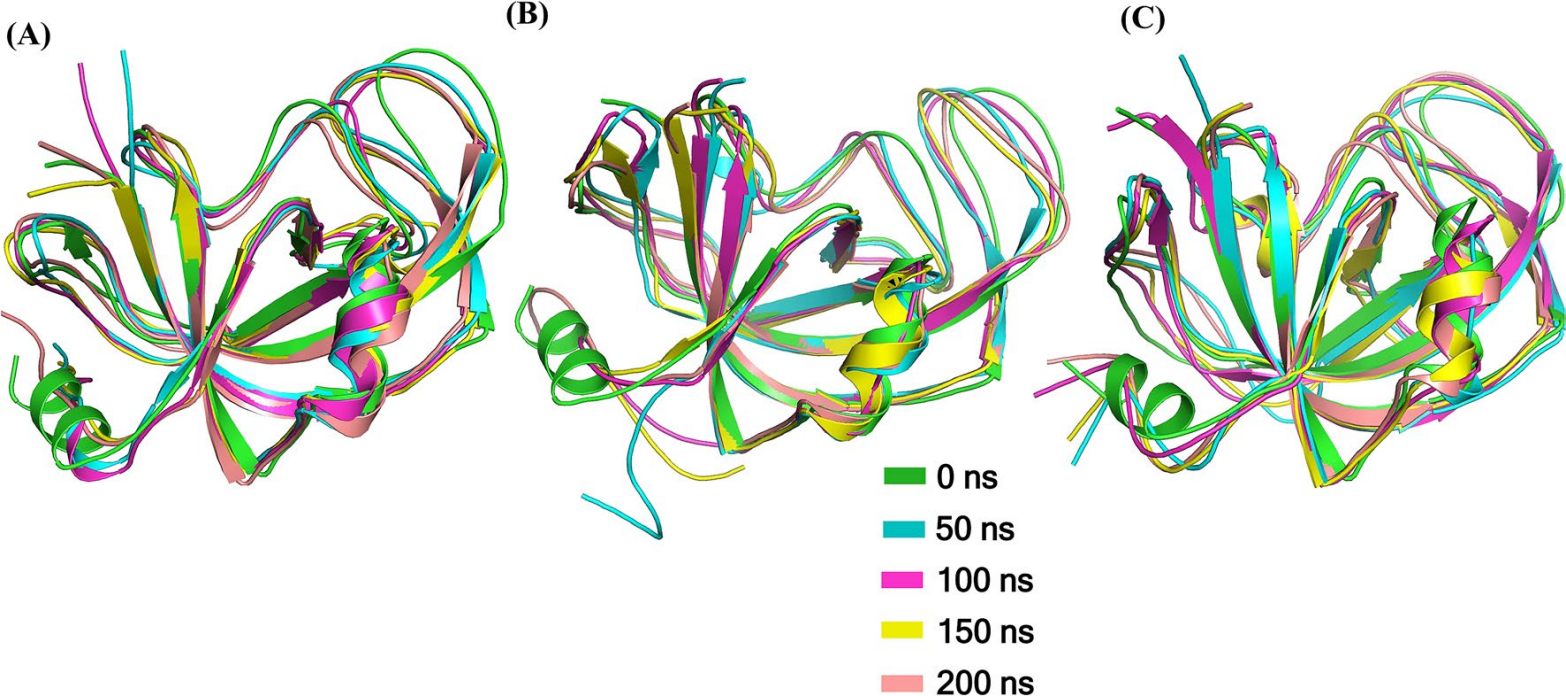
Structural snapshots of CTC1- (**A**) WT, (**B**) R806C, and (**C**) R806L at an interval of 50 ns from 0 to 200 ns of simulation. Structures were drawn using PyMOL (https://pymol.org/2).

MD simulation was utilized to study the disorderly impact of R806C and R806L on CTC1 conformation. The superimposed structural snapshots of CTC-WT, R806C and R806L taken at every 50 ns during the simulation are shown in Fig. 8. No significant difference is observed in mutants' structures when compared with WT except the loop regions, which become more flexible during the simulation in R806C. Interestingly, loss of the N-terminal helix was observed in both mutants during the early phase of the simulation, while it seems to disappear after 100 ns in the case of WT. Decrement was observed in the secondary structure content of mutants as compared to WT. A significant change was observed in the initial and final conformation of all three systems with RMSD calculated in PyMOL as 1.58 Å, 1.96 Å and 1.43 Å for CTC1-WT, R806C and R806L, respectively (Fig. 8).

**Figure 9:**
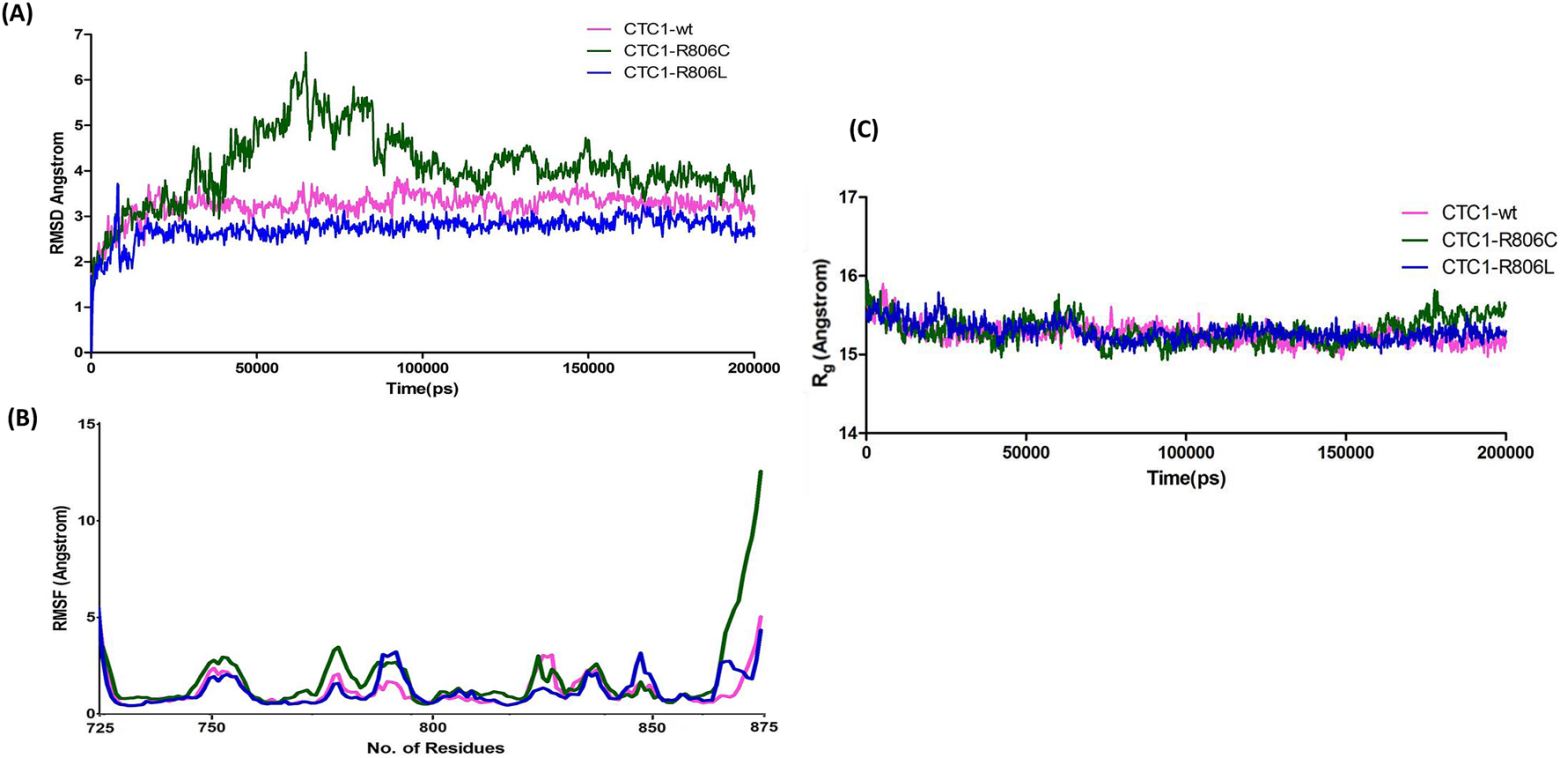
Conformational changes in CTC1 protein and its mutants. (**A**) Time evaluation of RMSD, (**B**) RMSF and (**C**) Radius of gyration of CTC1.

The time evaluation RMSD plot CTC1-WT, R806C, and R806L is represented in Fig. 9A. The result indicates a significant shift in the mutants' RMSD, especially in the case of R806C. We did not find any shift transition in the case of R806L while comparing with WT (Fig. 9A). It was observed that R806L was more stable and showed compactness with RMSD below ~ 3 Å, closely followed by WT (~ 3.5 Å RMSD). For R806C, the average RMSD was observed to be ~ 4 Å and the structure showing the unstable distribution of RMSD throughout the trajectory. The RMSD of R806C showed a sharp shift up to 6.5 Å suggesting unfolding transition of the CTC1 conformation upon mutation. During the RMSF analysis, the residual fluctuation pattern was similar in CTC1-WT and R806L, with two significant peaks in the 780–790 and 845–855 aa regions. While in the case of R806C, the residual fluctuation showed a higher tendency with a few random peaks in the 770–790, 820–830 and 865–875 aa regions (Fig. 9B).

To further investigate the compactness and stability of CTC1-WT and its mutants, we have calculated the Radius of gyration (*R*_*g*_) of all three systems and plotted as a function of time (Fig. 9C). Here we found that there is no significant difference in the average *R*_*g*_ values of WT, R806C and R806L. However, a slight increment can be observed after 160 ns in *R*_*g*_ in the case of R806C, suggesting a loss in compactness, as RMSD suggested (Fig. 9C).

## Conclusion

SNPs are considered as one of the most frequent genetic variants associated with several human diseases. Extensive analysis of SNPs can give insights to understand disease-causing mechanisms and help find effective treatments of diseases. In the present study, we have analyzed the nsSNPs of the CTC1, specifically the C-terminal OB-fold region. Sequence and structure-based analysis have shown that 126 mutations present in the C-terminal OB-fold of CTC1 where 75 mutations were found to be deleterious and destabilizing. A pathogenicity study revealed that 11 out of all the mutations are pathogenic. Although the RMSD calculation could not give any conclusive result, aggregation propensity analysis showed that almost 45% of the pathogenic mutations present in the C-terminal OB-fold of CTC1 tend to form aggregates or become less soluble. Pathogenicity of these mutations may occurred due to structural changes caused by the gain or loss of noncovalent intramolecular forces. MD simulation analyses, especially RMSD indicated a significant conformational loss in CTC1 protein structure due to R806C mutation. The results provide an in-depth understanding of the conformational behavior of CTC1 upon mutations. This study offers a detailed insight to understand the pathogenic nsSNPs of C-terminal OB-fold of CTC1 and the possible consequences of these mutations. These insights may be further used to build therapeutic strategies to cure CTC1 associated diseases.

## Supplementary Information

Supplementary Information

## References

[1] O'sullivan RJ, Karlseder J (2010). Telomeres: protecting chromosomes against genome instability. Nat. Rev. Mol. Cell Biol..

[2] Longhese MP (2008). DNA damage response at functional and dysfunctional telomeres. Genes Dev..

[3] Gorgoulis VG (2005). Activation of the DNA damage checkpoint and genomic instability in human precancerous lesions. Nature.

[4] Palm W, de Lange T (2008). How shelterin protects mammalian telomeres. Annu. Rev. Genet..

[5] Lipps HJ, Rhodes D (2009). G-quadruplex structures: in vivo evidence and function. Trends Cell Biol..

[6] di Fagagna FDA (2003). A DNA damage checkpoint response in telomere-initiated senescence. Nature.

[7] Aguado J (2019). Inhibition of DNA damage response at telomeres improves the detrimental phenotypes of Hutchinson-Gilford Progeria Syndrome. Nat. Commun..

[8] Lim, C. J. & Cech, T. R. Shaping human telomeres: from shelterin and CST complexes to telomeric chromatin organization. *Nature reviews Mol. Cell Biol.* **22**, 283–298 (2021). 10.1038/s41580-021-00328-y PMC8221230 33564154

[9] Amir M (2020). A systems view of the genome guardians: mapping the signaling circuitry underlying oligonucleotide/oligosaccharide-binding fold proteins. Omics: J. Integr. Biol..

[10] Amir M (2020). Structural features of nucleoprotein CST/shelterin complex involved in the telomere maintenance and its association with disease mutations. Cells.

[11] Stewart JA, Wang Y, Ackerson SM, Schuck PL (2018). Emerging roles of CST in maintaining genome stability and human disease. Front. Biosci. (Landmark Edn.).

[12] Lim CJ (2020). The structure of human CST reveals a decameric assembly bound to telomeric DNA. Science.

[13] Surovtseva YV (2009). Conserved telomere maintenance component 1 interacts with STN1 and maintains chromosome ends in higher eukaryotes. Mol. Cell.

[14] Feng X (2018). CTC1-STN1 terminates telomerase while STN1-TEN1 enables C-strand synthesis during telomere replication in colon cancer cells. Nat. Commun..

[15] Amir M (2020). Structure, function and therapeutic implications of OB-fold proteins: A lesson from past to present. Brief. Funct. Genom..

[16] Stewart JA (2012). Human CST promotes telomere duplex replication and general replication restart after fork stalling. EMBO J..

[17] Carrino S, Hennecker CD, Murrieta AC, Mittermaier A (2021). Frustrated folding of guanine quadruplexes in telomeric DNA. Nucleic Acids Res..

[18] Bhattacharjee A, Wang Y, Diao J, Price CM (2017). Dynamic DNA binding, junction recognition and G4 melting activity underlie the telomeric and genome-wide roles of human CST. Nucleic Acids Res..

[19] Lue NF (2018). Evolving linear chromosomes and telomeres: a C-strand-centric view. Trends Biochem. Sci..

[20] Gu P (2012). CTC1 deletion results in defective telomere replication, leading to catastrophic telomere loss and stem cell exhaustion. EMBO J..

[21] Price C (2010). Evolution of CST function in telomere maintenance. Cell Cycle.

[22] Amir M (2019). Investigating architecture and structure-function relationships in cold shock DNA-binding domain family using structural genomics-based approach. Int. J. Biol. Macromol..

[23] Lyu, X., Sang, P. B. & Chai, W. CST in maintaining genome stability: beyond telomeres. *DNA Repair* 103104 (2021). 10.1016/j.dnarep.2021.103104 PMC8081025 33780718

[24] Saint-Leandre B, Levine MT (2020). The telomere paradox: stable genome preservation with rapidly evolving proteins. Trends Genet..

[25] Huang C, Jia P, Chastain M, Shiva O, Chai W (2017). The human CTC1/STN1/TEN1 complex regulates telomere maintenance in ALT cancer cells. Exp. Cell Res..

[26] Wang Y, Chai W (2018). Pathogenic CTC1 mutations cause global genome instabilities under replication stress. Nucleic Acids Res..

[27] Grill, S. & Nandakumar, J. Molecular mechanisms of telomere biology disorders. *J. Biol. Chem. *100064 (2021). 10.1074/jbc.REV120.014017 PMC7948428 33482595

[28] Shastrula PK, Rice CT, Wang Z, Lieberman PM, Skordalakes EJNAR (2018). Structural and functional analysis of an OB-fold in human Ctc1 implicated in telomere maintenance and bone marrow syndromes. Nucleic Acids Res..

[29] Shastrula PK, Rice CT, Wang Z, Lieberman PM, Skordalakes E (2018). Structural and functional analysis of an OB-fold in human Ctc1 implicated in telomere maintenance and bone marrow syndromes. Nucleic Acids Res..

[30] Amir M (2016). Purification and characterization of oligonucleotide binding (OB)-fold protein from medicinal plant Tinospora cordifolia. J. Chromatogr. B.

[31] Amir M (2018). Sequence, structure and evolutionary analysis of cold shock domain proteins, a member of OB fold family. J. Evol. Biol..

[32] Roake CM, Artandi SE (2020). Regulation of human telomerase in homeostasis and disease. Nat. Rev. Mol. Cell Biol..

[33] Han E (2020). A unique case of coats plus syndrome and dyskeratosis congenita in a patient with CTC1 mutations. Ophthalmic Genet..

[34] Chen L-Y, Majerská J, Lingner J (2013). Molecular basis of telomere syndrome caused by CTC1 mutations. Genes Dev..

[35] Shen W (2019). Impact of germline ctc 1 alterations on telomere length in acquired bone marrow failure. Br. J. Haematol..

[36] Rice C, Skordalakes E (2016). Structure and function of the telomeric CST complex. Comput. Struct. Biotechnol. J..

[37] Gu P, Chang S (2013). Functional characterization of human CTC 1 mutations reveals novel mechanisms responsible for the pathogenesis of the telomere disease C oats plus. Aging Cell.

[38] Yates CM, Sternberg MJ (2013). The effects of non-synonymous single nucleotide polymorphisms (nsSNPs) on protein–protein interactions. J. Mol. Biol..

[39] Zheng, J., Guo, N. & Wagner, A. Selection enhances protein evolvability by increasing mutational robustness and foldability. *Science* **370**, 6521 (2020). 10.1126/science.abb5962 33273072

[40] Tokuriki N, Tawfik DS (2009). Stability effects of mutations and protein evolvability. Curr. Opin. Struct. Biol..

[41] Amir M (2019). Investigation of deleterious effects of nsSNPs in the POT1 gene: a structural genomics-based approach to understand the mechanism of cancer development. J. Cell. Biochem..

[42] Amir M (2019). Structural analysis and conformational dynamics of STN1 gene mutations involved in coat plus syndrome. Front. Mol. Biosci..

[43] Amir, M. *et al.* Impact of Gln94Glu mutation on the structure and function of protection of telomere 1, a cause of cutaneous familial melanoma. *J. Biomol. Struct. Dyn.* **38**(5), 1514–1524 (2019). 10.1080/07391102.2019.1610500 31014199

[44] Sherry ST (2001). dbSNP: the NCBI database of genetic variation. Nucleic Acids Res..

[45] Stenson PD (2009). The human gene mutation database: 2008 update. Genome Med..

[46] Landrum MJ (2014). ClinVar: public archive of relationships among sequence variation and human phenotype. Nucleic Acids Res..

[47] Hubbard T (2002). The Ensembl genome database project. Nucleic Acids Res..

[48] Kumar P, Henikoff S, Ng PC (2009). Predicting the effects of coding non-synonymous variants on protein function using the SIFT algorithm. Nat. Protoc..

[49] Ramensky V, Bork P, Sunyaev S (2002). Human non-synonymous SNPs: server and survey. Nucleic Acids Res..

[50] Choi Y, Chan AP (2015). PROVEAN web server: a tool to predict the functional effect of amino acid substitutions and indels. Bioinformatics.

[51] Reva B, Antipin Y, Sander C (2011). Predicting the functional impact of protein mutations: application to cancer genomics. Nucleic Acids Res..

[52] Niroula A, Urolagin S, Vihinen M (2015). PON-P2: prediction method for fast and reliable identification of harmful variants. PLoS ONE.

[53] Quan L, Lv Q, Zhang Y (2016). STRUM: structure-based prediction of protein stability changes upon single-point mutation. Bioinformatics.

[54] Laimer J, Hofer H, Fritz M, Wegenkittl S, Lackner P (2015). MAESTRO-multi agent stability prediction upon point mutations. BMC Bioinform..

[55] Pandurangan AP, Ochoa-Montaño B, Ascher DB, Blundell TL (2017). SDM: a server for predicting effects of mutations on protein stability. Nucleic Acids Res..

[56] Pires DE, Ascher DB, Blundell TL (2014). mCSM: predicting the effects of mutations in proteins using graph-based signatures. Bioinformatics.

[57] Pires DE, Ascher DB, Blundell TL (2014). DUET: a server for predicting effects of mutations on protein stability using an integrated computational approach. Nucleic Acids Res..

[58] López-Ferrando V, Gazzo A, De La Cruz X, Orozco M, Gelpí JL (2017). PMut: a web-based tool for the annotation of pathological variants on proteins, 2017 update. Nucleic Acids Res..

[59] Pejaver V (2020). Inferring the molecular and phenotypic impact of amino acid variants with MutPred2. Nat. Commun..

[60] Ausaf Ali S, Hassan I, Islam A, Ahmad F (2014). A review of methods available to estimate solvent-accessible surface areas of soluble proteins in the folded and unfolded states. Curr. Protein Peptide Sci..

[61] Paladin L, Piovesan D, Tosatto SC (2017). SODA: prediction of protein solubility from disorder and aggregation propensity. Nucleic Acids Res..

[62] Jubb HC (2017). Arpeggio: a web server for calculating and visualising interatomic interactions in protein structures. J. Mol. Biol..

[63] Ashkenazy H (2016). ConSurf 2016: an improved methodology to estimate and visualize evolutionary conservation in macromolecules. Nucleic Acids Res..

[64] Mohammad T, Dahiya R, Hassan MI (2020). Systems and Synthetic Immunology.

[65] Mohammad T (2019). Identification and evaluation of bioactive natural products as potential inhibitors of human microtubule affinity-regulating kinase 4 (MARK4). J. Biomol. Struct. Dyn..

[66] Naqvi AA, Mohammad T, Hasan GM, Hassan M (2018). Advancements in docking and molecular dynamics simulations towards ligand-receptor interactions and structure-function relationships. Curr. Top. Med. Chem..

[67] Amir, M. *et al.* Impact of Gln94Glu mutation on the structure and function of protection of telomere 1, a cause of cutaneous familial melanoma. *J. Biomol. Struct. Dyn.* **38**(5), 1514–1524 (2019). 10.1080/07391102.2019.1610500 31014199

[68] Mohammad T (2020). Impact of amino acid substitution in the kinase domain of Bruton tyrosine kinase and its association with X-linked agammaglobulinemia. Int. J. Biol. Macromol..

[69] Amir, M. *et al.* Structural and functional impact of non-synonymous SNPs in the CST complex subunit TEN1: structural genomics approach. *Biosci. Rep.*** 39**, BSR20190312 (2019). 10.1042/BSR20190312 PMC6522806 31028137

[70] Freyssin A, Page G, Fauconneau B, Bilan AR (2018). Natural polyphenols effects on protein aggregates in Alzheimer's and Parkinson's prion-like diseases. Neural Regen. Res..

[71] Nazam F (2021). Mechanistic insights into the pathogenesis of neurodegenerative diseases: towards the development of effective therapy. Mol. Cell Biochem..

[72] Sami N (2017). Protein aggregation, misfolding and consequential human neurodegenerative diseases. Int. J. Neurosci..

[73] Sami N (2017). Exploring missense mutations in tyrosine kinases implicated with neurodegeneration. Mol. Neurobiol..

[74] Kumar V, Islam A, Hassan MI, Ahmad F (2016). Delineating the relationship between amyotrophic lateral sclerosis and frontotemporal dementia: Sequence and structure-based predictions. Biochim. Biophys. Acta.

[75] Kumar V, Islam A, Hassan MI, Ahmad F (2016). Therapeutic progress in amyotrophic lateral sclerosis-beginning to learning. Eur. J. Med. Chem..

[76] Kumar V, Islam A, Hassan MI, Ahmad F (2016). (2016) Delineating the relationship between amyotrophic lateral sclerosis and frontotemporal dementia: sequence and structure-based predictions. Biochim Biophys Acta (BBA) Mol. Basis Dis..

[77] Kumar V, Prakash A, Pandey P, Lynn AM, Hassan MI (2018). TFE-induced local unfolding and fibrillation of SOD1: bridging the experiment and simulation studies. Biochem. J..

[78] Kumar V (2016). Protein aggregation and neurodegenerative diseases: From theory to therapy. Eur. J. Med. Chem..

