# Supplementary material for: Structural genomics approach to investigate deleterious impact of nsSNPs in conserved telomere maintenance component 1
Source: Sci Rep. 2021 May 13;11:10202. doi: 10.1038/s41598-021-89450-7 (PMC8119478; doi:10.1038/s41598-021-89450-7)
Supplement: Supplementary file 1 — Supplementary Information [file 41598_2021_89450_MOESM1_ESM.docx]

***Supplementary Information***

**Structural Genomics Approach to Investigate Deleterious Impact of nsSNPs in Conserved Telomere Maintenance Component 1**

Arunabh Choudhury^1,#^, Taj Mohammad^2,#^ Nikhil Samarth^3^, Afzal Hussain^4^, Md. Tabish Rehman^4^, Asimul Islam^2^, Mohamed F. Alajmi^4^, Shailza Singh^3^ and Md. Imtaiyaz Hassan^2,*^

*^1^Department of Computer Science, Jamia Millia Islamia, Jamia Nagar, New Delhi – 110025, India.*

*^2^Center for Interdisciplinary Research in Basic Sciences, Jamia Millia Islamia, Jamia Nagar, New Delhi – 110025, India.*

*^3^National Centre for Cell Science, NCCS Complex, Ganeshkhind, SP Pune University,*

*Campus, Pune-411007, INDIA*

*^4^Department of Pharmacognosy, College of Pharmacy, King Saud University, Riyadh 11451, Saudi Arabia.*

*^#^These authors contributed equally to this work*

*^*^Corresponding author:*

**Md. Imtaiyaz Hassan, Ph.D., FRSB, FRSC.**

Assistant Professor

*Centre for Interdisciplinary Research in Basic Sciences*

*Jamia Millia Islamia, New Delhi-110025, India*

*Email:* [*mihassan@jmi.ac.in*](mailto:mihassan@jmi.ac.in)

**Supplementary Table S1:** Sequence based analysis of CTC1 mutations.

| **S. No.** | **AA**  **Substitution** | **SI​FT** | **Poly­Phen** | **PROVEAN Score** | **PROVEAN Prediction** | **M A FI score** | **M A Impact** | **Probability of**  **Pathogenicity**  **(PON-P2)** | **Standard Error** | **Prediction**  **(PON-P2)** |
| --- | --- | --- | --- | --- | --- | --- | --- | --- | --- | --- |
|  | A2E | 0.16 | 0.023 | 0.357 | Neutral | 1.735 | low | 0.062 | 0.024 | Neutral |
|  | A2T | 4.32 | 0.007 | -0.226 | Neutral | 1.735 | low | 0.081 | 0.034 | Neutral |
|  | A3V | 0.22 | 0.025 | -0.672 | Neutral | 1.32 | low | 0.187 | 0.055 | Neutral |
|  | A3G | 0 | 0.002 | -0.126 | Neutral | 1.67 | low | 0.208 | 0.083 | Unknown |
|  | A3S | 0.16 | 0.025 | -0.021 | Neutral | 1.32 | low | 0.261 | 0.089 | Unknown |
|  | A3T | 0.04 | 0.23 | -0.305 | Neutral | 1.67 | low | 0.142 | 0.039 | Neutral |
|  | R5Q | 0.66 | 0.003 | -0.996 | Neutral | 0.345 | neutral | 0.19 | 0.05 | Neutral |
|  | R5W | 0.19 | 0.555 | -2.328 | Neutral | 1.04 | low | 0.355 | 0.101 | Unknown |
|  | R5G | 0.64 | 0.001 | -1.698 | Neutral | 0.145 | neutral | 0.081 | 0.042 | Neutral |
|  | A6V | 0.14 | 0.025 | -1.054 | Neutral | 1.735 | low | 0.126 | 0.039 | Neutral |
|  | A6S | 0.16 | 0.297 | -0.686 | Neutral | 1.39 | low | 0.184 | 0.083 | Unknown |
|  | A6P | 0.26 | 0.015 | -1.152 | Neutral | 1.735 | low | 0.188 | 0.05 | Neutral |
|  | A6T | 0.06 | 0.372 | -0.708 | Neutral | 1.735 | low | 0.194 | 0.066 | Neutral |
|  | Q7H | 0.08 | 0.371 | -0.899 | Neutral | 1.59 | low | 0.117 | 0.031 | Neutral |
|  | Q7R | 0.54 | 0.001 | -0.893 | Neutral | 0.9 | low | 0.064 | 0.027 | Neutral |
|  | V8G | 0.2 | 0.04 | -0.808 | Neutral | 0.345 | neutral | 0.159 | 0.042 | Neutral |
|  | V8I | 0.3 | 0.056 | -0.296 | Neutral | 0 | neutral | 0.11 | 0.037 | Neutral |
|  | P9L | 0.03 | 0.003 | -1.527 | Neutral | 1.1 | low | 0.414 | 0.121 | Unknown |
|  | P9R | 0.02 | 0.316 | -1.699 | Neutral | 1.445 | low | 0.206 | 0.06 | Neutral |
|  | P9H | 0.05 | 0.017 | -1.324 | Neutral | 1.445 | low | 0.181 | 0.043 | Neutral |
|  | P9S | 0.82 | 0.012 | -1.116 | Neutral | 1.1 | low | 0.166 | 0.061 | Neutral |
|  | S10F | 0.06 | 0.078 | -0.901 | Neutral | 1.04 | low | 0.16 | 0.048 | Neutral |
|  | S10C | 0.09 | 0.325 | -0.586 | Neutral | 1.04 | low | 0.133 | 0.035 | Neutral |
|  | S10P | 0.28 | 0.065 | -0.565 | Neutral | 0.69 | neutral | 0.237 | 0.056 | Neutral |
|  | S10T | 0.73 | 0 | -0.456 | Neutral | 0 | neutral | 0.083 | 0.034 | Neutral |
|  | S11P | 0.06 | 0.61 | -0.868 | Neutral | 1.795 | low | 0.489 | 0.086 | Unknown |
|  | S11F | 0.41 | 0.692 | -1.354 | Neutral | 1.795 | low | 0.176 | 0.045 | Neutral |
|  | E12K | 0 | 0.996 | -1.742 | Neutral | 1.895 | low | 0.539 | 0.115 | Unknown |
|  | Q13E | 0.17 | 0.009 | -1.465 | Neutral | 1.3 | low | 0.175 | 0.045 | Neutral |
|  | L16P | 0 | 0.999 | -4.079 | Deleterious | 1.895 | low | 0.833 | 0.043 | Pathogenic |
|  | L16V | 0 | 0.994 | -1.598 | Neutral | 1.895 | low | 0.262 | 0.086 | Unknown |
|  | E17G | 0 | 0.714 | -3.269 | Deleterious | 1.895 | low | 0.235 | 0.061 | Unknown |
|  | E17K | 0.05 | 0.046 | -1.623 | Neutral | 1.895 | low | 0.301 | 0.066 | Unknown |
|  | D18V | 0.2 | 0 | -1.576 | Neutral | -0.345 | neutral | 0.068 | 0.026 | Neutral |
|  | Q20R | 0.47 | 0.006 | -0.37 | Neutral | 1.5 | low | 0.116 | 0.038 | Neutral |
|  | V21G | 0.09 | 0.01 | -1.333 | Neutral | 0 | neutral | 0.173 | 0.062 | Neutral |
|  | K25N | 0.02 | 0.286 | -0.957 | Neutral | 0.345 | neutral | 0.209 | 0.042 | Neutral |
|  | L27P | 0 | 0.958 | -3.163 | Deleterious | 1.895 | low | 0.729 | 0.101 | Unknown |
|  | L27V | 0.01 | 0.324 | -1.117 | Neutral | 1.895 | low | 0.234 | 0.065 | Unknown |
|  | A30D | 0.01 | 0.238 | -1.237 | Neutral | 1.24 | low | 0.209 | 0.06 | Neutral |
|  | K32N | 0.04 | 0.124 | -1.277 | Neutral | 1.67 | low | 0.097 | 0.028 | Neutral |
|  | K32R | 0.06 | 0.124 | -0.859 | Neutral | 1.67 | low | 0.036 | 0.014 | Neutral |
|  | P34S | 0.1 | 0.01 | -2.418 | Neutral | 0.695 | neutral | 0.148 | 0.042 | Neutral |
|  | N35S | 0.47 | 0.002 | -0.718 | Neutral | 0.46 | neutral | 0.134 | 0.038 | Neutral |
|  | V36A | 0.67 | 0.007 | -1.048 | Neutral | 1.67 | low | 0.199 | 0.045 | Neutral |
|  | Q37R | 0.05 | 0.011 | -1.195 | Neutral | 1.445 | low | 0.226 | 0.052 | Neutral |
|  | Q37K | 0.02 | 0.124 | -0.972 | Neutral | 1.795 | low | 0.178 | 0.064 | Neutral |
|  | P40L | 0.09 | 0.025 | -2.701 | Deleterious | 0 | neutral | 0.221 | 0.064 | Unknown |
|  | V42I | 0.12 | 0.04 | -0.405 | Neutral | 1.895 | low | 0.163 | 0.06 | Neutral |
|  | I43T | 0 | 0.714 | -2.147 | Neutral | 1.845 | low | 0.539 | 0.067 | Unknown |
|  | I43V | 0.16 | 0.028 | -0.358 | Neutral | 1.845 | low | 0.142 | 0.058 | Neutral |
|  | D44G | 0.01 | 0.359 | -2.41 | Neutral | 1.845 | low | 0.25 | 0.057 | Unknown |
|  | C45G | 0.01 | 0.98 | -4.89 | Deleterious | 1.895 | low | 0.557 | 0.071 | Unknown |
|  | K47E | 0.01 | 0.811 | -1.24 | Neutral | 1.895 | low | 0.321 | 0.077 | Unknown |
|  | T48I | 0.03 | 0.625 | -1.895 | Neutral | 1.735 | low | 0.104 | 0.055 | Neutral |
|  | T48A | 0.24 | 0.019 | -1.161 | Neutral | 1.735 | low | 0.075 | 0.025 | Neutral |
|  | V49L | 0.26 | 0.003 | -0.819 | Neutral | 1.15 | low | 0.07 | 0.024 | Neutral |
|  | W50C | 0.11 | 0.106 | -4.333 | Deleterious | 1.895 | low | 0.618 | 0.071 | Unknown |
|  | W50L | 0 | 0.66 | -3.946 | Deleterious | 1.895 | low | 0.313 | 0.059 | Unknown |
|  | W50G | 0.04 | 0.578 | -3.623 | Deleterious | 1.895 | low | 0.586 | 0.058 | Unknown |
|  | L51F | 0.07 | 0.839 | -1.481 | Neutral | 1.895 | low | 0.116 | 0.039 | Neutral |
|  | N56H | 0.1 | 0.007 | -1.528 | Neutral | 0.955 | low | 0.131 | 0.053 | Neutral |
|  | Q57R | 0.11 | 0.586 | -1.325 | Neutral | 1.895 | low | 0.141 | 0.038 | Neutral |
|  | L63F | 0.03 | 0.86 | -1.983 | Neutral | 2.24 | medium | 0.313 | 0.06 | Unknown |
|  | Y65C | 0 | 0.999 | -4.76 | Deleterious | 2.645 | medium | 0.713 | 0.07 | Unknown |
|  | S66G | 0.01 | 0.99 | -2.128 | Neutral | 2.645 | medium | 0.411 | 0.065 | Unknown |
|  | F67L | 0.08 | 0.996 | -2.687 | Deleterious | 2.3 | medium | 0.211 | 0.055 | Neutral |
|  | V68I | 0.6 | 0.48 | -0.356 | Neutral | 2.105 | medium | 0.292 | 0.089 | Unknown |
|  | V70A | 0.07 | 0.043 | -2.526 | Deleterious | 1.81 | low | 0.239 | 0.06 | Unknown |
|  | V70I | 0.61 | 0.457 | -0.166 | Neutral | 1.81 | low | 0.22 | 0.068 | Unknown |
|  | L73V | 0 | 0.996 | -1.814 | Neutral | 2.67 | medium | 0.348 | 0.061 | Unknown |
|  | K74N | 0.01 | 0.206 | -1.871 | Neutral | 1.24 | low | 0.144 | 0.041 | Neutral |
|  | K74R | 0.95 | 0 | -0.343 | Neutral | -1.11 | neutral | 0.058 | 0.021 | Neutral |
|  | T75N | 0.1 | 0.563 | -1.167 | Neutral | 2.38 | medium | 0.413 | 0.076 | Unknown |
|  | H76Q | 0.95 | 0.022 | -2.048 | Neutral | 1.545 | low | 0.181 | 0.044 | Neutral |
|  | R78H | 0.58 | 0.003 | 0.664 | Neutral | -0.43 | neutral | 0.072 | 0.025 | Neutral |
|  | R78C | 0.13 | 0.653 | -3.288 | Deleterious | 1.67 | low | 0.648 | 0.116 | Unknown |
|  | C81S | 0 | 0.998 | -5.333 | Deleterious | 2.67 | medium | 0.781 | 0.056 | Pathogenic |
|  | C81R | 0 | 0.999 | -6.638 | Deleterious | 2.67 | medium | 0.853 | 0.043 | Pathogenic |
|  | S83T | 0 | 0.996 | -1.862 | Neutral | 2.67 | medium | 0.371 | 0.073 | Unknown |
|  | S83N | 0 | 0.998 | -1.767 | Neutral | 2.67 | medium | 0.657 | 0.084 | Unknown |
|  | S86L | 0 | 0.773 | -2.914 | Deleterious | 2.555 | medium | 0.56 | 0.07 | Unknown |
|  | S89G | 0.05 | 0.442 | -2.3 | Neutral | 2.52 | medium | 0.38 | 0.069 | Unknown |
|  | S90G | 0.03 | 0.168 | -1.908 | Neutral | 1.935 | low | 0.111 | 0.039 | Neutral |
|  | S90C | 0.45 | 0.067 | -2.535 | Deleterious | 1.935 | low | 0.133 | 0.047 | Neutral |
|  | Y92C | 0.02 | 0.982 | -4.776 | Deleterious | 2.615 | medium | 0.803 | 0.059 | Pathogenic |
|  | Q93R | 0.03 | 0.042 | -1.168 | Neutral | 2.19 | medium | 0.208 | 0.048 | Neutral |
|  | A96V | 0.18 | 0.067 | -1.714 | Neutral | 2.43 | medium | 0.268 | 0.086 | Unknown |
|  | A96T | 0.58 | 0.06 | -1.216 | Neutral | 1.395 | low | 0.159 | 0.055 | Neutral |
|  | A96S | 0.17 | 0.359 | -0.912 | Neutral | 2.085 | medium | 0.207 | 0.072 | Unknown |
|  | Q97R | 0.33 | 0.003 | -1.275 | Neutral | 1.64 | low | 0.276 | 0.062 | Unknown |
|  | P105H | 0.02 | 0.912 | -3.774 | Deleterious | 2.34 | medium | 0.287 | 0.049 | Unknown |
|  | P105S | 0.23 | 0.563 | -2.454 | Neutral | 1.995 | medium | 0.131 | 0.041 | Neutral |
|  | P107L | 0 | 0.675 | -5.286 | Deleterious | 2.645 | medium | 0.396 | 0.091 | Unknown |
|  | P107R | 0 | 0.858 | -4.776 | Deleterious | 2.645 | medium | 0.428 | 0.078 | Unknown |
|  | R108Q | 0.18 | 0.049 | -1.61 | Neutral | 1.905 | low | 0.171 | 0.05 | Neutral |
|  | R108G | 0 | 0.823 | -3.948 | Deleterious | 2.595 | medium | 0.5 | 0.079 | Unknown |
|  | E109K | 0.07 | 0.942 | -1.578 | Neutral | 2.555 | medium | 0.542 | 0.084 | Unknown |
|  | L111H | 0.04 | 0.151 | -4.233 | Deleterious |  |  | 0.778 | 0.05 | Pathogenic |
|  | L111P | 0.06 | 0.149 | -3.948 | Deleterious | 2.67 | medium | 0.91 | 0.03 | Pathogenic |
|  | L113F | 0.04 | 0.073 | -2.514 | Deleterious | 2.67 | medium | 0.261 | 0.069 | Unknown |
|  | L114R | 0.36 | 0 | -3.717 | Deleterious |  |  | 0.673 | 0.072 | Unknown |
|  | G115R | 0 | 1 | -5.267 | Deleterious | 2.67 | medium | 0.693 | 0.073 | Unknown |
|  | T116I | 0 | 0.157 | -1.629 | Neutral | 2.125 | medium | 0.249 | 0.066 | Unknown |
|  | T116S | 0 | 0.026 | -1.681 | Neutral | 2.47 | medium | 0.319 | 0.119 | Unknown |
|  | T116A | 0 | 0.009 | -1.905 | Neutral | 2.47 | medium | 0.319 | 0.084 | Unknown |
|  | T118R | 0 | 0.925 | -3.184 | Deleterious | 2.585 | medium | 0.62 | 0.092 | Unknown |
|  | D119V | 0 | 1 | -5.014 | Deleterious | 2.645 | medium | 0.56 | 0.095 | Unknown |
|  | S121L | 0.21 | 0.001 | -2.092 | Neutral | 0.785 | neutral | 0.059 | 0.022 | Neutral |
|  | D123Y | 0.03 | 0.912 | -4.024 | Deleterious | 2.32 | medium | 0.363 | 0.092 | Unknown |
|  | Q126K | 0.1 | 0.297 | -1.805 | Neutral | 2.52 | medium | 0.111 | 0.037 | Neutral |
|  | E127G | 0.02 | 0.01 | -3.221 | Deleterious | 1.755 | low | 0.17 | 0.047 | Neutral |
|  | C128F | 0.66 | 0.003 | -2.531 | Deleterious | 2.34 | medium | 0.22 | 0.059 | Neutral |
|  | G131R | 0 | 1 | -4.838 | Deleterious | 2.67 | medium | 0.864 | 0.033 | Pathogenic |
|  | S132N | 0.63 | 0.003 | -1.544 | Neutral | 1.485 | low | 0.183 | 0.042 | Neutral |
|  | L133H | - | 0 | -4.567 | Deleterious | 2.645 | medium | 0.761 | 0.066 | Unknown |
|  | N138S | 0.04 | 0.359 | -2.5 | Deleterious | 2.615 | medium | 0.573 | 0.114 | Unknown |
|  | T139A | 0 | 0.51 | -2.333 | Neutral | 2.555 | medium | 0.317 | 0.056 | Unknown |
|  | G140D | 0.07 | 0.999 | -3.995 | Deleterious | 2.67 | medium | 0.866 | 0.041 | Pathogenic |
|  | V141I | 1 | 0 | -0.181 | Neutral | -0.315 | neutral | 0.033 | 0.019 | Neutral |
|  | S143R | 0.11 | 0 | -1.53 | Neutral | -0.345 | neutral | 0.256 | 0.062 | Unknown |
|  | S143I | 0.05 | 0.038 | -1.576 | Neutral | 0 | neutral | 0.274 | 0.074 | Unknown |
|  | I147V | 0.03 | 0.197 | -0.51 | Neutral | 2.085 | medium | 0.226 | 0.053 | Neutral |
|  | D148E | 0.05 | 0.768 | -1.062 | Neutral | 2.505 | medium | 0.167 | 0.049 | Neutral |
|  | D150N | 0.03 | 0.218 | -1.786 | Neutral | 2.24 | medium | 0.261 | 0.058 | Unknown |
|  | L151F | 0.06 | 0.944 | -1.895 | Neutral | 2.615 | medium | 0.153 | 0.042 | Neutral |
|  | L151I | 0.03 | 0.822 | -0.686 | Neutral | 2.27 | medium | 0.146 | 0.042 | Neutral |
|  | L157P | 0 | 0.958 | -4.129 | Deleterious | 2.3 | medium | 0.864 | 0.051 | Pathogenic |
|  | L157V | 0 | 0.553 | -1.141 | Neutral | 2.645 | medium | 0.546 | 0.096 | Unknown |
|  | F160L | 0 | 0.99 | -3.105 | Deleterious | 2.3 | medium | 0.574 | 0.104 | Unknown |
|  | R162H | 0.01 | 0.157 | -0.284 | Neutral | 0 | neutral | 0.228 | 0.047 | Neutral |
|  | R162C | 0 | 0.365 | -0.492 | Neutral | -0.345 | neutral | 0.259 | 0.056 | Unknown |
|  | W163R | 0 | 0.999 | -8.371 | Deleterious | 2.67 | medium | 0.811 | 0.05 | Pathogenic |
|  | S164N | 0 | 0.996 | -0.957 | Neutral | 2.27 | medium | 0.229 | 0.045 | Neutral |
|  | P167L | 0 | 0.084 | -4.571 | Deleterious | 2.485 | medium | 0.611 | 0.075 | Unknown |
|  | P167S | 0 | 0.92 | -3.881 | Deleterious | 2.485 | medium | 0.554 | 0.067 | Unknown |
|  | P168H | 0.09 | 0.043 | -3.595 | Deleterious | 2.205 | medium | 0.322 | 0.05 | Unknown |
|  | P168S | 0.11 | 0.031 | -2.744 | Deleterious | 2.005 | medium | 0.188 | 0.051 | Neutral |
|  | A169T | 0.3 | 0.117 | -1.306 | Neutral | 2.155 | medium | 0.17 | 0.06 | Neutral |
|  | R170S | 0.12 | 0.009 | -1.402 | Neutral | 1.87 | low | 0.212 | 0.055 | Neutral |
|  | N172S | 0.18 | 0.005 | -1.687 | Neutral | 0.75 | neutral | 0.073 | 0.036 | Neutral |
|  | G175R | 0.26 | 0.044 | -2.06 | Neutral | 2.42 | medium | 0.22 | 0.051 | Neutral |
|  | H178R | 0.01 | 0.996 | -4.171 | Deleterious | 2.645 | medium | 0.515 | 0.072 | Unknown |
|  | D183Y | 0.27 | 0.465 | -3.014 | Deleterious | 0.69 | neutral | 0.257 | 0.065 | Unknown |
|  | D183N | 0.5 | 0.06 | -1.284 | Neutral | 0.69 | neutral | 0.082 | 0.034 | Neutral |
|  | A184P | 0.22 | 0.007 | -0.673 | Neutral | 1.1 | low | 0.094 | 0.032 | Neutral |
|  | P185A | 0 | 0.854 | -4.219 | Deleterious | 2.67 | medium | 0.36 | 0.093 | Unknown |
|  | V186L | 0.01 | 0.114 | -1.706 | Neutral | 2.565 | medium | 0.194 | 0.065 | Neutral |
|  | V186M | 0.01 | 0.192 | -1.673 | Neutral | 1.875 | low | 0.209 | 0.052 | Neutral |
|  | V188M | 0 | 0.999 | -1.386 | Neutral | 2.615 | medium | 0.539 | 0.081 | Unknown |
|  | P190R | 0 | 0.939 | -4.11 | Deleterious | 2.3 | medium | 0.588 | 0.075 | Unknown |
|  | P190A | 0.03 | 0.71 | -3.552 | Deleterious | 2.645 | medium | 0.255 | 0.098 | Unknown |
|  | T192I | 0.01 | 0.033 | -1.995 | Neutral | 2.3 | medium | 0.224 | 0.104 | Unknown |
|  | T192A | 0.16 | 0.085 | -0.848 | Neutral | 1.95 | medium | 0.142 | 0.056 | Neutral |
|  | I193L | 0.27 | 0.005 | -0.571 | Neutral | 1.735 | low | 0.153 | 0.052 | Neutral |
|  | P195L | 0.02 | 0.018 | -4.071 | Deleterious | 2.52 | medium | 0.424 | 0.074 | Unknown |
|  | P197A | 0.05 | 0.269 | -3.029 | Deleterious | 2.615 | medium | 0.167 | 0.098 | Unknown |
|  | V198L | 0.71 | 0 | 0.043 | Neutral | -1.59 | neutral | 0.045 | 0.025 | Neutral |
|  | V198I | 0.39 | 0.01 | -0.167 | Neutral | 0.69 | neutral | 0.062 | 0.032 | Neutral |
|  | T199M | 0.06 | 0.742 | -2.194 | Neutral | 2.47 | medium | 0.29 | 0.087 | Unknown |
|  | T199R | 0.14 | 0.789 | -2.437 | Neutral | 2.47 | medium | 0.282 | 0.063 | Unknown |
|  | T199P | 0.09 | 0.735 | -2.552 | Deleterious | 2.47 | medium | 0.281 | 0.048 | Neutral |
|  | P200L | 0.01 | 0.936 | -3.381 | Deleterious | 2.585 | medium | 0.247 | 0.074 | Unknown |
|  | P200S | 0.32 | 0.457 | -2.79 | Deleterious | 2.585 | medium | 0.389 | 0.082 | Unknown |
|  | P206A | 0.01 | 0.633 | -3.552 | Deleterious | 2.645 | medium | 0.136 | 0.038 | Neutral |
|  | E207D | 0.19 | 0.091 | -1.29 | Neutral | 2.42 | medium | 0.158 | 0.045 | Neutral |
|  | S208R | 0.5 | 0.025 | -1.268 | Neutral | 0.895 | low | 0.245 | 0.075 | Unknown |
|  | R214S | 0.45 | 0.007 | -0.711 | Neutral | 2.34 | medium | 0.107 | 0.036 | Neutral |
|  | R216K | 0.49 | 0.01 | -0.594 | Neutral | 2.565 | medium | 0.185 | 0.044 | Neutral |
|  | L219P | 0.21 | 0.011 | -1.714 | Neutral | 1.185 | low | 0.147 | 0.046 | Neutral |
|  | V222M | 0.13 | 0.132 | -0.124 | Neutral | 1.61 | low | 0.197 | 0.055 | Neutral |
|  | R224Q | 0.59 | 0.024 | -0.264 | Neutral | -0.69 | neutral | 0.151 | 0.041 | Neutral |
|  | A227V | 0.01 | 0.767 | -1.371 | Neutral | 2.615 | medium | 0.563 | 0.078 | Unknown |
|  | A227S | 0.02 | 0.885 | -0.91 | Neutral | 2.615 | medium | 0.325 | 0.064 | Unknown |
|  | A227T | 0.05 | 0.607 | -1.11 | Neutral | 2.27 | medium | 0.527 | 0.097 | Unknown |
|  | S229R | 0.15 | 0.007 | 0.378 | Neutral | 0.345 | neutral | 0.253 | 0.073 | Unknown |
|  | R232L | 0.01 | 0.429 | -2.501 | Deleterious | 2.42 | medium | 0.403 | 0.066 | Unknown |
|  | R232Q | 0.06 | 0.078 | -1.201 | Neutral | 2.42 | medium | 0.284 | 0.062 | Unknown |
|  | S234I | 0.01 | 0.891 | -2.962 | Deleterious | 2.535 | medium | 0.573 | 0.072 | Unknown |
|  | Q241E | 0.33 | 0.005 | -0.737 | Neutral | 1.245 | low | 0.101 | 0.027 | Neutral |
|  | A243V | 0.02 | 0.373 | -0.914 | Neutral | 2.08 | medium | 0.07 | 0.029 | Neutral |
|  | I246M | 0.05 | 0.664 | -0.663 | Neutral | 2.095 | medium | 0.158 | 0.045 | Neutral |
|  | I246V | 0.93 | 0.009 | -0.221 | Neutral | 0.485 | neutral | 0.066 | 0.027 | Neutral |
|  | I246L | 0.29 | 0.012 | -0.679 | Neutral | 1.4 | low | 0.153 | 0.053 | Neutral |
|  | L247P | 0 | 0.94 | -3.21 | Deleterious | 2.43 | medium | 0.808 | 0.08 | Unknown |
|  | S248C | 0.12 | 0.099 | -1.647 | Neutral | 2.24 | medium | 0.282 | 0.067 | Unknown |
|  | S252L | 0.69 | 0.017 | -1.541 | Neutral | 0.695 | neutral | 0.088 | 0.031 | Neutral |
|  | S252A | 0.85 | 0.281 | -0.724 | Neutral | 2.14 | medium | 0.066 | 0.027 | Neutral |
|  | P254R | 0.03 | 0.481 | -1.307 | Neutral | 2.3 | medium | 0.118 | 0.041 | Neutral |
|  | P254A | 0.19 | 0.02 | -1.124 | Neutral | 2.3 | medium | 0.06 | 0.024 | Neutral |
|  | A255S | 0.35 | 0.177 | -0.67 | Neutral | 1.63 | low | 0.165 | 0.044 | Neutral |
|  | A255T | 0.08 | 0.044 | -1.288 | Neutral | 1.63 | low | 0.124 | 0.047 | Neutral |
|  | T257I | 0.1 | 0.061 | -1.393 | Neutral | 1.355 | low | 0.049 | 0.019 | Neutral |
|  | T257N | 0.16 | 0.003 | -0.447 | Neutral | 1.355 | low | 0.205 | 0.051 | Neutral |
|  | V259A | 0 | 0.934 | -1.086 | Neutral | 2.535 | medium | 0.594 | 0.071 | Unknown |
|  | V259M | 0 | 0.98 | -1.203 | Neutral | 2.535 | medium | 0.517 | 0.077 | Unknown |
|  | I261V | 0.69 | 0.011 | -0.146 | Neutral | 0.25 | neutral | 0.09 | 0.031 | Neutral |
|  | I261L | 0.01 | 0.248 | -0.897 | Neutral | 2.095 | medium | 0.183 | 0.048 | Neutral |
|  | V263L | 0 | 0.978 | -1.298 | Neutral | 2.595 | medium | 0.364 | 0.056 | Unknown |
|  | V263M | 0 | 0.994 | -1.298 | Neutral | 2.595 | medium | 0.662 | 0.056 | Unknown |
|  | P266L | 0.01 | 0.998 | -4.534 | Deleterious | 2.645 | medium | 0.268 | 0.077 | Unknown |
|  | A267S | 0.47 | 0.207 | -0.289 | Neutral | 1.74 | low | 0.071 | 0.027 | Neutral |
|  | Q268R | 0.08 | 0 | -1.699 | Neutral | 2.065 | medium | 0.279 | 0.05 | Unknown |
|  | Q268H | 0.07 | 0.346 | -1.839 | Neutral | 2.615 | medium | 0.207 | 0.044 | Neutral |
|  | V270L | 0.09 | 0.62 | -0.875 | Neutral | 2.555 | medium | 0.288 | 0.078 | Unknown |
|  | W271C | 0 | 1 | -7.809 | Deleterious | 2.67 | medium | 0.784 | 0.052 | Pathogenic |
|  | W271R | 0 | 1 | -8.71 | Deleterious | 2.67 | medium | 0.813 | 0.051 | Pathogenic |
|  | R273G | 0.01 | 0.549 | -2.592 | Deleterious | 2.43 | medium | 0.494 | 0.074 | Unknown |
|  | A274V | 0.61 | 0.03 | -0.716 | Neutral | 1.68 | low | 0.27 | 0.104 | Unknown |
|  | R276Q | 0.4 | 0.091 | -1.125 | Neutral | 0.425 | neutral | 0.128 | 0.033 | Neutral |
|  | R276W | 0.07 | 0.018 | -2.24 | Neutral | -0.76 | neutral | 0.129 | 0.033 | Neutral |
|  | P277S | 0.01 | 0.104 | -2.526 | Deleterious | 2.585 | medium | 0.359 | 0.06 | Unknown |
|  | P277L | 1 | 0.315 | -1.78 | Neutral | 2.24 | medium | 0.454 | 0.113 | Unknown |
|  | G278V | 0 | 0.979 | -5.208 | Deleterious | 2.515 | medium | 0.675 | 0.073 | Unknown |
|  | G278S | 0.08 | 0.401 | -3.114 | Deleterious | 1.82 | low | 0.332 | 0.073 | Unknown |
|  | T279I | 0.04 | 0.138 | -1.48 | Neutral | 0 | neutral | 0.102 | 0.039 | Neutral |
|  | T279A | 0.26 | 0.003 | -0.151 | Neutral | 0 | neutral | 0.072 | 0.029 | Neutral |
|  | Y281H | 0.23 | 1 | -2.839 | Deleterious | 2.645 | medium | 0.612 | 0.061 | Unknown |
|  | Y281N | 0 | 0.999 | -4.919 | Deleterious | 2.645 | medium | 0.703 | 0.061 | Unknown |
|  | V282A | 0.09 | 0.624 | -1.747 | Neutral | 2.585 | medium | 0.344 | 0.067 | Unknown |
|  | V282M | 0 | 0.805 | -1.322 | Neutral | 2.585 | medium | 0.446 | 0.072 | Unknown |
|  | L286P | 0 | 0.994 | -4.295 | Deleterious | 2.595 | medium | 0.816 | 0.084 | Unknown |
|  | V288E | 0 | 0.962 | -3.581 | Deleterious | 2.34 | medium | 0.475 | 0.077 | Unknown |
|  | V288L | 0.03 | 0.596 | -0.972 | Neutral | 2.34 | medium | 0.181 | 0.07 | Neutral |
|  | V288M | 0.13 | 0.972 | -1.144 | Neutral | 2.34 | medium | 0.372 | 0.068 | Unknown |
|  | I291M | 0.01 | 0.817 | -0.81 | Neutral | 2.3 | medium | 0.256 | 0.06 | Unknown |
|  | R292H | 0.17 | 0.062 | -1.464 | Neutral | 0.66 | neutral | 0.081 | 0.037 | Neutral |
|  | R292C | 0.03 | 0.099 | -3.704 | Deleterious | 1.355 | low | 0.245 | 0.071 | Unknown |
|  | G293A | 0.1 | 0.607 | -2.263 | Neutral | 2.615 | medium | 0.199 | 0.043 | Neutral |
|  | R295H | 0.25 | 0.02 | -1.36 | Neutral | 1.555 | low | 0.232 | 0.063 | Unknown |
|  | R295C | 0.11 | 0.03 | -2.489 | Neutral | 1.555 | low | 0.12 | 0.043 | Neutral |
|  | Q296H | 0.59 | 0 | 0.776 | Neutral | -0.205 | neutral | 0.11 | 0.04 | Neutral |
|  | Q296L | 0.58 | 0.043 | -1.571 | Neutral | 0.345 | neutral | 0.088 | 0.026 | Neutral |
|  | Q296R | 0.36 | 0.005 | -0.507 | Neutral | 0.345 | neutral | 0.26 | 0.055 | Unknown |
|  | H297R | 1 | 0 | 1.007 | Neutral | -1.475 | neutral | 0.053 | 0.023 | Neutral |
|  | S302T | 0.33 | 0.013 | -0.542 | Neutral | 1.255 | low | 0.13 | 0.036 | Neutral |
|  | S302G | 0.14 | 0.017 | -2.034 | Neutral | 1.95 | medium | 0.21 | 0.055 | Neutral |
|  | Q303R | 0.05 | 0.173 | -1.02 | Neutral | 0 | neutral | 0.323 | 0.063 | Unknown |
|  | S304Y | 0 | 0.979 | -3.602 | Deleterious | 2.535 | medium | 0.569 | 0.064 | Unknown |
|  | S304A | 0.06 | 0.113 | -1.478 | Neutral | 1.845 | low | 0.151 | 0.047 | Neutral |
|  | S305L | 0.68 | 0.158 | -3.706 | Deleterious |  |  | 0.844 | 0.041 | Pathogenic |
|  | S305W | 0.2 | 0.911 | -4.733 | Deleterious | 2.67 | medium | 0.792 | 0.044 | Pathogenic |
|  | R306H | 0.2 | 0.003 | -0.226 | Neutral | -0.6 | neutral | 0.023 | 0.012 | Neutral |
|  | R306C | 0.06 | 0.007 | -0.055 | Neutral | 0.55 | neutral | 0.056 | 0.02 | Neutral |
|  | L309P | 0.04 | 0.03 | 0.942 | Neutral | -0.65 | neutral | 0.229 | 0.05 | Neutral |
|  | P312S | 0.07 | 0.857 | -2.402 | Neutral | 2.24 | medium | 0.206 | 0.056 | Neutral |
|  | E313A | 0.05 | 0.557 | -1.677 | Neutral | 2.47 | medium | 0.406 | 0.059 | Unknown |
|  | E313K | 0.06 | 0.772 | -1.651 | Neutral | 2.47 | medium | 0.507 | 0.077 | Unknown |
|  | C314R | 0.11 | 0.059 | -2.929 | Deleterious | 1.61 | low | 0.318 | 0.063 | Unknown |
|  | V315M | 0.04 | 0.984 | -1.651 | Neutral | 2.505 | medium | 0.281 | 0.055 | Unknown |
|  | Q316R | 0.72 | 0.007 | -1.08 | Neutral | -0.775 | neutral | 0.123 | 0.035 | Neutral |
|  | E317Q | 0.19 | 0.931 | -0.641 | Neutral | 2.565 | medium | 0.344 | 0.061 | Unknown |
|  | L320F | 0.01 | 0.571 | -1.131 | Neutral |  |  | 0.23 | 0.067 | Unknown |
|  | L327S | 0.08 | 0.272 | -0.408 | Neutral | 1.495 | low | 0.071 | 0.03 | Neutral |
|  | A329T | 0.28 | 0.073 | -1.355 | Neutral | 2.155 | medium | 0.012 | 0.008 | Neutral |
|  | P331R | 0.06 | 0.067 | -2.325 | Neutral | 2.47 | medium | 0.127 | 0.045 | Neutral |
|  | P331T | 0.06 | 0.124 | -2.224 | Neutral | 2.47 | medium | 0.111 | 0.057 | Neutral |
|  | K332R | 0.1 | 0.011 | -1.1 | Neutral | 2.075 | medium | 0.065 | 0.022 | Neutral |
|  | P335S | 0.15 | 0.426 | -1.288 | Neutral | 2.505 | medium | 0.081 | 0.041 | Neutral |
|  | M336T | 0.54 | 0.001 | -0.313 | Neutral | -0.145 | neutral | 0.034 | 0.016 | Neutral |
|  | N339S | 0.41 | 0.02 | -0.734 | Neutral | 0.11 | neutral | 0.08 | 0.027 | Neutral |
|  | S340L | 0.18 | 0.029 | -2.406 | Neutral | 2.175 | medium | 0.104 | 0.033 | Neutral |
|  | S340W | 0.01 | 0.928 | -2.974 | Deleterious | 2.175 | medium | 0.263 | 0.074 | Unknown |
|  | E341G | 0.03 | 0.199 | -1.634 | Neutral | 0.345 | neutral | 0.144 | 0.047 | Neutral |
|  | E341K | 0.2 | 0.007 | -0.538 | Neutral | -0.205 | neutral | 0.1 | 0.039 | Neutral |
|  | D342E | 0.07 | 0.068 | -0.534 | Neutral | 2.535 | medium | 0.062 | 0.03 | Neutral |
|  | D342G | 0.01 | 0.059 | -2.003 | Neutral | 2.535 | medium | 0.103 | 0.034 | Neutral |
|  | K343R | 0.79 | 0.001 | -0.263 | Neutral | 0.365 | neutral | 0.062 | 0.022 | Neutral |
|  | K343Q | 0.29 | 0.015 | -0.187 | Neutral | 1.61 | low | 0.183 | 0.051 | Neutral |
|  | K344T | 0.03 | 0.049 | -1.181 | Neutral | 1.05 | low | 0.185 | 0.043 | Neutral |
|  | S348R | 0.05 | 0.005 | -1.253 | Neutral | 0 | neutral | 0.212 | 0.063 | Neutral |
|  | S348N | 0.1 | 0.003 | -0.357 | Neutral | -0.55 | neutral | 0.118 | 0.038 | Neutral |
|  | S348G | 1 | 0 | 1.327 | Neutral | -2.32 | neutral | 0.045 | 0.022 | Neutral |
|  | L349F | 0.03 | 0.132 | -1.534 | Neutral | 2.52 | medium | 0.192 | 0.071 | Unknown |
|  | L349V | 0.15 | 0.139 | -0.865 | Neutral | 2.175 | medium | 0.106 | 0.04 | Neutral |
|  | R351H | 0 | 0.985 | -2.061 | Neutral |  |  | 0.438 | 0.071 | Unknown |
|  | R351C | 0 | 0.996 | -3.14 | Deleterious |  |  | 0.766 | 0.055 | Pathogenic |
|  | R351Q | 0.1 | 0.049 | -1.011 | Neutral | 1.78 | low | 0.139 | 0.041 | Neutral |
|  | R351W | 0 | 0.917 | -2.831 | Deleterious | 2.47 | medium | 0.7 | 0.072 | Unknown |
|  | S353C | 0 | 0.967 | -2.489 | Neutral | 2.555 | medium | 0.351 | 0.078 | Unknown |
|  | S353Y | 0 | 0.967 | -3.135 | Deleterious | 2.555 | medium | 0.525 | 0.072 | Unknown |
|  | R354H | 0.61 | 0 | -1.846 | Neutral |  |  | 0.196 | 0.053 | Neutral |
|  | R354C | 0.18 | 0.436 | -2.372 | Neutral |  |  | 0.429 | 0.09 | Unknown |
|  | L355F | 0.01 | 0.571 | -1.735 | Neutral | 2.4 | medium | 0.217 | 0.089 | Unknown |
|  | L356V | 0.01 | 0.994 | -0.794 | Neutral | 2.615 | medium | 0.199 | 0.047 | Neutral |
|  | S359R | 0.1 | 0 | -0.084 | Neutral |  |  | 0.233 | 0.07 | Unknown |
|  | S359L | 0.35 | 0 | -0.67 | Neutral | 0.14 | neutral | 0.233 | 0.059 | Neutral |
|  | G360R | 0 | 0.999 | -6.307 | Deleterious | 2.67 | medium | 0.875 | 0.035 | Pathogenic |
|  | A361S | 0.04 | 0.015 | -0.054 | Neutral | -0.345 | neutral | 0.27 | 0.086 | Unknown |
|  | V362G | 0 | 0.982 | -5.423 | Deleterious | 2.645 | medium | 0.167 | 0.093 | Unknown |
|  | V362D | 0 | 0.987 | -5.328 | Deleterious | 2.645 | medium | 0.694 | 0.075 | Unknown |
|  | V362L | 0.01 | 0.726 | -1.889 | Neutral | 2.3 | medium | 0.211 | 0.053 | Neutral |
|  | T363N | 0 | 0.998 | -3.704 | Deleterious | 2.67 | medium | 0.704 | 0.064 | Unknown |
|  | V365A | 0 | 0.269 | -2.992 | Deleterious | 2.565 | medium | 0.453 | 0.072 | Unknown |
|  | V365M | 0 | 0.188 | -1.956 | Neutral | 2.565 | medium | 0.635 | 0.063 | Unknown |
|  | A370T | 0 | 0.998 | -2.772 | Deleterious | 2.67 | medium | 0.714 | 0.095 | Unknown |
|  | G371S | 0.08 | 0.334 | -4.333 | Deleterious | 1.895 | low | 0.556 | 0.09 | Unknown |
|  | Y373H | 0 | 0.931 | -3.894 | Deleterious | 2.67 | medium | 0.771 | 0.059 | Pathogenic |
|  | E374G | 0.01 | 0.892 | -4.36 | Deleterious | 2.555 | medium | 0.456 | 0.113 | Unknown |
|  | G380R | 0.09 | 0.007 | -1.129 | Neutral | 2.015 | medium | 0.187 | 0.062 | Neutral |
|  | L381I | 0 | 0.994 | -1.529 | Neutral | 2.67 | medium | 0.33 | 0.066 | Unknown |
|  | L383F | 0.02 | 0.092 | -2.169 | Neutral | 1.875 | low | 0.203 | 0.046 | Neutral |
|  | A384V | 0 | 0.846 | -2.582 | Deleterious | 2.22 | medium | 0.75 | 0.063 | Unknown |
|  | Y385S | 0 | 0.998 | -6.143 | Deleterious | 2.67 | medium | 0.687 | 0.061 | Unknown |
|  | R389H | 0.61 | 0 | 0.731 | Neutral | -1.14 | neutral | 0.101 | 0.039 | Neutral |
|  | R389C | 0.18 | 0.436 | -1.437 | Neutral | 1.1 | low | 0.522 | 0.076 | Unknown |
|  | R393P | 0 | 0.626 | -4.607 | Deleterious | 2.515 | medium | 0.672 | 0.089 | Unknown |
|  | R393Q | 0.06 | 0.013 | -2.719 | Deleterious | 1.82 | low | 0.134 | 0.043 | Neutral |
|  | R393W | 0 | 0.742 | -5.358 | Deleterious | 2.515 | medium | 0.659 | 0.063 | Unknown |
|  | V394F | 0 | 0.848 | -1.704 | Neutral |  |  | 0.55 | 0.069 | Unknown |
|  | V394I | 0.16 | 0.019 | -0.788 | Neutral |  |  | 0.163 | 0.039 | Neutral |
|  | M395I | 0.47 | 0 | -0.581 | Neutral | 0.225 | neutral | 0.11 | 0.038 | Neutral |
|  | M395T | 0 | 0.015 | -3.021 | Deleterious | 1.61 | low | 0.363 | 0.068 | Unknown |
|  | R396Q | 0 | 0.996 | -3.153 | Deleterious | 2.67 | medium | 0.694 | 0.069 | Unknown |
|  | G398A | - | 0 | -4.587 | Deleterious | 2.67 | medium | 0.627 | 0.076 | Unknown |
|  | G398E | - | 0 | -6.14 | Deleterious | 2.67 | medium | 0.869 | 0.04 | Pathogenic |
|  | V399A | 0.67 | 0.006 | 0.443 | Neutral | 1.32 | low | 0.186 | 0.046 | Neutral |
|  | V399M | 0.01 | 0.499 | -1.216 | Neutral | 2.47 | medium | 0.48 | 0.072 | Unknown |
|  | C400Y | 0.14 | 0.081 | -4.477 | Deleterious | 1.845 | low | 0.273 | 0.066 | Unknown |
|  | L403F | 0 | 0.998 | -2.63 | Deleterious | 2.615 | medium | 0.26 | 0.066 | Unknown |
|  | Q404R | 0.57 | 0.003 | -0.707 | Neutral | 1.095 | low | 0.169 | 0.043 | Neutral |
|  | D405G | 0 | 0.998 | -3.757 | Deleterious | 2.645 | medium | 0.33 | 0.069 | Unknown |
|  | V406A | 0.15 | 0.994 | -1.153 | Neutral | 2.3 | medium | 0.338 | 0.061 | Unknown |
|  | H407L | 0.28 | 0 | -7.862 | Deleterious | 2.67 | medium | 0.765 | 0.046 | Pathogenic |
|  | H407R | 0.11 | 0 | -5.688 | Deleterious | 2.67 | medium | 0.777 | 0.082 | Unknown |
|  | H407N | 0 | 0.993 | -5.138 | Deleterious | 2.67 | medium | 0.684 | 0.077 | Unknown |
|  | L408P | 0 | 0.976 | -4.328 | Deleterious | 2.585 | medium | 0.899 | 0.03 | Pathogenic |
|  | S411T | 0.08 | 0.346 | -0.974 | Neutral | 2.47 | medium | 0.095 | 0.031 | Neutral |
|  | G414R | 0.01 | 0.138 | -2.331 | Neutral | 2.585 | medium | 0.437 | 0.071 | Unknown |
|  | G414A | 0.8 | 0.999 | -0.849 | Neutral | 2.585 | medium | 0.191 | 0.044 | Neutral |
|  | G414E | 0.15 | 1 | -1.64 | Neutral | 2.585 | medium | 0.408 | 0.089 | Unknown |
|  | G414W | 0.01 | 1 | -2.997 | Deleterious | 2.585 | medium | 0.463 | 0.063 | Unknown |
|  | R418K | 0.35 | 0.01 | -0.563 | Neutral | 1.705 | low | 0.115 | 0.038 | Neutral |
|  | V420A | 0.03 | 0.164 | -2.249 | Neutral | 2.535 | medium | 0.287 | 0.057 | Unknown |
|  | A422V | 0.04 | 0.998 | -2.034 | Neutral | 2.585 | medium | 0.185 | 0.046 | Neutral |
|  | A422S | 0.04 | 0.998 | -1.079 | Neutral | 2.585 | medium | 0.156 | 0.062 | Neutral |
|  | A422T | 0.01 | 0.998 | -1.701 | Neutral | 2.24 | medium | 0.408 | 0.099 | Unknown |
|  | P423A | 1 | 0.003 | -0.061 | Neutral | 1.175 | low | 0.082 | 0.028 | Neutral |
|  | C424G | 0 | 0.997 | -8.841 | Deleterious | 2.67 | medium | 0.33 | 0.064 | Unknown |
|  | C424S | 0.02 | 0.996 | -7.407 | Deleterious | 2.67 | medium | 0.213 | 0.054 | Neutral |
|  | L425P | 0 | 0.943 | -3.955 | Deleterious | 2.595 | medium | 0.83 | 0.067 | Pathogenic |
|  | R426H | 0.61 | 0.005 | -1.204 | Neutral | 0.835 | low | 0.118 | 0.047 | Neutral |
|  | R426C | 0.01 | 0.742 | -3.597 | Deleterious | 2.28 | medium | 0.288 | 0.065 | Unknown |
|  | G427V | 0.01 | 0.961 | -4 | Deleterious | 2.585 | medium | 0.334 | 0.074 | Unknown |
|  | G427S | 0.78 | 0.59 | -0.54 | Neutral | 1.895 | low | 0.179 | 0.047 | Neutral |
|  | A428T | 0.95 | 0.077 | -0.177 | Neutral | 1.47 | low | 0.233 | 0.093 | Unknown |
|  | V429F | 0 | 0.848 | -3.208 | Deleterious | 2.565 | medium | 0.507 | 0.081 | Unknown |
|  | V429I | 0.17 | 0.019 | -0.388 | Neutral | 1.875 | low | 0.26 | 0.071 | Unknown |
|  | L430V | 0.47 | 0.423 | -0.959 | Neutral | 2.47 | medium | 0.109 | 0.042 | Neutral |
|  | L431V | 0.1 | 0.03 | -0.727 | Neutral | 1.64 | low | 0.123 | 0.038 | Neutral |
|  | S433G | 1 | 0 | -1.238 | Neutral | -1.02 | neutral | 0.057 | 0.024 | Neutral |
|  | F434L | 0 | 0.613 | -4.206 | Deleterious | 2.67 | medium | 0.344 | 0.074 | Unknown |
|  | R436L | 0.15 | 0.147 | -2.35 | Neutral | 1.59 | low | 0.188 | 0.078 | Unknown |
|  | R436P | 0.13 | 0.706 | -2.437 | Neutral | 1.59 | low | 0.297 | 0.098 | Unknown |
|  | R436H | 0.15 | 0.007 | -1.925 | Neutral | 0.895 | low | 0.166 | 0.052 | Neutral |
|  | R436C | 0.08 | 0.001 | -0.921 | Neutral | -0.535 | neutral | 0.034 | 0.014 | Neutral |
|  | K438N | 0.5 | 0.037 | -0.999 | Neutral | 2.34 | medium | 0.204 | 0.058 | Neutral |
|  | P439S | 0.1 | 0.177 | -0.688 | Neutral | 2.005 | medium | 0.129 | 0.047 | Neutral |
|  | G440A | 0.72 | 0.025 | -0.132 | Neutral | 1.39 | low | 0.064 | 0.024 | Neutral |
|  | A441V | 0.11 | 0.006 | -0.186 | Neutral | 0.895 | low | 0.077 | 0.024 | Neutral |
|  | H442L | 0.3 | 0 | -1.651 | Neutral | -0.345 | neutral | 0.209 | 0.052 | Neutral |
|  | H442R | 0.11 | 0 | -1.251 | Neutral | 0.345 | neutral | 0.267 | 0.066 | Unknown |
|  | S444T | 0.25 | 0.295 | -0.384 | Neutral | 2.47 | medium | 0.2 | 0.053 | Neutral |
|  | R445L | 0.55 | 0 | -0.509 | Neutral | -0.69 | neutral | 0.096 | 0.034 | Neutral |
|  | R445H | 1 | 0 | 1.738 | Neutral | -2.3 | neutral | 0.042 | 0.018 | Neutral |
|  | R445C | 0.12 | 0.365 | -0.535 | Neutral | -0.345 | neutral | 0.48 | 0.085 | Unknown |
|  | A447T | 0.5 | 0.124 | -0.721 | Neutral | 2.25 | medium | 0.255 | 0.074 | Unknown |
|  | Y448C | 0.2 | 0 | -1.314 | Neutral | 0.345 | neutral | 0.068 | 0.029 | Neutral |
|  | G449R | 0.01 | 0.138 | -2.523 | Deleterious | 2.535 | medium | 0.236 | 0.058 | Neutral |
|  | A450V | 0.05 | 0.218 | -1.439 | Neutral | 2.175 | medium | 0.097 | 0.042 | Neutral |
|  | A450D | 0.01 | 0.967 | -1.659 | Neutral | 2.52 | medium | 0.516 | 0.084 | Unknown |
|  | A450P | 0.02 | 0.967 | -0.989 | Neutral | 2.52 | medium | 0.445 | 0.088 | Unknown |
|  | S451C | 0.02 | 0.799 | -1.323 | Neutral | 2.34 | medium | 0.304 | 0.131 | Unknown |
|  | S451A | 0.09 | 0.124 | -0.841 | Neutral | 2.34 | medium | 0.134 | 0.042 | Neutral |
|  | Y453F | 0.06 | 0.018 | -2.157 | Neutral | 2.555 | medium | 0.119 | 0.039 | Neutral |
|  | E454K | 0.09 | 0.381 | -0.558 | Neutral | 2.43 | medium | 0.22 | 0.063 | Unknown |
|  | V457L | 1 | 0.011 | 0.249 | Neutral | 1.445 | low | 0.12 | 0.038 | Neutral |
|  | R460P | 0.11 | 0.619 | -2.328 | Neutral | 1.845 | low | 0.478 | 0.102 | Unknown |
|  | R460H | 0.44 | 0.003 | -0.213 | Neutral | 0.235 | neutral | 0.063 | 0.028 | Neutral |
|  | R460C | 0.13 | 0.007 | -2.037 | Neutral | 1.295 | low | 0.204 | 0.059 | Neutral |
|  | Q461H | 0.12 | 0.009 | 0.099 | Neutral | 1.285 | low | 0.186 | 0.037 | Neutral |
|  | Q461P | 0.02 | 0.731 | -2.098 | Neutral | 2.43 | medium | 0.602 | 0.06 | Unknown |
|  | Q461K | 0.04 | 0.297 | -1.144 | Neutral | 2.43 | medium | 0.313 | 0.065 | Unknown |
|  | P465S | 0.06 | 0.011 | -3.64 | Deleterious | 2.645 | medium | 0.191 | 0.055 | Neutral |
|  | P465L | 0 | 0.998 | -5.026 | Deleterious | 2.645 | medium | 0.53 | 0.074 | Unknown |
|  | L466V | 0.09 | 0.03 | -1.035 | Neutral | 1.755 | low | 0.106 | 0.031 | Neutral |
|  | T471I | 0.05 | 0.054 | -0.096 | Neutral | 2.075 | medium | 0.099 | 0.055 | Neutral |
|  | T471S | 0.34 | 0.005 | -1.054 | Neutral | 0.975 | low | 0.034 | 0.016 | Neutral |
|  | A473T | 0.22 | 0.018 | -0.606 | Neutral | 1.395 | low | 0.135 | 0.048 | Neutral |
|  | L474P | 0 | 0.999 | -2.923 | Deleterious | 2.615 | medium | 0.747 | 0.106 | Unknown |
|  | C479F | 0.7 | 0.005 | -1.613 | Neutral | 2.2 | medium | 0.195 | 0.055 | Neutral |
|  | K480R | 0.66 | 0.994 | -0.556 | Neutral | 2.615 | medium | 0.102 | 0.036 | Neutral |
|  | P483R | 0.02 | 0.998 | -3.619 | Deleterious | 2.645 | medium | 0.229 | 0.048 | Neutral |
|  | P483A | 0.17 | 0.996 | -3.402 | Deleterious | 2.645 | medium | 0.123 | 0.035 | Neutral |
|  | H484P | 0.15 | 0.998 | -2.735 | Deleterious | 2.555 | medium | 0.62 | 0.068 | Unknown |
|  | H484R | 0.15 | 0.059 | -1.622 | Neutral | 2.005 | medium | 0.325 | 0.065 | Unknown |
|  | V485A | 0.01 | 0.115 | -1.461 | Neutral | 2.125 | medium | 0.146 | 0.04 | Neutral |
|  | V485L | 0.39 | 0.006 | 0.21 | Neutral | 0.97 | low | 0.119 | 0.038 | Neutral |
|  | R487W | 0.01 | 0.999 | -3.354 | Deleterious |  |  | 0.466 | 0.064 | Unknown |
|  | R487G | 0 | 0.998 | -1.852 | Neutral | 2.555 | medium | 0.563 | 0.062 | Unknown |
|  | H488R | 0.31 | 0.367 | -2.009 | Neutral | 2.47 | medium | 0.323 | 0.056 | Unknown |
|  | H489D | 0.02 | 0.081 | -2.725 | Deleterious | 2.28 | medium | 0.303 | 0.055 | Unknown |
|  | F491C | 0 | 0.785 | -2.288 | Neutral | 2.085 | medium | 0.717 | 0.062 | Unknown |
|  | F491V | 0.04 | 0.115 | -1.776 | Neutral | 2.43 | medium | 0.499 | 0.067 | Unknown |
|  | Q493R | 0.65 | 0.165 | -0.696 | Neutral | 2.35 | medium | 0.283 | 0.078 | Unknown |
|  | H494Y | 0.93 | 0 | -1.444 | Neutral | 1.375 | low | 0.193 | 0.084 | Unknown |
|  | S496F | 0.02 | 0.005 | -2.384 | Neutral | 2.24 | medium | 0.134 | 0.042 | Neutral |
|  | P497A | 0.16 | 0.71 | -1.95 | Neutral | 2.43 | medium | 0.102 | 0.032 | Neutral |
|  | S499I | 0.05 | 0.102 | -2.128 | Neutral | 1.1 | low | 0.253 | 0.07 | Unknown |
|  | S499T | 0.28 | 0.001 | -0.42 | Neutral | 0.55 | neutral | 0.045 | 0.018 | Neutral |
|  | P500S | 0.05 | 0.011 | -2.331 | Neutral | 2.585 | medium | 0.178 | 0.047 | Neutral |
|  | S501I | 0.02 | 0.555 | -2.679 | Deleterious | 2.43 | medium | 0.447 | 0.072 | Unknown |
|  | S501N | 0.26 | 0.007 | -0.813 | Neutral | 2.43 | medium | 0.385 | 0.079 | Unknown |
|  | L502P | 0 | 0.999 | -1.03 | Neutral | 2.24 | medium | 0.144 | 0.04 | Neutral |
|  | G503R | 0 | 0.999 | -2.661 | Deleterious | 2.555 | medium | 0.494 | 0.07 | Unknown |
|  | A508T | 0.21 | 0.018 | -0.755 | Neutral | 1.52 | low | 0.171 | 0.057 | Neutral |
|  | P509S | 0.12 | 0.998 | -3.164 | Deleterious | 2.615 | medium | 0.435 | 0.083 | Unknown |
|  | T510I | 0.64 | 0.003 | 0.029 | Neutral | 1.43 | low | 0.091 | 0.037 | Neutral |
|  | T510A | 0.51 | 0.003 | -0.841 | Neutral | 1.025 | low | 0.093 | 0.037 | Neutral |
|  | D512V | 0.01 | 0.732 | -3.286 | Deleterious | 2.32 | medium | 0.207 | 0.057 | Neutral |
|  | L514P | 0 | 0.999 | -1.995 | Neutral | 2.555 | medium | 0.707 | 0.077 | Unknown |
|  | P516L | 0.11 | 0.062 | -4.472 | Deleterious | 1.81 | low | 0.383 | 0.081 | Unknown |
|  | G518D | 0.63 | 0.007 | -1.59 | Neutral | 1.2 | low | 0.261 | 0.064 | Unknown |
|  | P520A | 0.22 | 0.003 | -1.216 | Neutral | 1.43 | low | 0.085 | 0.027 | Neutral |
|  | V521F | 0.18 | 0 | -1.295 | Neutral | 0 | neutral | 0.154 | 0.045 | Neutral |
|  | R522Q | 0 | 0.996 | -2.463 | Neutral | 2.67 | medium | 0.685 | 0.096 | Unknown |
|  | R522W | 0.01 | 0.999 | -5.307 | Deleterious | 2.67 | medium | 0.829 | 0.049 | Pathogenic |
|  | R522G | 0 | 0.998 | -4.257 | Deleterious | 2.67 | medium | 0.761 | 0.058 | Pathogenic |
|  | A524V | 1 | 0 | 1.037 | Neutral | 0.64 | neutral | 0.107 | 0.052 | Neutral |
|  | H525R | 0.02 | 0.118 | -3.011 | Deleterious | 2.3 | medium | 0.481 | 0.062 | Unknown |
|  | N526K | 0.84 | 0 | -0.985 | Neutral | 0 | neutral | 0.052 | 0.02 | Neutral |
|  | N526S | 0.95 | 0 | -0.562 | Neutral | -0.805 | neutral | 0.069 | 0.03 | Neutral |
|  | L529F | 0.03 | 0.099 | -1.928 | Neutral | 1.875 | low | 0.135 | 0.046 | Neutral |
|  | L529I | 0.01 | 0.348 | -0.678 | Neutral | 2.565 | medium | 0.164 | 0.04 | Neutral |
|  | E531Q | 0.01 | 0.848 | -1.279 | Neutral | 2.585 | medium | 0.283 | 0.079 | Unknown |
|  | E531K | 0.28 | 0.641 | -1.404 | Neutral | 2.24 | medium | 0.299 | 0.086 | Unknown |
|  | P532A | 0.14 | 0.71 | -3.57 | Deleterious | 2.565 | medium | 0.29 | 0.07 | Unknown |
|  | H533R | 0 | 0.996 | -5.189 | Deleterious | 2.67 | medium | 0.753 | 0.098 | Unknown |
|  | H533Y | 0 | 0.996 | -4.178 | Deleterious | 2.67 | medium | 0.492 | 0.074 | Unknown |
|  | H534Q | 0.4 | 0.01 | -2.465 | Neutral | 2.34 | medium | 0.326 | 0.055 | Unknown |
|  | C535Y | 0 | 0.998 | -7.516 | Deleterious | 2.67 | medium | 0.887 | 0.037 | Pathogenic |
|  | P536S | 0 | 0.998 | -4.189 | Deleterious | 2.645 | medium | 0.747 | 0.06 | Unknown |
|  | L537P | 0 | 0.999 | -4.028 | Deleterious | 2.645 | medium | 0.918 | 0.027 | Pathogenic |
|  | Q538R | 0.12 | 0.054 | -1.357 | Neutral | 2.035 | medium | 0.181 | 0.042 | Neutral |
|  | Y540S | 0.01 | 0.872 | -4.457 | Deleterious | 2.67 | medium | 0.713 | 0.057 | Unknown |
|  | T541S | 0.69 | 0.009 | -0.323 | Neutral | 0.975 | low | 0.031 | 0.017 | Neutral |
|  | R542Q | 0.19 | 0.007 | -0.513 | Neutral | 0.845 | low | 0.095 | 0.031 | Neutral |
|  | R542W | 0.02 | 0.736 | -2.256 | Neutral | 2.2 | medium | 0.284 | 0.081 | Unknown |
|  | Q544H | 0.02 | 0.997 | -1.774 | Neutral | 2.585 | medium | 0.38 | 0.079 | Unknown |
|  | S547Y | 0.33 | 0 | 0.751 | Neutral | -1.735 | neutral | 0.081 | 0.024 | Neutral |
|  | S548N | 0.01 | 0.666 | -1.488 | Neutral |  |  | 0.308 | 0.088 | Unknown |
|  | F549S | 0.19 | 0.997 | -3.761 | Deleterious | 2.585 | medium | 0.443 | 0.077 | Unknown |
|  | F549I | 0.34 | 0.996 | -1.654 | Neutral | 2.585 | medium | 0.296 | 0.059 | Unknown |
|  | T551I | 0.01 | 0.408 | -2.987 | Deleterious | 2.505 | medium | 0.243 | 0.07 | Unknown |
|  | E558G | 0.17 | 0.714 | -2.896 | Deleterious | 2.52 | medium | 0.227 | 0.061 | Neutral |
|  | G559R | 0 | 0.105 | -2.648 | Deleterious | 2.38 | medium | 0.112 | 0.045 | Neutral |
|  | Q560R | 0.11 | 0.003 | -1.642 | Neutral | 0.855 | low | 0.074 | 0.034 | Neutral |
|  | R561P | 0.11 | 0.242 | -2.342 | Neutral | 1.5 | low | 0.266 | 0.089 | Unknown |
|  | R561H | 0.34 | 0.001 | -0.347 | Neutral | 0.345 | neutral | 0.052 | 0.026 | Neutral |
|  | R561C | 0.28 | 0 | -2.047 | Neutral | 0 | neutral | 0.036 | 0.014 | Neutral |
|  | R561S | 0.36 | 0.003 | -1.352 | Neutral | 0.4 | neutral | 0.068 | 0.027 | Neutral |
|  | W564R | 0.01 | 0.068 | -7.745 | Deleterious | 2.645 | medium | 0.587 | 0.077 | Unknown |
|  | S566C | 0.01 | 0.729 | -2.486 | Neutral | 2.35 | medium | 0.218 | 0.076 | Unknown |
|  | P569L | 0 | 0.811 | -4.28 | Deleterious | 2.27 | medium | 0.522 | 0.073 | Unknown |
|  | P569S | 0.01 | 0.74 | -3.501 | Deleterious | 2.615 | medium | 0.214 | 0.061 | Neutral |
|  | P574R | 0 | 0.964 | -3.248 | Deleterious | 2.615 | medium | 0.584 | 0.09 | Unknown |
|  | P574S | 0.5 | 0.947 | -2.262 | Neutral | 2.27 | medium | 0.137 | 0.034 | Neutral |
|  | L575F | 0.28 | 0.018 | -0.92 | Neutral | 2.125 | medium | 0.118 | 0.041 | Neutral |
|  | P576L | 0.59 | 0.479 | -2.328 | Neutral | 2.205 | medium | 0.137 | 0.043 | Neutral |
|  | A578V | 0.13 | 0.072 | -1.836 | Neutral | 1.375 | low | 0.115 | 0.065 | Neutral |
|  | S579A | 0.9 | 0.003 | -0.929 | Neutral | 0.69 | neutral | 0.076 | 0.033 | Neutral |
|  | P582R | 0.09 | 0.299 | -1.095 | Neutral | 1.78 | low | 0.151 | 0.043 | Neutral |
|  | P582S | 0.81 | 0.005 | 0.251 | Neutral | 0.68 | neutral | 0.05 | 0.02 | Neutral |
|  | S583N | 0.01 | 0.666 | -1.717 | Neutral | 2.505 | medium | 0.397 | 0.096 | Unknown |
|  | L586F | 0 | 0.998 | -1.872 | Neutral | 2.585 | medium | 0.308 | 0.064 | Unknown |
|  | L586V | 0.02 | 0.994 | -1.178 | Neutral | 2.24 | medium | 0.373 | 0.066 | Unknown |
|  | N587S | 0 | 0.862 | -3.069 | Deleterious | 2.645 | medium | 0.716 | 0.061 | Unknown |
|  | N587H | 0 | 0.967 | -3.402 | Deleterious | 2.645 | medium | 0.581 | 0.119 | Unknown |
|  | R588H | 0.39 | 0 | -1.488 | Neutral | -0.49 | neutral | 0.014 | 0.008 | Neutral |
|  | R588C | 0.11 | 0.001 | -2.637 | Deleterious | 0.895 | low | 0.048 | 0.021 | Neutral |
|  | R588G | 0.03 | 0.029 | -1.86 | Neutral | 0.895 | low | 0.28 | 0.076 | Unknown |
|  | R589Q | 0.55 | 0.071 | -1.447 | Neutral |  |  | 0.301 | 0.08 | Unknown |
|  | R589W | 0 | 0.112 | -3.735 | Deleterious |  |  | 0.518 | 0.066 | Unknown |
|  | R589L | 0 | 0.437 | -3.164 | Deleterious | 2.555 | medium | 0.531 | 0.115 | Unknown |
|  | R589H | 0.12 | 0.015 | -1.879 | Neutral | 2.005 | medium | 0.343 | 0.061 | Unknown |
|  | R589C | 0 | 0.037 | -3.395 | Deleterious | 2.555 | medium | 0.58 | 0.097 | Unknown |
|  | A591V | 0 | 0.846 | -2.238 | Neutral | 2.505 | medium | 0.17 | 0.055 | Neutral |
|  | S593F | 0 | 0.998 | -3.625 | Deleterious | 2.645 | medium | 0.742 | 0.058 | Unknown |
|  | W594L | 0.01 | 0.998 | -5.474 | Deleterious | 2.585 | medium | 0.488 | 0.057 | Unknown |
|  | C596S | 0.51 | 0.003 | -1.895 | Neutral | 1.08 | low | 0.102 | 0.035 | Neutral |
|  | L598V | 0.84 | 0.043 | -0.786 | Neutral | 2.3 | medium | 0.131 | 0.039 | Neutral |
|  | P599S | 0.28 | 0.104 | -2.165 | Neutral | 1.68 | low | 0.173 | 0.045 | Neutral |
|  | A601T | 0.54 | 0.003 | -0.789 | Neutral | 1.1 | low | 0.076 | 0.029 | Neutral |
|  | Q606H | 0.25 | 0.007 | -2.013 | Neutral | 2.075 | medium | 0.251 | 0.054 | Neutral |
|  | L609R | 0 | 0.884 | -4.244 | Deleterious | 2.295 | medium | 0.682 | 0.061 | Unknown |
|  | L609F | 0.01 | 0.75 | -2.493 | Neutral | 2.295 | medium | 0.205 | 0.058 | Neutral |
|  | L609V | 0.19 | 0.018 | -1.229 | Neutral | 0.905 | low | 0.166 | 0.055 | Neutral |
|  | L612Q | 0 | 0.943 | -4.073 | Deleterious | 2.645 | medium | 0.541 | 0.08 | Unknown |
|  | V613L | 0.35 | 0.164 | -1.178 | Neutral | 2.505 | medium | 0.136 | 0.047 | Neutral |
|  | A614T | 0.07 | 0.067 | -1.844 | Neutral | 2.42 | medium | 0.187 | 0.053 | Neutral |
|  | K618E | 0.02 | 0.726 | -1.049 | Neutral | 2.52 | medium | 0.158 | 0.046 | Neutral |
|  | G619R | 0 | 0.928 | -4.858 | Deleterious | 2.535 | medium | 0.568 | 0.094 | Unknown |
|  | C620F | 0.62 | 0.003 | -2.569 | Deleterious | 2.24 | medium | 0.178 | 0.046 | Neutral |
|  | C620Y | 1 | 0.003 | -1.781 | Neutral | 0.85 | low | 0.093 | 0.04 | Neutral |
|  | R624Q | 0.53 | 0.071 | -0.82 | Neutral | 1.555 | low | 0.176 | 0.063 | Neutral |
|  | R624W | 0.04 | 0.112 | -3.008 | Deleterious | 2.455 | medium | 0.333 | 0.067 | Unknown |
|  | D625G | 0 | 0.999 | -5.499 | Deleterious | 1.875 | low | 0.254 | 0.056 | Unknown |
|  | S627G | 0 | 0.442 | -2.628 | Deleterious | 2.28 | medium | 0.175 | 0.047 | Neutral |
|  | G628V | 0 | 1 | -6.084 | Deleterious | 2.535 | medium | 0.438 | 0.075 | Unknown |
|  | S629Y | 0.06 | 0.804 | -3.032 | Deleterious | 2.485 | medium | 0.226 | 0.045 | Neutral |
|  | P631L | 0.01 | 0.027 | -5.274 | Deleterious | 2.425 | medium | 0.441 | 0.099 | Unknown |
|  | L633V | 0.8 | 0.043 | -1.722 | Neutral | 1.575 | low | 0.097 | 0.031 | Neutral |
|  | L635R | 0.02 | 0.999 | -2.359 | Neutral | 2.615 | medium | 0.585 | 0.085 | Unknown |
|  | K637E | 0.29 | 0.001 | -0.783 | Neutral | 0.345 | neutral | 0.062 | 0.025 | Neutral |
|  | L642V | 0.31 | 0.01 | -0.713 | Neutral | 0.975 | low | 0.074 | 0.029 | Neutral |
|  | S643C | 0.17 | 0.267 | -1.618 | Neutral | 0.345 | neutral | 0.183 | 0.049 | Neutral |
|  | D644E | 0.19 | 0.998 | -1.982 | Neutral | 2.585 | medium | 0.173 | 0.047 | Neutral |
|  | R646Q | 0.5 | 0.003 | -1.132 | Neutral | 0.39 | neutral | 0.118 | 0.066 | Neutral |
|  | R646W | 0.04 | 0.005 | -1.395 | Neutral | 1.085 | low | 0.218 | 0.051 | Neutral |
|  | L647Q | 0 | 0.943 | -2.686 | Deleterious | 2.455 | medium | 0.543 | 0.063 | Unknown |
|  | G649D | 0.08 | 0.999 | -4.347 | Deleterious | 2.595 | medium | 0.619 | 0.094 | Unknown |
|  | G649C | 0 | 1 | -6.514 | Deleterious | 2.595 | medium | 0.72 | 0.065 | Unknown |
|  | V652L | 0.51 | 0.011 | -1.381 | Neutral | 2.105 | medium | 0.12 | 0.033 | Neutral |
|  | R653Q | 0.79 | 0.996 | -2.014 | Neutral | 2.155 | medium | 0.121 | 0.037 | Neutral |
|  | R653W | 0.01 | 0.999 | -4.786 | Deleterious | 2.505 | medium | 0.509 | 0.13 | Unknown |
|  | R656S | 0.1 | 0.23 | -2.721 | Deleterious | 2.24 | medium | 0.136 | 0.05 | Neutral |
|  | F657L | 0.01 | 0.127 | -3.787 | Deleterious | 2.615 | medium | 0.194 | 0.046 | Neutral |
|  | Q658H | 0.01 | 0.875 | -2.966 | Deleterious | 2.535 | medium | 0.314 | 0.079 | Unknown |
|  | L659W | 0 | 0.996 | -4.292 | Deleterious | 2.645 | medium | 0.7 | 0.076 | Unknown |
|  | V661L | 0.01 | 0.155 | -1.598 | Neutral | 1.955 | medium | 0.072 | 0.024 | Neutral |
|  | V661I | 0.42 | 0.007 | -0.264 | Neutral | -0.06 | neutral | 0.072 | 0.035 | Neutral |
|  | E662K | 0 | 0.996 | -3.025 | Deleterious | 2.67 | medium | 0.727 | 0.067 | Unknown |
|  | D664E | 0.82 | 0.001 | -0.571 | Neutral | 0.205 | neutral | 0.041 | 0.017 | Neutral |
|  | D664N | 0.87 | 0 | -0.148 | Neutral | -0.98 | neutral | 0.046 | 0.02 | Neutral |
|  | V665G | 0 | 0.893 | -3.014 | Deleterious | 2.585 | medium | 0.32 | 0.071 | Unknown |
|  | V665M | 0.07 | 0.272 | -0.656 | Neutral | 2.585 | medium | 0.134 | 0.044 | Neutral |
|  | R666S | 0.02 | 0.23 | -1.763 | Neutral | 2.34 | medium | 0.27 | 0.058 | Unknown |
|  | P670L | 0 | 0.998 | -5.622 | Deleterious | 2.645 | medium | 0.701 | 0.097 | Unknown |
|  | P670S | 0.06 | 0.998 | -4.074 | Deleterious | 2.645 | medium | 0.552 | 0.095 | Unknown |
|  | S671F | 0 | 0.998 | -4.168 | Deleterious | 2.645 | medium | 0.787 | 0.043 | Pathogenic |
|  | S671C | 0 | 0.998 | -3.608 | Deleterious | 2.645 | medium | 0.729 | 0.073 | Unknown |
|  | L675M | 0.03 | 0.324 | -1.035 | Neutral | 2.27 | medium | 0.485 | 0.088 | Unknown |
|  | S676N | 0.4 | 0.015 | -0.383 | Neutral | 1.1 | low | 0.094 | 0.027 | Neutral |
|  | G679A | 0.32 | 0.124 | -1.587 | Neutral | 2.175 | medium | 0.084 | 0.029 | Neutral |
|  | G679D | 0.52 | 0.006 | -0.588 | Neutral | 0.565 | neutral | 0.118 | 0.035 | Neutral |
|  | G679R | 0.25 | 0.015 | -1.872 | Neutral | 1.825 | low | 0.15 | 0.047 | Neutral |
|  | G679S | 0.54 | 0.02 | -1.069 | Neutral | 1.825 | low | 0.245 | 0.065 | Unknown |
|  | A686T | 0.86 | 0.044 | -1.344 | Neutral | 1.74 | low | 0.113 | 0.039 | Neutral |
|  | V688I | 0.34 | 0.026 | -0.378 | Neutral | 1.32 | low | 0.324 | 0.101 | Unknown |
|  | Y689C | 0 | 0.967 | -6.067 | Deleterious | 2.67 | medium | 0.839 | 0.047 | Pathogenic |
|  | V690I | 0.09 | 0.011 | -0.257 | Neutral | 1.115 | low | 0.288 | 0.081 | Unknown |
|  | D696N | 0.02 | 0.789 | -3.489 | Deleterious | 2.565 | medium | 0.52 | 0.075 | Unknown |
|  | A697G | 0 | 0.475 | -2.797 | Deleterious | 2.535 | medium | 0.161 | 0.05 | Neutral |
|  | A697T | 0.03 | 0.675 | -2.035 | Neutral | 2.535 | medium | 0.308 | 0.086 | Unknown |
|  | L698P | 0.09 | 0.999 | -3.835 | Deleterious | 2.585 | medium | 0.795 | 0.09 | Unknown |
|  | L700P | 0 | 0.943 | -4.573 | Deleterious | 2.595 | medium | 0.837 | 0.055 | Pathogenic |
|  | P701L | 0.24 | 0.75 | -4.613 | Deleterious | 2.32 | medium | 0.544 | 0.205 | Unknown |
|  | V702L | 0.09 | 0.009 | -1.086 | Neutral | 2.34 | medium | 0.109 | 0.045 | Neutral |
|  | P703S | 0.03 | 0.906 | -2.879 | Deleterious | 2.005 | medium | 0.159 | 0.043 | Neutral |
|  | R704T | 0.04 | 0.294 | -2.41 | Neutral | 2.52 | medium | 0.13 | 0.034 | Neutral |
|  | L707F | 0.23 | 0.003 | -0.748 | Neutral | 1.53 | low | 0.103 | 0.042 | Neutral |
|  | S709L | 0.01 | 0.009 | -2.537 | Deleterious | 2.135 | medium | 0.149 | 0.052 | Neutral |
|  | A710V | 0.12 | 0.541 | -0.873 | Neutral | 1.5 | low | 0.073 | 0.028 | Neutral |
|  | A710T | 0.19 | 0.378 | -1.079 | Neutral | 2.2 | medium | 0.177 | 0.058 | Neutral |
|  | T711A | 0.2 | 0.003 | -1.489 | Neutral | 2.14 | medium | 0.056 | 0.025 | Neutral |
|  | P712S | 0.98 | 0 | -1.521 | Neutral | -1.05 | neutral | 0.038 | 0.017 | Neutral |
|  | P712T | 1 | 0.017 | -1.849 | Neutral | 0.895 | low | 0.029 | 0.014 | Neutral |
|  | S713L | 0.09 | 0.001 | -2.859 | Deleterious | 1.1 | low | 0.047 | 0.019 | Neutral |
|  | S713P | 0.03 | 0.277 | -2.027 | Neutral | 1.79 | low | 0.142 | 0.045 | Neutral |
|  | T714I | 0.19 | 0.112 | -1.785 | Neutral | 1.39 | low | 0.1 | 0.04 | Neutral |
|  | P715Q | 0.13 | 0.033 | -3.231 | Deleterious |  |  | 0.189 | 0.053 | Neutral |
|  | P715L | 0.08 | 0.22 | -3.314 | Deleterious | 2.28 | medium | 0.109 | 0.043 | Neutral |
|  | P715S | 0.08 | 0.01 | -2.777 | Deleterious | 1.245 | low | 0.052 | 0.022 | Neutral |
|  | Q716R | 0.2 | 0.003 | -2.088 | Neutral | 1.59 | low | 0.209 | 0.044 | Neutral |
|  | D718E | 1 | 0 | -0.753 | Neutral | -1.3 | neutral | 0.052 | 0.025 | Neutral |
|  | D718H | 0.01 | 0.371 | -2.731 | Deleterious | 0.895 | low | 0.291 | 0.074 | Unknown |
|  | P719A | 0.78 | 0.059 | -3.021 | Deleterious | 2.3 | medium | 0.105 | 0.04 | Neutral |
|  | P719L | 0.5 | 0.23 | -4.059 | Deleterious | 2.3 | medium | 0.445 | 0.095 | Unknown |
|  | T720I | 0.07 | 0.316 | -2.91 | Deleterious | 1.79 | low | 0.106 | 0.043 | Neutral |
|  | G721A | 0.53 | 0.01 | 0.16 | Neutral | 0.69 | neutral | 0.121 | 0.043 | Neutral |
|  | G721S | 0.45 | 0.003 | -0.187 | Neutral | 0.69 | neutral | 0.084 | 0.032 | Neutral |
|  | P722S | 0.45 | 0.055 | -1.579 | Neutral | 1.59 | low | 0.106 | 0.041 | Neutral |
|  | P722T | 0.25 | 0.224 | -2.027 | Neutral | 2.28 | medium | 0.23 | 0.054 | Neutral |
|  | E723K | 0.09 | 0.01 | -2.343 | Neutral | 2.395 | medium | 0.097 | 0.035 | Neutral |
|  | P725R | 0.23 | 0.804 | -4.003 | Deleterious | 2.075 | medium | 0.223 | 0.056 | Neutral |
|  | H726R | 0.51 | 0.055 | -1.546 | Neutral | 2.19 | medium | 0.164 | 0.04 | Neutral |
|  | G728E | 0.23 | 0.003 | -2.306 | Neutral | 0.65 | neutral | 0.065 | 0.025 | Neutral |
|  | G728R | 0.1 | 0.034 | -2.513 | Deleterious | 2.255 | medium | 0.092 | 0.034 | Neutral |
|  | Q729H | 0.03 | 0.987 | -3.27 | Deleterious | 2.615 | medium | 0.289 | 0.069 | Unknown |
|  | S730R | 0* | 1 | -3.032 | Deleterious | 2.645 | medium | 0.588 | 0.067 | Unknown |
|  | S730G | 0.02 | 0.996 | -2.987 | Deleterious | 2.645 | medium | 0.405 | 0.074 | Unknown |
|  | R731L | 0 | 0.999 | -4.144 | Deleterious | 2.615 | medium | 0.597 | 0.063 | Unknown |
|  | R731Q | 0.05 | 0.998 | -2.321 | Neutral | 2.615 | medium | 0.33 | 0.112 | Unknown |
|  | R731W | 0 | 1 | -5.379 | Deleterious | 2.615 | medium | 0.607 | 0.078 | Unknown |
|  | C736R | 0.01 | 0.035 | -1.308 | Neutral | 0 | neutral | 0.313 | 0.057 | Unknown |
|  | H737R | 0.02 | 0.022 | -3.579 | Deleterious | 1.78 | low | 0.319 | 0.063 | Unknown |
|  | M742I | 0.04 | 0.006 | -1.66 | Neutral | 1.845 | low | 0.222 | 0.052 | Neutral |
|  | M742T | 0.01 | 0.354 | -3.19 | Deleterious | 2.535 | medium | 0.517 | 0.068 | Unknown |
|  | M742V | 0.04 | 0.073 | -1.565 | Neutral | 2.19 | medium | 0.136 | 0.037 | Neutral |
|  | R744P | 0 | 0.935 | -3.692 | Deleterious | 2.19 | medium | 0.752 | 0.063 | Unknown |
|  | R744H | 0 | 0.917 | -2.556 | Deleterious | 2.535 | medium | 0.29 | 0.08 | Unknown |
|  | R744C | 0 | 0.917 | -3.879 | Deleterious | 2.535 | medium | 0.703 | 0.06 | Unknown |
|  | R744G | 0 | 0.905 | -4.216 | Deleterious | 2.535 | medium | 0.678 | 0.066 | Unknown |
|  | N745H | 0 | 0.999 | -3.175 | Deleterious | 2.615 | medium | 0.529 | 0.067 | Unknown |
|  | C747S | 0.38 | 0.003 | -2.937 | Deleterious | 1.645 | low | 0.091 | 0.029 | Neutral |
|  | V748I | 0.39 | 0.001 | -0.202 | Neutral | 1.5 | low | 0.051 | 0.021 | Neutral |
|  | P749S | 0.12 | 0.466 | -0.002 | Neutral | 0.725 | neutral | 0.063 | 0.025 | Neutral |
|  | P749A | 0.25 | 0.124 | -0.435 | Neutral | 1.735 | low | 0.084 | 0.029 | Neutral |
|  | P749T | 0.18 | 0.178 | -0.483 | Neutral | 2.08 | medium | 0.172 | 0.045 | Neutral |
|  | P750Q | 0.12 | 0.033 | -1.879 | Neutral | 1.835 | low | 0.077 | 0.032 | Neutral |
|  | A752E | 0.4 | 0.124 | -1.019 | Neutral | 2.39 | medium | 0.248 | 0.061 | Unknown |
|  | A752T | 0.14 | 0.012 | -1.512 | Neutral | 1.7 | low | 0.192 | 0.058 | Neutral |
|  | P754A | 0.78 | 0.059 | -1.117 | Neutral | 1.355 | low | 0.08 | 0.029 | Neutral |
|  | E755G | 0.02 | 0.003 | -3.264 | Deleterious | 1.35 | low | 0.164 | 0.051 | Neutral |
|  | V756M | 0.11 | 0.024 | -0.978 | Neutral | 2.08 | medium | 0.168 | 0.045 | Neutral |
|  | P757S | 0.48 | 0.055 | -1.215 | Neutral | 2.24 | medium | 0.151 | 0.052 | Neutral |
|  | P757T | 0.23 | 0.224 | -1.708 | Neutral | 2.24 | medium | 0.206 | 0.047 | Neutral |
|  | A760T | 1 | 0 | 1.275 | Neutral | -2.505 | neutral | 0.021 | 0.011 | Neutral |
|  | L761F | 0.26 | 0.043 | -0.837 | Neutral | 1.32 | low | 0.128 | 0.041 | Neutral |
|  | S762T | 0.01 | 0.636 | -2.106 | Neutral | 2.455 | medium | 0.115 | 0.042 | Neutral |
|  | Y764C | 0.19 | 0.003 | -1.943 | Neutral | -0.205 | neutral | 0.059 | 0.028 | Neutral |
|  | Y764H | 1 | 0 | 0.402 | Neutral | -1.545 | neutral | 0.029 | 0.012 | Neutral |
|  | L766F | 0.01 | 0.281 | -1.076 | Neutral | 1.1 | low | 0.293 | 0.06 | Unknown |
|  | G767R | 0.06 | 1 | -3.832 | Deleterious | 2.585 | medium | 0.667 | 0.073 | Unknown |
|  | S768A | 0.1 | 0.124 | -0.114 | Neutral |  |  | 0.044 | 0.019 | Neutral |
|  | S768R | 0.14 | 0.001 | -1.378 | Neutral | 1.32 | low | 0.054 | 0.02 | Neutral |
|  | W769C | 0 | 0.999 | -6.983 | Deleterious | 2.645 | medium | 0.514 | 0.073 | Unknown |
|  | G771E | 0.08 | 0.892 | -4.594 | Deleterious | 2.555 | medium | 0.311 | 0.065 | Unknown |
|  | G772D | 0.19 | 0.768 | -3.835 | Deleterious | 2.585 | medium | 0.393 | 0.098 | Unknown |
|  | K776E | 0.31 | 0.526 | -1.429 | Neutral | 2.24 | medium | 0.271 | 0.057 | Unknown |
|  | E777Q | 0.09 | 0.082 | -1.389 | Neutral | 2.155 | medium | 0.181 | 0.05 | Neutral |
|  | G778D | 0.06 | 0.058 | -1.895 | Neutral | 2.615 | medium | 0.218 | 0.049 | Neutral |
|  | W781L | 0.12 | 0.948 | -4.983 | Deleterious | 2.67 | medium | 0.435 | 0.059 | Unknown |
|  | G782A | 0.08 | 0.679 | -2.246 | Neutral | 2.52 | medium | 0.318 | 0.098 | Unknown |
|  | G782E | 0.16 | 0.912 | -2.869 | Deleterious | 2.52 | medium | 0.449 | 0.067 | Unknown |
|  | P784S | 0.13 | 0.466 | -2.549 | Deleterious | 2.395 | medium | 0.129 | 0.049 | Neutral |
|  | E785K | 0.01 | 0.617 | -2.035 | Neutral | 2.585 | medium | 0.229 | 0.053 | Neutral |
|  | P786R | 0.29 | 0.499 | -3.038 | Deleterious | 2.25 | medium | 0.193 | 0.059 | Neutral |
|  | P786T | 0.48 | 0.22 | -2.66 | Deleterious | 2.25 | medium | 0.248 | 0.058 | Unknown |
|  | G788E | 0.51 | 0.011 | -1.596 | Neutral | 0.945 | low | 0.062 | 0.022 | Neutral |
|  | D790N | 0.22 | 0.001 | -2.022 | Neutral | 0 | neutral | 0.297 | 0.071 | Unknown |
|  | D791Y | 0.01 | 0.518 | -2.457 | Neutral | 0.69 | neutral | 0.288 | 0.064 | Unknown |
|  | D791N | 1 | 0 | 0.17 | Neutral | -1.5 | neutral | 0.029 | 0.014 | Neutral |
|  | N792S | 0.32 | 0 | 0.124 | Neutral | -1.1 | neutral | 0.054 | 0.032 | Neutral |
|  | D793N | 0.11 | 0.027 | -2.157 | Neutral | 1.725 | low | 0.153 | 0.041 | Neutral |
|  | Q794R | 0.02 | 0.007 | -1.61 | Neutral | 1.63 | low | 0.172 | 0.042 | Neutral |
|  | L798F | 0.01 | 0.204 | -3.172 | Deleterious | 2.3 | medium | 0.306 | 0.066 | Unknown |
|  | F800C | 0 | 0.999 | -6.26 | Deleterious | 2.67 | medium | 0.779 | 0.053 | Pathogenic |
|  | S803A | 0.1 | 0.124 | -1.67 | Neutral | 2.34 | medium | 0.104 | 0.031 | Neutral |
|  | S804C | 0.03 | 0.874 | -2.698 | Deleterious |  |  | 0.233 | 0.048 | Neutral |
|  | R806H | 0.08 | 0.037 | -2.963 | Deleterious | 2.615 | medium | 0.624 | 0.084 | Unknown |
|  | R806C | 0.07 | 0.037 | -5.572 | Deleterious | 2.615 | medium | 0.746 | 0.079 | Unknown |
|  | R806L | 0.02 | 0.846 | -4.29 | Deleterious | 2.615 | medium | 0.619 | 0.066 | Unknown |
|  | W807C | 0 | 0.999 | -10.006 | Deleterious | 2.67 | medium | 0.823 | 0.046 | Pathogenic |
|  | E809Q | 0.2 | 0.474 | -0.933 | Neutral | 2.3 | medium | 0.181 | 0.053 | Neutral |
|  | H812Q | 0 | 0.885 | -4.546 | Deleterious | 1.97 | medium | 0.407 | 0.068 | Unknown |
|  | P813L | 0.34 | 0.999 | -6.206 | Deleterious | 2.3 | medium | 0.302 | 0.065 | Unknown |
|  | P813S | 0.01 | 0.999 | -5.498 | Deleterious | 2.645 | medium | 0.346 | 0.093 | Unknown |
|  | G814E | 0.18 | 0.912 | -4.356 | Deleterious | 2.52 | medium | 0.659 | 0.085 | Unknown |
|  | V816A | 0.03 | 0.582 | -2.606 | Deleterious | 2.555 | medium | 0.434 | 0.059 | Unknown |
|  | R818L | 0 | 0.999 | -5.192 | Deleterious | 2.67 | medium | 0.526 | 0.065 | Unknown |
|  | R818Q | 0.08 | 0.998 | -2.654 | Deleterious | 2.67 | medium | 0.225 | 0.069 | Unknown |
|  | I820V | 1 | 0 | 0.175 | Neutral | -1.75 | neutral | 0.07 | 0.037 | Neutral |
|  | A821G | 0 | 0.018 | -2.612 | Deleterious | 1.78 | low | 0.151 | 0.044 | Neutral |
|  | A821S | 0.01 | 0.046 | -1.28 | Neutral | 2.47 | medium | 0.117 | 0.038 | Neutral |
|  | A821T | 0.1 | 0.343 | -1.51 | Neutral | 2.47 | medium | 0.215 | 0.064 | Unknown |
|  | P822S | 0.09 | 0.021 | -3.23 | Deleterious | 0.785 | neutral | 0.121 | 0.031 | Neutral |
|  | P824L | 0.1 | 0.675 | -3.892 | Deleterious | 2.48 | medium | 0.366 | 0.099 | Unknown |
|  | P824R | 0.08 | 0.893 | -3.162 | Deleterious | 2.48 | medium | 0.404 | 0.087 | Unknown |
|  | P824S | 0.58 | 0.177 | -1.567 | Neutral | 1.68 | low | 0.122 | 0.034 | Neutral |
|  | A825P | 0.18 | 0 | 0.683 | Neutral | -1.245 | neutral | 0.028 | 0.014 | Neutral |
|  | A825T | 1 | 0 | 0.332 | Neutral | 0.55 | neutral | 0.058 | 0.028 | Neutral |
|  | T826I | 0.19 | 0.027 | -2.374 | Neutral | 2.44 | medium | 0.261 | 0.061 | Unknown |
|  | T826K | 0.64 | 0.49 | -1.436 | Neutral | 2.44 | medium | 0.351 | 0.06 | Unknown |
|  | T826S | 0.57 | 0.295 | -0.954 | Neutral | 2.44 | medium | 0.185 | 0.07 | Neutral |
|  | P827S | 0.85 | 0.031 | -4.087 | Deleterious | 1.725 | low | 0.152 | 0.037 | Neutral |
|  | P827A | 0.65 | 0.197 | -4.125 | Deleterious | 2.42 | medium | 0.086 | 0.034 | Neutral |
|  | M828I | 0.18 | 0.009 | -1.346 | Neutral | 1.7 | low | 0.09 | 0.039 | Neutral |
|  | M828V | 0.28 | 0 | -1.026 | Neutral | 1.7 | low | 0.114 | 0.059 | Neutral |
|  | D833E | 1 | 0.003 | -0.685 | Neutral | -0.255 | neutral | 0.024 | 0.012 | Neutral |
|  | D833H | 0.19 | 0.471 | -2.415 | Neutral | 1.1 | low | 0.279 | 0.087 | Unknown |
|  | G834D | 0.89 | 0.003 | -2.653 | Deleterious | 1.255 | low | 0.123 | 0.032 | Neutral |
|  | G834C | 0.32 | 0.73 | -4.002 | Deleterious | 2.3 | medium | 0.205 | 0.049 | Neutral |
|  | G834S | 0.6 | 0.007 | -2.017 | Neutral | 1.4 | low | 0.148 | 0.041 | Neutral |
|  | C837S | 0.54 | 0.005 | -1.984 | Neutral | 2.4 | medium | 0.195 | 0.054 | Neutral |
|  | S839C | 0.03 | 0.874 | -3.025 | Deleterious | 2.47 | medium | 0.266 | 0.106 | Unknown |
|  | R840P | 0.21 | 0.164 | -1.763 | Neutral | 0.895 | low | 0.475 | 0.118 | Unknown |
|  | R840Q | 1 | 0 | -0.283 | Neutral | -1.445 | neutral | 0.082 | 0.029 | Neutral |
|  | R840W | 0.03 | 0.436 | -4.556 | Deleterious |  |  | 0.445 | 0.068 | Unknown |
|  | R841H | 0.07 | 0.037 | -2.997 | Deleterious | 1.81 | low | 0.242 | 0.063 | Unknown |
|  | R841C | 0.07 | 0.037 | -4.61 | Deleterious | 2.505 | medium | 0.27 | 0.081 | Unknown |
|  | P842S | 0.05 | 0.876 | -2.435 | Neutral | 2.175 | medium | 0.149 | 0.04 | Neutral |
|  | L845V | 0.12 | 0.808 | -1.395 | Neutral | 2.585 | medium | 0.152 | 0.043 | Neutral |
|  | S850A | 0.99 | 0.448 | -1.433 | Neutral | 2.27 | medium | 0.223 | 0.057 | Neutral |
|  | L852F | 0 | 0.816 | -2.13 | Neutral | 2.22 | medium | 0.211 | 0.051 | Neutral |
|  | T853A | 0.43 | 0.495 | -2.175 | Neutral | 2.52 | medium | 0.281 | 0.062 | Unknown |
|  | Q855R | 0.15 | 0.006 | -2.083 | Neutral | 2.645 | medium | 0.144 | 0.038 | Neutral |
|  | N857K | 0.43 | 0.001 | -1.665 | Neutral | 0.345 | neutral | 0.162 | 0.05 | Neutral |
|  | T859I | 0.42 | 0.078 | -2.671 | Deleterious | 2.005 | medium | 0.202 | 0.051 | Neutral |
|  | T859S | 0.42 | 0.655 | -1.583 | Neutral | 2.555 | medium | 0.312 | 0.06 | Unknown |
|  | T859A | 0.51 | 0.655 | -2.341 | Neutral | 2.555 | medium | 0.168 | 0.05 | Neutral |
|  | L860P | 0 | 0.919 | -4.106 | Deleterious | 2.485 | medium | 0.809 | 0.065 | Pathogenic |
|  | S864C | 0.1 | 0.967 | -1.819 | Neutral | 1.995 | medium | 0.177 | 0.064 | Neutral |
|  | S864T | 0.12 | 0.809 | -0.917 | Neutral | 1.645 | low | 0.036 | 0.016 | Neutral |
|  | S864R | 0.25 | 0.003 | -1.854 | Neutral | 0.61 | neutral | 0.087 | 0.029 | Neutral |
|  | I868M | 0.1 | 0.077 | -1.098 | Neutral | 2.215 | medium | 0.063 | 0.024 | Neutral |
|  | I868V | 0.64 | 0.011 | -0.103 | Neutral | 1.12 | low | 0.056 | 0.024 | Neutral |
|  | V871M | 0.17 | 0.052 | -0.932 | Neutral | 1.69 | low | 0.066 | 0.024 | Neutral |
|  | L872I | 0.09 | 0.054 | -1.076 | Neutral |  |  | 0.17 | 0.045 | Neutral |
|  | D873G | 0.59 | 0 | -1.038 | Neutral | -0.95 | neutral | 0.098 | 0.051 | Neutral |
|  | K876N | 0.29 | 0.368 | -1.71 | Neutral | 2.19 | medium | 0.124 | 0.04 | Neutral |
|  | K876E | 0.65 | 0.006 | -1.423 | Neutral | 2.19 | medium | 0.202 | 0.054 | Neutral |
|  | S877A | 1 | 0 | 0.622 | Neutral | -2.35 | neutral | 0.056 | 0.026 | Neutral |
|  | P879L | 0.16 | 0.646 | -4.206 | Deleterious | 2.48 | medium | 0.328 | 0.088 | Unknown |
|  | E880K | 0.2 | 0.018 | -1.695 | Neutral | 1.81 | low | 0.069 | 0.03 | Neutral |
|  | D885Y | 0.01 | 0.477 | -3.008 | Deleterious | 2.08 | medium | 0.261 | 0.056 | Unknown |
|  | D885H | 0.24 | 0.013 | -1.67 | Neutral | 1.53 | low | 0.142 | 0.054 | Neutral |
|  | D885N | 0.22 | 0.003 | -1.302 | Neutral | 0.98 | low | 0.107 | 0.035 | Neutral |
|  | S888N | 0.07 | 0.046 | -0.895 | Neutral | 2.205 | medium | 0.345 | 0.073 | Unknown |
|  | D889V | 0.14 | 0.003 | -2.141 | Neutral | 1.445 | low | 0.171 | 0.049 | Neutral |
|  | D889G | 1 | 0 | -0.071 | Neutral | -0.33 | neutral | 0.089 | 0.048 | Neutral |
|  | N890S | 0.9 | 0.02 | -0.935 | Neutral | 1.19 | low | 0.112 | 0.044 | Neutral |
|  | D893G | 0.71 | 0.027 | -2.278 | Neutral | 1.655 | low | 0.171 | 0.064 | Neutral |
|  | S894Y | 0 | 0.565 | -3.931 | Deleterious | 2.615 | medium | 0.57 | 0.065 | Unknown |
|  | V896L | 0 | 0.991 | -1.943 | Neutral | 2.3 | medium | 0.383 | 0.059 | Unknown |
|  | V896M | 0 | 0.999 | -2.229 | Neutral | 2.645 | medium | 0.621 | 0.061 | Unknown |
|  | S897C | 0 | 0.943 | -3.808 | Deleterious | 2.19 | medium | 0.412 | 0.082 | Unknown |
|  | S897T | 0.03 | 0.072 | -2.206 | Neutral | 1.845 | low | 0.185 | 0.049 | Neutral |
|  | S899C | 0.1 | 0.967 | -3.27 | Deleterious | 2.615 | medium | 0.318 | 0.068 | Unknown |
|  | S899T | 0.12 | 0.809 | -1.705 | Neutral | 2.27 | medium | 0.151 | 0.039 | Neutral |
|  | A900V | 0 | 0.998 | -2.927 | Deleterious | 2.615 | medium | 0.281 | 0.085 | Unknown |
|  | A900T | 0 | 0.998 | -2.53 | Deleterious | 2.615 | medium | 0.149 | 0.07 | Neutral |
|  | E901Q | 0.02 | 0.814 | -1.705 | Neutral | 2.535 | medium | 0.284 | 0.062 | Unknown |
|  | L903S | 0 | 0.858 | -3.044 | Deleterious | 2.505 | medium | 0.162 | 0.036 | Neutral |
|  | S904T | 0.07 | 0.359 | -1.778 | Neutral | 2.585 | medium | 0.095 | 0.031 | Neutral |
|  | R905Q | 0 | 0.994 | -2.663 | Deleterious | 2.515 | medium | 0.167 | 0.046 | Neutral |
|  | R905W | 0 | 0.999 | -6.098 | Deleterious | 2.515 | medium | 0.435 | 0.064 | Unknown |
|  | L907I | 0.09 | 0.054 | -0.897 | Neutral | 2.095 | medium | 0.053 | 0.018 | Neutral |
|  | P910L | 0 | 0.801 | -5.397 | Deleterious | 2.47 | medium | 0.27 | 0.063 | Unknown |
|  | P910H | 0 | 0.917 | -3.933 | Deleterious | 2.47 | medium | 0.218 | 0.045 | Neutral |
|  | L911P | 0 | 0.976 | 0.346 | Neutral | 1.385 | low | 0.139 | 0.046 | Neutral |
|  | L911F | 0.65 | 0.006 | -2.052 | Neutral | 1.295 | low | 0.099 | 0.033 | Neutral |
|  | A913V | 0.38 | 0.091 | -1.808 | Neutral | 1.78 | low | 0.107 | 0.036 | Neutral |
|  | A913E | 0.23 | 0.149 | -1.432 | Neutral | 1.43 | low | 0.177 | 0.043 | Neutral |
|  | A913T | 0.42 | 0.007 | -0.657 | Neutral | 0.74 | neutral | 0.237 | 0.068 | Unknown |
|  | L915F | 0.56 | 0.003 | -1.062 | Neutral | 0.145 | neutral | 0.106 | 0.033 | Neutral |
|  | K918N | 0.02 | 0.625 | -1.848 | Neutral | 2.19 | medium | 0.115 | 0.031 | Neutral |
|  | N921K | 0.07 | 0.087 | -2.613 | Deleterious | 1.795 | low | 0.145 | 0.04 | Neutral |
|  | T922M | 0.13 | 0.385 | -1.702 | Neutral | 1.79 | low | 0.087 | 0.034 | Neutral |
|  | G923W | 0.02 | 0.795 | -2.625 | Deleterious | 1.905 | low | 0.279 | 0.065 | Unknown |
|  | A924V | 0.04 | 0.025 | -1.579 | Neutral | 2.47 | medium | 0.104 | 0.055 | Neutral |
|  | A924T | 0.22 | 0.015 | -1.302 | Neutral | 1.92 | low | 0.06 | 0.023 | Neutral |
|  | R926K | 0.69 | 0.011 | -0.538 | Neutral | 1.75 | low | 0.141 | 0.04 | Neutral |
|  | C928F | 0.02 | 0.66 | -4.298 | Deleterious | 2.4 | medium | 0.215 | 0.054 | Neutral |
|  | C928Y | 0.02 | 0.66 | -4.084 | Deleterious | 2.4 | medium | 0.251 | 0.049 | Neutral |
|  | V929G | 0.01 | 0.998 | -4.765 | Deleterious | 2.595 | medium | 0.393 | 0.067 | Unknown |
|  | V929M | 0.03 | 0.999 | -2.079 | Neutral | 2.595 | medium | 0.499 | 0.075 | Unknown |
|  | T932I | 0 | 0.816 | -4.013 | Deleterious | 2.535 | medium | 0.268 | 0.065 | Unknown |
|  | A934T | 0.04 | 0.358 | -1.368 | Neutral | 2.075 | medium | 0.254 | 0.075 | Unknown |
|  | L935F | 0.02 | 0.79 | -2.176 | Neutral | 2.36 | medium | 0.119 | 0.043 | Neutral |
|  | T937I | 0.48 | 0.007 | -1.706 | Neutral | 2.3 | medium | 0.163 | 0.062 | Neutral |
|  | T937S | 0.24 | 0.085 | -0.733 | Neutral | 2.3 | medium | 0.049 | 0.025 | Neutral |
|  | E939D | 1 | 0 | 0.343 | Neutral | -1.835 | neutral | 0.029 | 0.013 | Neutral |
|  | C940Y | 0.2 | 0.003 | -4.251 | Deleterious | 1.14 | low | 0.141 | 0.057 | Neutral |
|  | F942L | 0.15 | 0.088 | -2.144 | Neutral | 2.2 | medium | 0.143 | 0.052 | Neutral |
|  | F942I | 0.09 | 0.283 | -2.168 | Neutral | 2.2 | medium | 0.289 | 0.051 | Unknown |
|  | P943L | 0.02 | 0.018 | -6.168 | Deleterious | 1.63 | low | 0.275 | 0.084 | Unknown |
|  | P943R | 0.11 | 0.012 | -5.594 | Deleterious | 1.08 | low | 0.219 | 0.053 | Neutral |
|  | P944S | 0.42 | 0.509 | -2.403 | Neutral | 2.52 | medium | 0.125 | 0.041 | Neutral |
|  | L946P | 0 | 0.976 | -5.121 | Deleterious | 2.585 | medium | 0.897 | 0.037 | Pathogenic |
|  | Y949C | 0 | 0.999 | -7.543 | Deleterious | 2.67 | medium | 0.836 | 0.048 | Pathogenic |
|  | I950M | 0.07 | 0.127 | -0.562 | Neutral | 1.64 | low | 0.191 | 0.056 | Neutral |
|  | I950V | 0.04 | 0.019 | -0.473 | Neutral | 2.535 | medium | 0.194 | 0.046 | Neutral |
|  | D952E | 1 | 0.003 | -0.492 | Neutral | 1.645 | low | 0.034 | 0.016 | Neutral |
|  | P953L | 0.01 | 0.998 | -4.325 | Deleterious | 2.585 | medium | 0.384 | 0.106 | Unknown |
|  | P956L | 0.02 | 0.903 | -4.794 | Deleterious | 2.645 | medium | 0.361 | 0.08 | Unknown |
|  | P957L | 0.65 | 0.003 | -1.13 | Neutral | 1.795 | low | 0.362 | 0.071 | Unknown |
|  | P957S | 0.4 | 0.012 | -1.457 | Neutral | 1.645 | low | 0.226 | 0.06 | Neutral |
|  | L961V | 0.06 | 0.258 | -2.8 | Deleterious | 2.67 | medium | 0.357 | 0.062 | Unknown |
|  | P963L | 0 | 0.998 | -9.063 | Deleterious | 2.67 | medium | 0.636 | 0.102 | Unknown |
|  | P963A | 0 | 0.998 | -7.07 | Deleterious | 2.67 | medium | 0.689 | 0.086 | Unknown |
|  | R966P | 0.01 | 0.838 | -3.406 | Deleterious | 2.585 | medium | 0.815 | 0.061 | Pathogenic |
|  | R966Q | 0.15 | 0.07 | -1.559 | Neutral | 2.585 | medium | 0.317 | 0.088 | Unknown |
|  | R966W | 0 | 0.928 | -4.451 | Deleterious | 2.585 | medium | 0.589 | 0.111 | Unknown |
|  | L972F | 0 | 0.998 | -2.844 | Deleterious | 2.3 | medium | 0.211 | 0.052 | Neutral |
|  | R975S | 0.01 | 0.23 | -3.819 | Deleterious | 2.39 | medium | 0.464 | 0.069 | Unknown |
|  | R975G | 0.01 | 0.372 | -5.273 | Deleterious | 2.39 | medium | 0.546 | 0.085 | Unknown |
|  | V976F | 0 | 0.043 | -3.959 | Deleterious | 2.43 | medium | 0.55 | 0.078 | Unknown |
|  | N981S | 0.03 | 0.346 | -2.817 | Deleterious | 2.175 | medium | 0.197 | 0.059 | Neutral |
|  | V982I | 0.04 | 0.058 | -0.829 | Neutral | 1.905 | low | 0.266 | 0.07 | Unknown |
|  | Y983C | 0 | 0.999 | -8.543 | Deleterious | 2.67 | medium | 0.845 | 0.048 | Pathogenic |
|  | Y983H | 0 | 0.998 | -4.698 | Deleterious | 2.67 | medium | 0.693 | 0.071 | Unknown |
|  | R987Q | 0.07 | 0.049 | -0.498 | Neutral | 1.245 | low | 0.15 | 0.055 | Neutral |
|  | R987W | 0.02 | 0.88 | -2.759 | Deleterious | 2.34 | medium | 0.587 | 0.101 | Unknown |
|  | S989F | 0 | 0.943 | -3.771 | Deleterious | 2.555 | medium | 0.462 | 0.085 | Unknown |
|  | T990A | 0.01 | 0.51 | -1.921 | Neutral | 2.555 | medium | 0.282 | 0.047 | Neutral |
|  | Y991C | 0.22 | 0.035 | -0.34 | Neutral | 1.4 | low | 0.2 | 0.049 | Neutral |
|  | Q993R | 0.26 | 0.015 | -1.163 | Neutral | 1.395 | low | 0.158 | 0.039 | Neutral |
|  | Q993K | 0.07 | 0.178 | -1.363 | Neutral | 2.43 | medium | 0.242 | 0.068 | Unknown |
|  | S996R | 0 | 0.55 | -3.621 | Deleterious | 2.565 | medium | 0.278 | 0.053 | Unknown |
|  | F997C | 0 | 0.998 | -3.687 | Deleterious | 2.585 | medium | 0.706 | 0.071 | Unknown |
|  | P998L | 0 | 0.998 | -5.413 | Deleterious | 2.645 | medium | 0.538 | 0.08 | Unknown |
|  | P999L | 0.14 | 0.007 | -3.32 | Deleterious | 1.5 | low | 0.354 | 0.088 | Unknown |
|  | P999H | 0 | 0.639 | -3.17 | Deleterious | 2.39 | medium | 0.455 | 0.079 | Unknown |
|  | E1000K | 0.2 | 0.624 | -2.248 | Neutral | 2.36 | medium | 0.271 | 0.065 | Unknown |
|  | I1003V | 1 | 0 | 0.175 | Neutral | -0.09 | neutral | 0.059 | 0.023 | Neutral |
|  | I1005V | 1 | 0 | 0.362 | Neutral | -1.7 | neutral | 0.02 | 0.011 | Neutral |
|  | P1008R | 0.04 | 0.999 | -5.724 | Deleterious | 2.645 | medium | 0.576 | 0.096 | Unknown |
|  | H1009R | 0.22 | 0.015 | -2.962 | Deleterious | 2.505 | medium | 0.304 | 0.057 | Unknown |
|  | I1010M | 0 | 0.79 | -0.978 | Neutral | 2.035 | medium | 0.229 | 0.044 | Neutral |
|  | I1010N | 0 | 0.912 | -4.238 | Deleterious | 2.38 | medium | 0.546 | 0.09 | Unknown |
|  | I1010V | 0.73 | 0.015 | -0.178 | Neutral | 1.03 | low | 0.225 | 0.068 | Unknown |
|  | I1010L | 0.21 | 0.178 | -0.892 | Neutral | 2.38 | medium | 0.194 | 0.043 | Neutral |
|  | Y1011S | 0.01 | 0.494 | -3.976 | Deleterious | 2.38 | medium | 0.398 | 0.076 | Unknown |
|  | A1013V | 0 | 0.974 | -2.165 | Neutral | 2.585 | medium | 0.259 | 0.064 | Unknown |
|  | A1013T | 0.11 | 0.947 | -2.165 | Neutral | 2.585 | medium | 0.241 | 0.093 | Unknown |
|  | E1014K | 0.1 | 0.359 | -2.098 | Neutral | 2.505 | medium | 0.344 | 0.087 | Unknown |
|  | Q1017H | 0 | 0.997 | -0.697 | Neutral | 1.645 | low | 0.232 | 0.068 | Unknown |
|  | Q1017P | 0 | 0.996 | -2.235 | Neutral | 2.34 | medium | 0.568 | 0.082 | Unknown |
|  | A1025V | 0.04 | 0.884 | -2.543 | Deleterious | 2.645 | medium | 0.298 | 0.089 | Unknown |
|  | A1027T | 0.56 | 0.028 | 0.359 | Neutral | 1.79 | low | 0.113 | 0.048 | Neutral |
|  | S1028F | 0.19 | 0.998 | -2.962 | Deleterious | 2.585 | medium | 0.265 | 0.106 | Unknown |
|  | C1029Y | 0 | 0.998 | -7.787 | Deleterious | 2.645 | medium | 0.655 | 0.061 | Unknown |
|  | H1030R | 0 | 0.996 | -5.33 | Deleterious | 2.645 | medium | 0.694 | 0.069 | Unknown |
|  | V1032I | 0.04 | 0.992 | -0.708 | Neutral | 2.645 | medium | 0.23 | 0.055 | Neutral |
|  | S1033F | 0.71 | 0.022 | -3.184 | Deleterious | 2.48 | medium | 0.584 | 0.062 | Unknown |
|  | S1033C | 0.32 | 0.035 | 0.28 | Neutral | 1.79 | low | 0.227 | 0.064 | Unknown |
|  | S1036G | 0.01 | 0.475 | -2.856 | Deleterious | 2.585 | medium | 0.311 | 0.07 | Unknown |
|  | Q1038R | 0.21 | 0.294 | -1.997 | Neutral | 2.075 | medium | 0.166 | 0.043 | Neutral |
|  | Q1038P | 0.04 | 0.648 | -3.746 | Deleterious | 2.42 | medium | 0.681 | 0.076 | Unknown |
|  | L1039V | 0.06 | 0.324 | -2.267 | Neutral | 2.585 | medium | 0.435 | 0.082 | Unknown |
|  | W1041R | 0 | 0.999 | -10.625 | Deleterious | 2.67 | medium | 0.852 | 0.047 | Pathogenic |
|  | V1042A | 0.02 | 0.046 | -2.54 | Deleterious | 2.505 | medium | 0.447 | 0.061 | Unknown |
|  | T1047I | 0 | 0.408 | -2.962 | Deleterious | 2.505 | medium | 0.238 | 0.064 | Unknown |
|  | T1047N | 0.2 | 0.037 | -0.968 | Neutral | 2.505 | medium | 0.429 | 0.071 | Unknown |
|  | T1047A | 0.04 | 0.164 | -1.492 | Neutral | 2.505 | medium | 0.332 | 0.109 | Unknown |
|  | I1049M | 0 | 0.181 | -2.244 | Neutral | 1.65 | low | 0.196 | 0.048 | Neutral |
|  | C1050F | 0.82 | 0.912 | -2.967 | Deleterious | 1.92 | low | 0.325 | 0.061 | Unknown |
|  | R1051Q | 0.46 | 0.037 | -0.116 | Neutral | 0.345 | neutral | 0.166 | 0.054 | Neutral |
|  | R1051W | 0.03 | 0.436 | -2.256 | Neutral | 0.345 | neutral | 0.438 | 0.094 | Unknown |
|  | Q1052H | 0 | 0.997 | -3.054 | Deleterious | 2.615 | medium | 0.415 | 0.087 | Unknown |
|  | Q1052P | 0 | 0.996 | -3.83 | Deleterious | 2.615 | medium | 0.658 | 0.08 | Unknown |
|  | K1054R | 1 | 0 | -0.389 | Neutral | -1.2 | neutral | 0.062 | 0.023 | Neutral |
|  | K1054Q | 0.09 | 0.009 | -0.786 | Neutral | 0.6 | neutral | 0.069 | 0.022 | Neutral |
|  | C1055S | 0 | 0.998 | -8.079 | Deleterious | 2.67 | medium | 0.786 | 0.073 | Unknown |
|  | C1055G | 0 | 0.998 | -9.59 | Deleterious | 2.67 | medium | 0.82 | 0.042 | Pathogenic |
|  | R1057H | 0.13 | 0.007 | -2.938 | Deleterious | 1.295 | low | 0.148 | 0.053 | Neutral |
|  | R1057C | 0.01 | 0.742 | -4.762 | Deleterious | 2.045 | medium | 0.324 | 0.082 | Unknown |
|  | L1058R | 0.1 | 0 | 0.76 | Neutral | -0.345 | neutral | 0.463 | 0.077 | Unknown |
|  | G1059V | 0.02 | 0.651 | -3.81 | Deleterious | 2.125 | medium | 0.205 | 0.06 | Neutral |
|  | S1060F | 0 | 0.221 | -2.787 | Deleterious | 1.1 | low | 0.204 | 0.05 | Neutral |
|  | C1062W | 0* | 1 | -8.025 | Deleterious | 2.67 | medium | 0.506 | 0.139 | Unknown |
|  | C1062Y | 0 | 0.999 | -8.121 | Deleterious | 2.67 | medium | 0.873 | 0.063 | Pathogenic |
|  | P1063S | 0.08 | 0.021 | -4.206 | Deleterious | 0.87 | low | 0.146 | 0.038 | Neutral |
|  | T1064M | 0 | 0.882 | -3.148 | Deleterious | 2.3 | medium | 0.416 | 0.084 | Unknown |
|  | T1064A | 0.16 | 0.018 | -1.858 | Neutral | 1.4 | low | 0.22 | 0.053 | Neutral |
|  | T1066R | 0.03 | 0.015 | -2.66 | Deleterious | 1.935 | low | 0.391 | 0.062 | Unknown |
|  | T1066S | 0.03 | 0.124 | -1.017 | Neutral | 1.935 | low | 0.073 | 0.026 | Neutral |
|  | A1067S | 1 | 0.003 | 0.344 | Neutral | -1.65 | neutral | 0.038 | 0.017 | Neutral |
|  | I1068V | 1 | 0.001 | 0.699 | Neutral | -0.895 | neutral | 0.094 | 0.041 | Neutral |
|  | Q1070R | 0.1 | 0.996 | -2.07 | Neutral | 2.3 | medium | 0.56 | 0.076 | Unknown |
|  | I1072V | 0.03 | 0.015 | 0.032 | Neutral | 0 | neutral | 0.081 | 0.028 | Neutral |
|  | R1074S | 0.1 | 0.047 | -3.594 | Deleterious | 1.875 | low | 0.19 | 0.05 | Neutral |
|  | R1074K | 0.07 | 0.199 | -1.421 | Neutral | 2.565 | medium | 0.156 | 0.053 | Neutral |
|  | L1075P | 0 | 0.919 | -4.541 | Deleterious | 2.535 | medium | 0.811 | 0.068 | Pathogenic |
|  | L1075F | 0.09 | 0.069 | -2.229 | Neutral | 1.845 | low | 0.164 | 0.044 | Neutral |
|  | T1081I | 0 | 0.998 | -5.152 | Deleterious | 2.645 | medium | 0.871 | 0.04 | Pathogenic |
|  | E1083K | 0 | 0.541 | -3.01 | Deleterious | 2.595 | medium | 0.768 | 0.065 | Unknown |
|  | V1085L | 0.71 | 0.007 | -0.462 | Neutral | 1.295 | low | 0.114 | 0.034 | Neutral |
|  | V1085M | 0.2 | 0.055 | -0.23 | Neutral | 1.09 | low | 0.142 | 0.036 | Neutral |
|  | V1086M | 0 | 0.943 | -2.1 | Neutral | 2.645 | medium | 0.794 | 0.058 | Pathogenic |
|  | C1088R | 0 | 0.998 | -7.686 | Deleterious | 2.615 | medium | 0.828 | 0.04 | Pathogenic |
|  | N1090S | 0 | 0.275 | -2.303 | Neutral | 2.36 | medium | 0.229 | 0.06 | Neutral |
|  | H1091Q | 0.61 | 0.135 | -2.235 | Neutral | 1.74 | low | 0.145 | 0.041 | Neutral |
|  | H1091Y | 0.02 | 0.031 | -3.367 | Deleterious | 1.74 | low | 0.216 | 0.049 | Neutral |
|  | H1092G | 0.53 | 0.427 | -4.74 | Deleterious |  |  | 0.359 | 0.072 | Unknown |
|  | A1094V | 0 | 0.633 | -2.213 | Neutral | 2.535 | medium | 0.218 | 0.063 | Neutral |
|  | A1094T | 0.02 | 0.137 | -2.022 | Neutral | 1.845 | low | 0.203 | 0.053 | Neutral |
|  | A1096T | 0.19 | 0.104 | -1.902 | Neutral | 2.155 | medium | 0.325 | 0.069 | Unknown |
|  | L1097P | 0 | 0.999 | -5.884 | Deleterious | 2.67 | medium | 0.916 | 0.034 | Pathogenic |
|  | G1098R | 0 | 1 | -4.854 | Deleterious | 2.67 | medium | 0.795 | 0.061 | Pathogenic |
|  | P1101T | 0.17 | 0.992 | -3.187 | Deleterious | 2.505 | medium | 0.397 | 0.083 | Unknown |
|  | P1101R | 0.02 | 0.936 | -3.729 | Deleterious | 2.505 | medium | 0.555 | 0.1 | Unknown |
|  | R1102K | 0.61 | 0.006 | 0.21 | Neutral | 0.345 | neutral | 0.058 | 0.02 | Neutral |
|  | E1103D | 0.06 | 0.994 | -1.994 | Neutral | 2.585 | medium | 0.615 | 0.102 | Unknown |
|  | S1106Y | 0.13 | 0.912 | -3.437 | Deleterious | 2.43 | medium | 0.532 | 0.083 | Unknown |
|  | L1107F | 0 | 0.858 | -2.165 | Neutral | 2.555 | medium | 0.527 | 0.071 | Unknown |
|  | L1108P | 0.02 | 0.967 | -1.837 | Neutral | 2.52 | medium | 0.896 | 0.036 | Pathogenic |
|  | D1109G | 0.06 | 0.109 | -2.005 | Neutral | 0.345 | neutral | 0.183 | 0.042 | Neutral |
|  | D1109H | 0.02 | 0.471 | -1.18 | Neutral | 0.345 | neutral | 0.429 | 0.078 | Unknown |
|  | V1111L | 0.24 | 0.971 | -1.862 | Neutral | 2.125 | medium | 0.348 | 0.078 | Unknown |
|  | V1111I | 0.05 | 0.651 | -0.684 | Neutral | 2.47 | medium | 0.368 | 0.068 | Unknown |
|  | V1113M | 0.11 | 0.371 | -0.524 | Neutral | 1.355 | low | 0.243 | 0.068 | Unknown |
|  | P1114S | 0.08 | 0.998 | -2.949 | Deleterious | 2.585 | medium | 0.528 | 0.09 | Unknown |
|  | V1117A | 0 | 0.994 | -3.022 | Deleterious | 2.645 | medium | 0.588 | 0.067 | Unknown |
|  | V1117L | 0.05 | 0.991 | -1.195 | Neutral | 2.3 | medium | 0.328 | 0.076 | Unknown |
|  | Q1120H | 0.14 | 0.01 | -1.856 | Neutral | 1.465 | low | 0.167 | 0.046 | Neutral |
|  | G1123R | 0.13 | 0.018 | -2.283 | Neutral | 1.41 | low | 0.287 | 0.089 | Unknown |
|  | P1124S | 0.1 | 0.007 | -1.843 | Neutral | 2.48 | medium | 0.153 | 0.064 | Neutral |
|  | Q1127H | 0.1 | 0.01 | -1.189 | Neutral | 1.32 | low | 0.259 | 0.078 | Unknown |
|  | L1128V | 1 | 0.003 | -0.229 | Neutral | 1.905 | low | 0.04 | 0.017 | Neutral |
|  | S1130P | 0.29 | 0.007 | -1.405 | Neutral | 2.52 | medium | 0.547 | 0.098 | Unknown |
|  | S1131L | 0.07 | 0.23 | -1.597 | Neutral | 2.4 | medium | 0.124 | 0.038 | Neutral |
|  | R1133K | 1 | 0 | 0.116 | Neutral | -1.935 | neutral | 0.041 | 0.019 | Neutral |
|  | R1133G | 0.17 | 0.025 | -1.562 | Neutral | 0.345 | neutral | 0.434 | 0.127 | Unknown |
|  | V1134A | 0.6 | 0.025 | -0.746 | Neutral | 1.1 | low | 0.152 | 0.036 | Neutral |
|  | V1134F | 0.36 | 0.112 | -1.417 | Neutral | 1.1 | low | 0.178 | 0.048 | Neutral |
|  | D1135E | 0.86 | 0.013 | -0.636 | Neutral | 1.255 | low | 0.065 | 0.025 | Neutral |
|  | D1135G | 0.47 | 0.003 | -2.534 | Deleterious | 1.95 | medium | 0.209 | 0.046 | Neutral |
|  | D1135N | 0.26 | 0.003 | -1.835 | Neutral | 2.3 | medium | 0.164 | 0.041 | Neutral |
|  | E1136K | 0.04 | 0.029 | -1.675 | Neutral | 1.545 | low | 0.26 | 0.062 | Unknown |
|  | P1137R | 0.01 | 0.868 | -3.989 | Deleterious | 2.615 | medium | 0.554 | 0.088 | Unknown |
|  | M1138I | 0.22 | 0.003 | -0.276 | Neutral | -0.345 | neutral | 0.127 | 0.04 | Neutral |
|  | M1138K | 0 | 0.065 | -2.216 | Neutral | 0 | neutral | 0.312 | 0.118 | Unknown |
|  | M1138V | 0.42 | 0 | -0.519 | Neutral | 0 | neutral | 0.094 | 0.031 | Neutral |
|  | T1139A | 0.48 | 0.025 | -0.805 | Neutral | 1.355 | low | 0.063 | 0.023 | Neutral |
|  | M1140I | 0.18 | 0 | -0.448 | Neutral | 0 | neutral | 0.109 | 0.033 | Neutral |
|  | M1140T | 0.11 | 0 | -1.556 | Neutral | 0 | neutral | 0.267 | 0.056 | Unknown |
|  | M1140K | 0.08 | 0 | -2.09 | Neutral | 0 | neutral | 0.268 | 0.07 | Unknown |
|  | M1140V | 0.2 | 0 | -0.592 | Neutral | 0 | neutral | 0.1 | 0.034 | Neutral |
|  | F1141L | 0.2 | 0.027 | -3.188 | Deleterious | 1.92 | low | 0.255 | 0.054 | Neutral |
|  | L1142H | 0 | 0.999 | -4.767 | Deleterious | 2.645 | medium | 0.768 | 0.065 | Unknown |
|  | L1142P | 0 | 0.999 | -4.325 | Deleterious | 2.645 | medium | 0.918 | 0.03 | Pathogenic |
|  | T1144I | 0 | 0.563 | -2.662 | Deleterious | 2.47 | medium | 0.318 | 0.082 | Unknown |
|  | L1145R | 0 | 0.999 | -4.13 | Deleterious | 2.645 | medium | 0.881 | 0.034 | Pathogenic |
|  | C1146R | 0 | 0.998 | -6.933 | Deleterious | 2.645 | medium | 0.63 | 0.065 | Unknown |
|  | T1147I | 0.16 | 0.901 | -3.289 | Deleterious | 2.43 | medium | 0.299 | 0.083 | Unknown |
|  | T1147S | 0.81 | 0.085 | -1.012 | Neutral | 1.74 | low | 0.089 | 0.034 | Neutral |
|  | T1147A | 0.53 | 0.135 | -1.929 | Neutral | 1.74 | low | 0.145 | 0.045 | Neutral |
|  | P1149L | 0.45 | 0.003 | -4.621 | Deleterious | 0.995 | low | 0.079 | 0.032 | Neutral |
|  | V1151A | 0.07 | 0.043 | -2.724 | Deleterious | 1.875 | low | 0.258 | 0.061 | Unknown |
|  | R1153P | 0 | 0.999 | -4.624 | Deleterious | 2.67 | medium | 0.859 | 0.041 | Pathogenic |
|  | R1153H | 0 | 0.998 | -3.214 | Deleterious | 2.67 | medium | 0.608 | 0.063 | Unknown |
|  | R1153C | 0 | 0.998 | -4.876 | Deleterious | 2.67 | medium | 0.645 | 0.061 | Unknown |
|  | I1155V | 0.24 | 0.003 | -0.326 | Neutral | 0.46 | neutral | 0.058 | 0.026 | Neutral |
|  | L1157F | 0.02 | 0.208 | -2.295 | Neutral | 2.3 | medium | 0.166 | 0.05 | Neutral |
|  | S1158A | 0.36 | 0.009 | -1.262 | Neutral | 1.78 | low | 0.121 | 0.035 | Neutral |
|  | E1160D | 0.11 | 0.6 | -1.138 | Neutral | 2.48 | medium | 0.197 | 0.053 | Neutral |
|  | E1160G | 0.02 | 0.439 | -2.386 | Neutral | 2.48 | medium | 0.187 | 0.05 | Neutral |
|  | K1164N | 0.1 | 0.714 | -1.952 | Neutral | 2.43 | medium | 0.206 | 0.042 | Neutral |
|  | P1165L | 0 | 0.898 | -5.19 | Deleterious | 2.615 | medium | 0.405 | 0.134 | Unknown |
|  | S1166L | 0.1 | 0.187 | -2.806 | Deleterious | 2.43 | medium | 0.163 | 0.054 | Neutral |
|  | K1167T | 0.01 | 0.08 | -2.343 | Neutral | 1.79 | low | 0.109 | 0.032 | Neutral |
|  | V1169I | 0.39 | 0 | -0.07 | Neutral | -1.79 | neutral | 0.033 | 0.016 | Neutral |
|  | P1170S | 0.11 | 0.801 | -2.4 | Neutral | 2.585 | medium | 0.135 | 0.042 | Neutral |
|  | P1170A | 0.08 | 0.475 | -2.71 | Deleterious | 2.585 | medium | 0.092 | 0.033 | Neutral |
|  | L1171F | 0.14 | 0.715 | -1.32 | Neutral | 2.39 | medium | 0.169 | 0.08 | Unknown |
|  | R1175Q | 0.77 | 0.121 | -1.127 | Neutral | 1.61 | low | 0.131 | 0.038 | Neutral |
|  | R1175W | 0 | 0.969 | -4.003 | Deleterious | 2.505 | medium | 0.642 | 0.073 | Unknown |
|  | R1178L | 0.01 | 0.773 | -3.195 | Deleterious | 2.47 | medium | 0.579 | 0.065 | Unknown |
|  | R1178P | 0 | 0.906 | -3.433 | Deleterious | 2.47 | medium | 0.716 | 0.072 | Unknown |
|  | R1178Q | 0.17 | 0.049 | -1.576 | Neutral | 1.43 | low | 0.227 | 0.066 | Unknown |
|  | R1178G | 0.01 | 0.823 | -3.613 | Deleterious | 2.47 | medium | 0.638 | 0.076 | Unknown |
|  | L1187P | 0.02 | 0.987 | -3.243 | Deleterious | 2.615 | medium | 0.826 | 0.052 | Pathogenic |
|  | T1188I | 0 | 0.998 | -3.705 | Deleterious | 2.645 | medium | 0.626 | 0.074 | Unknown |
|  | H1189Q | 0.43 | 0.151 | -0.775 | Neutral | 1.01 | low | 0.277 | 0.058 | Unknown |
|  | H1189Y | 0.01 | 0.157 | -1.77 | Neutral | 1.355 | low | 0.256 | 0.063 | Unknown |
|  | V1190M | 0.15 | 0.124 | -0.971 | Neutral | 2.19 | medium | 0.396 | 0.082 | Unknown |
|  | R1195L | 0.06 | 0.151 | -2.802 | Deleterious | 2.34 | medium | 0.283 | 0.094 | Unknown |
|  | R1195Q | 0.45 | 0.005 | -0.984 | Neutral | 1.1 | low | 0.11 | 0.044 | Neutral |
|  | R1195G | 0.06 | 0.151 | -2.278 | Neutral | 2.34 | medium | 0.362 | 0.091 | Unknown |
|  | I1201V | 0.56 | 0.015 | -0.443 | Neutral | 2 | medium | 0.216 | 0.058 | Neutral |
|  | I1201L | 0.3 | 0.003 | -0.397 | Neutral | 0.965 | low | 0.232 | 0.052 | Neutral |
|  | R1202Q | 1 | 0.001 | -0.352 | Neutral | -1.01 | neutral | 0.127 | 0.04 | Neutral |
|  | S1207F | 0.08 | 0.001 | -1.659 | Neutral | 0.69 | neutral | 0.147 | 0.038 | Neutral |
|  | S1207Y | 0.01 | 0.081 | -1.426 | Neutral | 0.69 | neutral | 0.299 | 0.05 | Unknown |
|  | S1208N | 0.1 | 0.003 | 0.032 | Neutral | 1.61 | low | 0.081 | 0.048 | Neutral |
|  | L1210V | 0.12 | 0.003 | -0.807 | Neutral | 1.79 | low | 0.107 | 0.038 | Neutral |
|  | A1214V | 0.18 | 0.003 | -1.724 | Neutral | 1.355 | low | 0.065 | 0.028 | Neutral |
|  | C1217Y | 0 | 0 | -2.5 | Deleterious | 1.095 | low | 0.156 | 0.046 | Neutral |
|  | C1217R | 0 | 0.003 | -2.229 | Neutral | 1.795 | low | 0.188 | 0.046 | Neutral |

**Supplementary Table S2:** Structure based prediction of CTC1 mutations.

| **S. No.** | **AA**  **Substitution** | **mCSM**  **(kcal/mol)** | **Stability** | **SDM Score** | **Stability** | **DUET Score** | **Stability** | **MAESTRO (ΔΔ*G*pred.)** | **MAESTRO (cpred.)** | **STRUM  (ΔΔ*G*)** |
| --- | --- | --- | --- | --- | --- | --- | --- | --- | --- | --- |
|  | P725R | 0.187 | Stabilizing | 0 | Destabilizing | 0.385 | Stabilizing | 0.228 | 0.915 | -0.23 |
|  | H726R | -0.571 | Destabilizing | -0.55 | Destabilizing | -0.411 | Destabilizing | 0.143 | 0.94 | -0.92 |
|  | G728E | -1.951 | Destabilizing | -1.97 | Destabilizing | -2.034 | Destabilizing | 0.408 | 0.911 | -1.96 |
|  | G728R | -1.086 | Destabilizing | -2.38 | Destabilizing | -1.032 | Destabilizing | 0.978 | 0.907 | -1.91 |
|  | Q729H | -1.479 | Destabilizing | 0.9 | Stabilizing | -0.955 | Destabilizing | 0.298 | 0.93 | -1.46 |
|  | S730R | -1.112 | Destabilizing | -0.66 | Destabilizing | -0.873 | Destabilizing | 1.129 | 0.91 | -1.77 |
|  | S730G | -1.965 | Destabilizing | 0.75 | Stabilizing | -1.576 | Destabilizing | 0.834 | 0.906 | -1.34 |
|  | R731L | -1.401 | Destabilizing | 0.82 | Stabilizing | -0.916 | Destabilizing | 0.3 | 0.964 | -1.1 |
|  | R731Q | -1.49 | Destabilizing | -2.09 | Destabilizing | -1.831 | Destabilizing | -0.542 | 0.829 | -1.63 |
|  | R731W | -1.05 | Destabilizing | 0.92 | Stabilizing | -0.634 | Destabilizing | 1.017 | 0.868 | -1.91 |
|  | C736R | -0.711 | Destabilizing | 0.15 | Stabilizing | -0.215 | Destabilizing | 2.281 | 0.858 | -1.29 |
|  | H737R | -0.916 | Destabilizing | -0.39 | Destabilizing | -0.761 | Destabilizing | 0.809 | 0.922 | -0.81 |
|  | M742I | -0.346 | Destabilizing | 0.77 | Stabilizing | 0.39 | Stabilizing | 2.049 | 0.838 | -0.94 |
|  | M742T | -0.284 | Destabilizing | 0.06 | Stabilizing | 0.35 | Stabilizing | 0.176 | 0.905 | -1.93 |
|  | M742V | -0.516 | Destabilizing | 1.23 | Stabilizing | 0.312 | Stabilizing | -0.266 | 0.824 | -1.18 |
|  | R744P | -0.757 | Destabilizing | -2.76 | Destabilizing | -1.463 | Destabilizing | 0.233 | 0.881 | -2.51 |
|  | R744H | -1.833 | Destabilizing | -1.01 | Destabilizing | -2.041 | Destabilizing | 0.763 | 0.888 | -1.6 |
|  | R744C | -1.485 | Destabilizing | -1.56 | Destabilizing | -1.799 | Destabilizing | 0.277 | 0.904 | -1.46 |
|  | R744G | -1.846 | Destabilizing | -1.64 | Destabilizing | -2.183 | Destabilizing | 0.503 | 0.878 | -2.43 |
|  | N745H | -0.851 | Destabilizing | 0.8 | Stabilizing | -0.529 | Destabilizing | -0.165 | 0.921 | -0.62 |
|  | C747S | -2.268 | Destabilizing | -1.01 | Destabilizing | -2.16 | Destabilizing | 0.301 | 0.883 | -2.47 |
|  | V748I | -0.458 | Destabilizing | 0.18 | Stabilizing | -0.101 | Destabilizing | -0.587 | 0.848 | -1.02 |
|  | P749S | -0.676 | Destabilizing | -0.27 | Destabilizing | -0.5 | Destabilizing | 2.654 | 0.813 | -1.29 |
|  | P749A | -0.746 | Destabilizing | 0.22 | Stabilizing | -0.523 | Destabilizing | 0.495 | 0.869 | -1 |
|  | P749T | -0.677 | Destabilizing | -0.14 | Destabilizing | -0.475 | Destabilizing | 0.532 | 0.86 | -1.23 |
|  | P750Q | -0.451 | Destabilizing | -0.81 | Destabilizing | -0.384 | Destabilizing | 0.548 | 0.865 | -0.45 |
|  | A752E | -0.76 | Destabilizing | -0.11 | Destabilizing | -0.529 | Destabilizing | 2.761 | 0.836 | -0.94 |
|  | A752T | -0.621 | Destabilizing | -0.16 | Destabilizing | -0.421 | Destabilizing | 1.702 | 0.837 | -1.09 |
|  | P754A | -0.351 | Destabilizing | -0.2 | Destabilizing | -0.076 | Destabilizing | 0.535 | 0.838 | -0.3 |
|  | E755G | -0.444 | Destabilizing | 0.82 | Stabilizing | -0.04 | Destabilizing | 1.852 | 0.802 | -1.06 |
|  | V756M | -0.642 | Destabilizing | -0.64 | Destabilizing | -0.649 | Destabilizing | 1.857 | 0.811 | -0.91 |
|  | P757S | -1.253 | Destabilizing | -1.44 | Destabilizing | -1.216 | Destabilizing | 0.752 | 0.866 | -1.48 |
|  | P757T | -1.199 | Destabilizing | -0.54 | Destabilizing | -0.979 | Destabilizing | 0.95 | 0.909 | -1.5 |
|  | A760T | -0.983 | Destabilizing | -0.02 | Destabilizing | -0.527 | Destabilizing | 1.311 | 0.841 | -1.44 |
|  | L761F | -0.899 | Destabilizing | -0.3 | Destabilizing | -0.779 | Destabilizing | -0.14 | 0.889 | -1.49 |
|  | S762T | -0.24 | Destabilizing | 0.45 | Stabilizing | 0.297 | Stabilizing | 0.391 | 0.902 | -1.16 |
|  | Y764C | -2.06 | Destabilizing | -1.09 | Destabilizing | -2.134 | Destabilizing | -0.359 | 0.878 | -1.77 |
|  | Y764H | -2.331 | Destabilizing | -0.4 | Destabilizing | -2.166 | Destabilizing | 0.583 | 0.878 | -2.12 |
|  | L766F | -1.578 | Destabilizing | -0.1 | Destabilizing | -1.354 | Destabilizing | 0.193 | 0.891 | -1.21 |
|  | G767R | -1.243 | Destabilizing | -2.38 | Destabilizing | -1.188 | Destabilizing | 0.963 | 0.898 | -1.8 |
|  | S768A | -0.691 | Destabilizing | 1.26 | Stabilizing | -0.075 | Destabilizing | 1.171 | 0.886 | -0.71 |
|  | S768R | -0.137 | Destabilizing | 1.09 | Stabilizing | 0.435 | Stabilizing | 0.683 | 0.938 | -1.06 |
|  | W769C | -1.837 | Destabilizing | -1.37 | Destabilizing | -1.735 | Destabilizing | 0.834 | 0.889 | -2.74 |
|  | G771E | -1.89 | Destabilizing | -0.81 | Destabilizing | -1.751 | Destabilizing | 2.209 | 0.792 | -1.61 |
|  | G772D | -2.41 | Destabilizing | -2.1 | Destabilizing | -2.591 | Destabilizing | 1.207 | 0.922 | -2.2 |
|  | K776E | -1.496 | Destabilizing | -0.1 | Destabilizing | -1.224 | Destabilizing | 3.524 | 0.771 | -1.19 |
|  | E777Q | -0.98 | Destabilizing | -1.12 | Destabilizing | -0.886 | Destabilizing | 0.275 | 0.942 | -0.83 |
|  | G778D | -0.895 | Destabilizing | -3 | Destabilizing | -1.305 | Destabilizing | 0.794 | 0.911 | -1.51 |
|  | W781L | -2.468 | Destabilizing | -3.13 | Destabilizing | -2.588 | Destabilizing | 0.267 | 0.956 | -2.02 |
|  | G782A | -0.777 | Destabilizing | 0.44 | Stabilizing | -0.394 | Destabilizing | 1.613 | 0.92 | -0.67 |
|  | G782E | -1.398 | Destabilizing | -0.48 | Destabilizing | -1.175 | Destabilizing | 0.273 | 0.885 | -1.12 |
|  | P784S | 0.127 | Stabilizing | -0.4 | Destabilizing | 0.287 | Stabilizing | 0.43 | 0.9 | -1.05 |
|  | E785K | 0.052 | Stabilizing | -0.25 | Destabilizing | 0.337 | Stabilizing | 0.391 | 0.911 | -0.73 |
|  | P786R | 0.127 | Stabilizing | -0.4 | Destabilizing | 0.287 | Stabilizing | 0.762 | 0.872 | -0.4 |
|  | P786T | -0.429 | Destabilizing | -0.38 | Destabilizing | -0.147 | Destabilizing | 0.74 | 0.889 | -0.16 |
|  | G788E | -0.888 | Destabilizing | -0.44 | Destabilizing | -0.609 | Destabilizing | 0.462 | 0.913 | -0.64 |
|  | D790N | 0.327 | Stabilizing | -0.07 | Destabilizing | 0.543 | Stabilizing | -0.379 | 0.865 | -0.35 |
|  | D791Y | 0.006 | Stabilizing | 0.26 | Stabilizing | 0.142 | Stabilizing | -0.295 | 0.857 | -0.61 |
|  | D791N | 0.632 | Stabilizing | -0.14 | Destabilizing | 0.819 | Stabilizing | -0.341 | 0.912 | -1.02 |
|  | N792S | 0.104 | Stabilizing | -1.05 | Destabilizing | 0.189 | Stabilizing | 0.459 | 0.925 | -0.36 |
|  | D793N | 0.438 | Stabilizing | 0.25 | Stabilizing | 0.69 | Stabilizing | 0.17 | 0.936 | -0.37 |
|  | Q794R | -0.241 | Destabilizing | 0.72 | Stabilizing | 0.131 | Stabilizing | 0.432 | 0.916 | -0.97 |
|  | L798F | -1.237 | Destabilizing | -0.66 | Destabilizing | -1.22 | Destabilizing | 0.102 | 0.883 | -2.28 |
|  | F800C | -0.501 | Destabilizing | -1.33 | Destabilizing | -0.569 | Destabilizing | 0.204 | 0.902 | -2.68 |
|  | S803A | -0.34 | Destabilizing | 1.72 | Stabilizing | 0.374 | Stabilizing | 1.04 | 0.913 | -0.57 |
|  | S804C | -0.299 | Destabilizing | 1.85 | Stabilizing | 1.85 | Stabilizing | 1.013 | 0.904 | -1.04 |
|  | R806H | -1.149 | Destabilizing | 0.37 | Stabilizing | -1.053 | Destabilizing | 0.805 | 0.922 | -0.48 |
|  | R806C | -0.488 | Destabilizing | -0.27 | Destabilizing | -0.55 | Destabilizing | 0.665 | 0.931 | -0.11 |
|  | R806L | 0.239 | Stabilizing | 0.31 | Stabilizing | 0.322 | Stabilizing | -0.309 | 0.864 | 0.4 |
|  | W807C | -1.003 | Destabilizing | -0.13 | Destabilizing | -0.675 | Destabilizing | 0.169 | 0.863 | -2.49 |
|  | E809Q | -0.567 | Destabilizing | -1.14 | Destabilizing | -0.471 | Destabilizing | 0.974 | 0.913 | -0.48 |
|  | H812Q | -1.431 | Destabilizing | -1.18 | Destabilizing | -1.618 | Destabilizing | 0.991 | 0.849 | -0.31 |
|  | P813L | -0.83 | Destabilizing | -0.32 | Destabilizing | -0.729 | Destabilizing | 1.153 | 0.903 | -0.97 |
|  | P813S | -1.515 | Destabilizing | 0.13 | Stabilizing | -1.315 | Destabilizing | -0.505 | 0.892 | -0.98 |
|  | G814E | -1.082 | Destabilizing | -2.58 | Destabilizing | -1.422 | Destabilizing | 0.016 | 0.824 | -1.72 |
|  | V816A | -1.988 | Destabilizing | -2.6 | Destabilizing | -2.393 | Destabilizing | -0.084 | 0.94 | -2.17 |
|  | R818L | -0.857 | Destabilizing | 1.3 | Stabilizing | -0.258 | Destabilizing | 0.638 | 0.92 | -0.92 |
|  | R818Q | -1.199 | Destabilizing | -1.78 | Destabilizing | -1.446 | Destabilizing | 0.749 | 0.93 | -1.63 |
|  | I820V | -1.392 | Destabilizing | 0.29 | Stabilizing | -0.981 | Destabilizing | 1.417 | 0.874 | -1.42 |
|  | A821G | -1.301 | Destabilizing | -0.34 | Destabilizing | -1.053 | Destabilizing | 0.322 | 0.916 | -1.45 |
|  | A821S | -1.754 | Destabilizing | -1.49 | Destabilizing | -1.681 | Destabilizing | 0.586 | 0.898 | -1.53 |
|  | A821T | -1.39 | Destabilizing | -0.71 | Destabilizing | -1.115 | Destabilizing | 1.418 | 0.908 | -1.68 |
|  | P822S | -0.603 | Destabilizing | 0.81 | Stabilizing | 0.032 | Stabilizing | 1.576 | 0.871 | -0.78 |
|  | P824L | -0.173 | Destabilizing | -0.04 | Destabilizing | 0.162 | Stabilizing | 1.05 | 0.894 | -0.05 |
|  | P824R | 0.196 | Stabilizing | -0.4 | Destabilizing | 0.353 | Stabilizing | 1.433 | 0.899 | -0.16 |
|  | P824S | -0.375 | Destabilizing | -0.84 | Destabilizing | -0.171 | Destabilizing | -0.045 | 0.916 | -0.4 |
|  | A825P | -0.357 | Destabilizing | -1.47 | Destabilizing | -0.382 | Destabilizing | 1.25 | 0.881 | -1.55 |
|  | A825T | -0.764 | Destabilizing | -0.99 | Destabilizing | -0.611 | Destabilizing | 1.68 | 0.879 | -1.77 |
|  | T826I | 0.052 | Stabilizing | 1.02 | Stabilizing | 0.41 | Stabilizing | 0.859 | 0.863 | -0.44 |
|  | T826K | -0.343 | Destabilizing | 0.18 | Stabilizing | -0.08 | Destabilizing | 1.272 | 0.907 | -0.89 |
|  | T826S | -0.3 | Destabilizing | -0.5 | Destabilizing | -0.221 | Destabilizing | 0.166 | 0.932 | -0.61 |
|  | P827S | -0.206 | Destabilizing | -0.26 | Destabilizing | -0.023 | Destabilizing | 1.73 | 0.844 | -0.58 |
|  | P827A | -0.186 | Destabilizing | 0.69 | Stabilizing | 0.196 | Stabilizing | 0.017 | 0.92 | -0.63 |
|  | M828I | -0.101 | Destabilizing | 0.43 | Stabilizing | 0.355 | Stabilizing | 0.521 | 0.898 | -0.31 |
|  | M828V | -0.18 | Destabilizing | 0.36 | Stabilizing | 0.26 | Stabilizing | 0.078 | 0.898 | -0.4 |
|  | D833E | -0.372 | Destabilizing | -0.75 | Destabilizing | -0.226 | Destabilizing | -0.499 | 0.896 | -0.51 |
|  | D833H | -0.758 | Destabilizing | 0.39 | Stabilizing | -0.525 | Destabilizing | 0.171 | 0.929 | -0.71 |
|  | G834D | -0.491 | Destabilizing | -1.83 | Destabilizing | -0.496 | Destabilizing | 1.979 | 0.858 | -1.42 |
|  | G834C | -1.004 | Destabilizing | -1.16 | Destabilizing | -1.051 | Destabilizing | 0.488 | 0.872 | -1.29 |
|  | G834S | -0.524 | Destabilizing | -2.58 | Destabilizing | -0.667 | Destabilizing | 0.678 | 0.919 | -1.37 |
|  | C837S | -0.133 | Destabilizing | -0.62 | Destabilizing | 0.188 | Stabilizing | 1.092 | 0.887 | -0.68 |
|  | S839C | -0.388 | Destabilizing | 1.34 | Stabilizing | 0.111 | Stabilizing | 0.886 | 0.848 | -0.77 |
|  | R840P | -0.8 | Destabilizing | -1.61 | Destabilizing | -1.151 | Destabilizing | 0.911 | 0.895 | -1.19 |
|  | R840Q | -1.28 | Destabilizing | -0.65 | Destabilizing | -1.226 | Destabilizing | 0.328 | 0.914 | -0.8 |
|  | R840W | -0.539 | Destabilizing | 0.37 | Stabilizing | -0.457 | Destabilizing | -0.033 | 0.881 | -0.66 |
|  | R841H | -1.734 | Destabilizing | 0.37 | Stabilizing | -1.503 | Destabilizing | 0.011 | 0.832 | -1.08 |
|  | R841C | -1.132 | Destabilizing | -0.19 | Destabilizing | -1.048 | Destabilizing | 1.303 | 0.864 | -0.88 |
|  | P842S | -2.858 | Destabilizing | -1.48 | Destabilizing | -2.958 | Destabilizing | 0.711 | 0.866 | -1.49 |
|  | L845V | -1.749 | Destabilizing | -3.1 | Destabilizing | -2.188 | Destabilizing | 0.043 | 0.925 | -1.84 |
|  | S850A | -0.313 | Destabilizing | 1.62 | Stabilizing | 0.284 | Stabilizing | 1.223 | 0.867 | -0.97 |
|  | L852F | -1.563 | Destabilizing | -0.66 | Destabilizing | -1.531 | Destabilizing | 0.034 | 0.895 | -1.82 |
|  | T853A | -1.223 | Destabilizing | 0.47 | Stabilizing | -0.867 | Destabilizing | 0.42 | 0.873 | -1.28 |
|  | Q855R | -0.162 | Destabilizing | 0.31 | Stabilizing | 0.054 | Stabilizing | 0.3 | 0.856 | -1.08 |
|  | N857K | -0.203 | Destabilizing | -0.13 | Destabilizing | 0.034 | Stabilizing | 1.216 | 0.92 | -1.18 |
|  | T859I | 0.158 | Stabilizing | 0.8 | Stabilizing | 0.672 | Stabilizing | 0.546 | 0.908 | -0.55 |
|  | T859S | -1.241 | Destabilizing | -1.24 | Destabilizing | -1.228 | Destabilizing | -0.106 | 0.865 | -0.86 |
|  | T859A | -0.828 | Destabilizing | -0.45 | Destabilizing | -0.716 | Destabilizing | 0.811 | 0.886 | -0.99 |
|  | L860P | -0.991 | Destabilizing | -4.31 | Destabilizing | -1.654 | Destabilizing | 1.481 | 0.887 | -2.35 |
|  | S864C | -0.308 | Destabilizing | 0.53 | Stabilizing | -0.067 | Destabilizing | 0 | 0.911 | -0.67 |
|  | S864T | -0.358 | Destabilizing | 0.82 | Stabilizing | 0.075 | Stabilizing | -0.451 | 0.861 | -0.84 |
|  | S864R | -0.288 | Destabilizing | 0 | Destabilizing | -0.068 | Destabilizing | 1.936 | 0.86 | -0.7 |
|  | I868M | -1.146 | Destabilizing | -0.43 | Destabilizing | -1.073 | Destabilizing | 2.878 | 0.813 | -0.97 |
|  | I868V | -1.67 | Destabilizing | -0.35 | Destabilizing | -1.467 | Destabilizing | 1.371 | 0.893 | -1.44 |
|  | V871M | -0.651 | Destabilizing | -0.87 | Destabilizing | -0.649 | Destabilizing | 1.876 | 0.849 | -1.11 |
|  | L872I | -0.581 | Destabilizing | -0.19 | Destabilizing | -0.238 | Destabilizing | 1.891 | 0.875 | -0.56 |
|  | D873G | -0.459 | Destabilizing | -0.21 | Destabilizing | -0.221 | Destabilizing | 2.595 | 0.845 | -0.32 |

**Supplementary Table S3:** Predictions of disease phenotype using PMut.

| **S. No.** | **Mutation** | | **Prediction** |
| --- | --- | --- | --- |
|  | H726R | H → R (His → Arg) | 0.21 (92%)    Neutral |
|  | G728E | G → E (Gly → Glu) | 0.06 (97%)    Neutral |
|  | G728R | G → R (Gly → Arg) | 0.19 (93%)    Neutral |
|  | Q729H | Q → H (Gln → His) | 0.23 (91%)    Neutral |
|  | S730R | S → R (Ser → Arg) | 0.65 (84%)    Disease |
|  | S730G | S → G (Ser → Gly) | 0.54 (80%)    Disease |
|  | R731L | R → L (Arg → Leu) | 0.41 (86%)    Neutral |
|  | R731Q | R → Q (Arg → Gln) | 0.12 (95%)    Neutral |
|  | R731W | R → W (Arg → Trp) | 0.56 (81%)    Disease |
|  | H737R | H → R (His → Arg) | 0.26 (90%)    Neutral |
|  | M742T | M → T (Met → Thr) | 0.44 (85%)    Neutral |
|  | R744P | R → P (Arg → Pro) | 0.50 (82%)    Neutral |
|  | R744H | R → H (Arg → His) | 0.41 (86%)    Neutral |
|  | R744C | R → C (Arg → Cys) | 0.56 (81%)    Disease |
|  | R744G | R → G (Arg → Gly) | 0.51 (79%)    Disease |
|  | N745H | N → H (Asn → His) | 0.40 (86%)    Neutral |
|  | C747S | C → S (Cys → Ser) | 0.07 (96%)    Neutral |
|  | P749S | P → S (Pro → Ser) | 0.05 (97%)    Neutral |
|  | P749T | P → T (Pro → Thr) | 0.07 (96%)    Neutral |
|  | A752E | A → E (Ala → Glu) | 0.12 (95%)    Neutral |
|  | A752T | A → T (Ala → Thr) | 0.08 (96%)    Neutral |
|  | P754A | P → A (Pro → Ala) | 0.05 (97%)    Neutral |
|  | V756M | V → M (Val → Met) | 0.06 (97%)    Neutral |
|  | P757S | P → S (Pro → Ser) | 0.19 (93%)    Neutral |
|  | A760T | A → T (Ala → Thr) | 0.00 (99%)    Neutral |
|  | Y764H | Y → H (Tyr → His) | 0.01 (99%)    Neutral |
|  | L766F | L → F (Leu → Phe) | 0.08 (96%)    Neutral |
|  | G767R | G → R (Gly → Arg) | 0.52 (79%)    Disease |
|  | W769C | W → C (Trp → Cys) | 0.43 (85%)    Neutral |
|  | G771E | G → E (Gly → Glu) | 0.26 (90%)    Neutral |
|  | G772D | G → D (Gly → Asp) | 0.24 (91%)    Neutral |
|  | K776E | K → E (Lys → Glu) | 0.19 (93%)    Neutral |
|  | G778D | G → D (Gly → Asp) | 0.14 (94%)    Neutral |
|  | W781L | W → L (Trp → Leu) | 0.37 (87%)    Neutral |
|  | G782E | G → E (Gly → Glu) | 0.18 (93%)    Neutral |
|  | L798F | L → F (Leu → Phe) | 0.14 (94%)    Neutral |
|  | F800C | F → C (Phe → Cys) | 0.77 (88%)    Disease |
|  | S804C | S → C (Ser → Cys) | 0.11 (95%)    Neutral |
|  | R806C | R → C (Arg → Cys) | 0.65 (84%)    Disease |
|  | R806L | R → L (Arg → Leu) | 0.61 (83%)    Disease |
|  | W807C | W → C (Trp → Cys) | 0.83 (90%)    Disease |
|  | E809Q | E → Q (Glu → Gln) | 0.26 (90%)    Neutral |
|  | H812Q | H → Q (His → Gln) | 0.21 (92%)    Neutral |
|  | P813L | P → L (Pro → Leu) | 0.12 (95%)    Neutral |
|  | P813S | P → S (Pro → Ser) | 0.34 (88%)    Neutral |
|  | G814E | G → E (Gly → Glu) | 0.24 (91%)    Neutral |
|  | V816A | V → A (Val → Ala) | 0.26 (91%)    Neutral |
|  | R818L | R → L (Arg → Leu) | 0.61 (83%)    Disease |
|  | R818Q | R → Q (Arg → Gln) | 0.28 (90%)    Neutral |
|  | A821G | A → G (Ala → Gly) | 0.07 (96%)    Neutral |
|  | A821S | A → S (Ala → Ser) | 0.28 (90%)    Neutral |
|  | A821T | A → T (Ala → Thr) | 0.11 (95%)    Neutral |
|  | P824R | P → R (Pro → Arg) | 0.39 (86%)    Neutral |
|  | A825P | A → P (Ala → Pro) | 0.00 (99%)    Neutral |
|  | A825T | A → T (Ala → Thr) | 0.02 (99%)    Neutral |
|  | T826K | T → K (Thr → Lys) | 0.27 (90%)    Neutral |
|  | T826S | T → S (Thr → Ser) | 0.22 (92%)    Neutral |
|  | P827S | P → S (Pro → Ser) | 0.09 (96%)    Neutral |
|  | G834D | G → D (Gly → Asp) | 0.08 (96%)    Neutral |
|  | G834C | G → C (Gly → Cys) | 0.07 (97%)    Neutral |
|  | G834S | G → S (Gly → Ser) | 0.06 (97%)    Neutral |
|  | S839C | S → C (Ser → Cys) | 0.27 (90%)    Neutral |
|  | R840P | R → P (Arg → Pro) | 0.08 (96%)    Neutral |
|  | R840Q | R → Q (Arg → Gln) | 0.01 (99%)    Neutral |
|  | P842S | P → S (Pro → Ser) | 0.23 (91%)    Neutral |
|  | L845V | L → V (Leu → Val) | 0.31 (89%)    Neutral |
|  | L852F | L → F (Leu → Phe) | 0.15 (94%)    Neutral |
|  | T859A | T → A (Thr → Ala) | 0.25 (91%)    Neutral |
|  | L860P | L → P (Leu → Pro) | 0.56 (81%)    Disease |
|  | S864R | S → R (Ser → Arg) | 0.07 (96%)    Neutral |
|  | I868M | I → M (Ile → Met) | 0.08 (96%)    Neutral |
|  | I868V | I → V (Ile → Val) | 0.06 (97%)    Neutral |
|  | V871M | V → M (Val → Met) | 0.08 (96%)    Neutral |
|  | L872I | L → I (Leu → Ile) | 0.09 (96%)    Neutral |
|  | D873G | D → G (Asp → Gly) | 0.04 (98%)    Neutral |

**Supplementary Table S4:** Predictions of disease phenotype using MutPred2.

| **S. No.** | **Substitution** | **Score** | **Description** | |
| --- | --- | --- | --- | --- |
|  | H726R | 0.052 | - | - |
|  | G728E | 0.058 | - | - |
|  | G728R | 0.102 | - | - |
|  | Q729H | 0.238 | - | - |
|  | S730R | 0.677 | Loss of Strand (Pr = 0.28 \| P = 0.01); Gain of Helix (Pr = 0.27 \| P = 0.04); Gain of Pyrrolidone carboxylic acid at Q729 (Pr = 0.04 \| P = 0.05) | LME000012\|ELME000102\|ELME000106\|ELME000108\|ELME000146\|ELME000149\|ELME000197\|ELME000 |
|  | S730G | 0.529 | Loss of Strand (Pr = 0.26 \| P = 0.05) | ELME000197\|ELME000336 |
|  | R731L | 0.596 | Loss of Intrinsic disorder (Pr = 0.35 \| P = 0.05); Loss of Strand (Pr = 0.27 \| P = 0.01) | ELME000045\|ELME000146\|ELME000197\|ELME000335\|ELME000336 |
|  | R731Q | 0.387 | - | - |
|  | R731W | 0.526 | Loss of Intrinsic disorder (Pr = 0.53 \| P = 4.4e-03); Gain of Strand (Pr = 0.27 \| P = 0.02); Gain of Pyrrolidone carboxylic acid at Q729 (Pr = 0.05 \| P = 0.04) | ELME000146\|ELME000197\|ELME000336 |
|  | H737R | 0.301 | - | - |
|  | M742T | 0.627 | Gain of Strand (Pr = 0.26 \| P = 0.04); Altered Stability (Pr = 0.14 \| P = 0.02) | ELME000146\|PS00005 |
|  | R744P | 0.776 | Gain of Loop (Pr = 0.27 \| P = 0.03) | ELME000100\|ELME000108 |
|  | R744H | 0.329 | - | - |
|  | R744C | 0.469 | - | - |
|  | R744G | 0.564 | Gain of Strand (Pr = 0.27 \| P = 0.01); Gain of Loop (Pr = 0.27 \| P = 0.04); Altered Stability (Pr = 0.09 \| P = 0.05) | ELME000100\|ELME000108 |
|  | N745H | 0.448 | - | - |
|  | C747S | 0.116 | - | - |
|  | P749S | 0.058 | - | - |
|  | P749T | 0.127 | - | - |
|  | A752E | 0.121 | - | - |
|  | A752T | 0.049 | - | - |
|  | P754A | 0.088 | - | - |
|  | V756M | 0.068 | - | - |
|  | P757S | 0.12 | - | - |
|  | A760T | 0.025 | - | - |
|  | Y764H | 0.056 | - | - |
|  | L766F | 0.162 | - | - |
|  | G767R | 0.718 | Gain of ADP-ribosylation at G767 (Pr = 0.19 \| P = 0.05) | ELME000182\|PS00008 |
|  | W769C | 0.887 | Altered Ordered interface (Pr = 0.23 \| P = 0.05); Loss of Pyrrolidone carboxylic acid at Q774 (Pr = 0.08 \| P = 0.02) | PS00008 |
|  | G771E | 0.465 | - | - |
|  | G772D | 0.635 | Gain of Pyrrolidone carboxylic acid at Q774 (Pr = 0.09 \| P = 0.01) | ELME000202\|ELME000336\|PS00008 |
|  | K776E | 0.392 | - | - |
|  | G778D | 0.415 | - | - |
|  | W781L | 0.546 | Gain of Intrinsic disorder (Pr = 0.31 \| P = 0.04) | ELME000233\|PS00008 |
|  | G782E | 0.34 | - | - |
|  | L798F | 0.441 | - | - |
|  | F800C | 0.836 | Altered Ordered interface (Pr = 0.23 \| P = 0.05); Altered Stability (Pr = 0.18 \| P = 0.02) | ELME000336 |
|  | S804C | 0.543 | Altered Ordered interface (Pr = 0.26 \| P = 0.02) | ELME000336\|PS00005 |
|  | R806C | 0.573 | Altered Ordered interface (Pr = 0.27 \| P = 7.5e-03) | PS00005\|PS00008 |
|  | R806L | 0.596 | Altered Ordered interface (Pr = 0.27 \| P = 7.3e-03); Loss of Strand (Pr = 0.26 \| P = 0.03) | PS00005 |
|  | W807C | 0.917 | Altered Ordered interface (Pr = 0.37 \| P = 1.7e-03); Loss of Strand (Pr = 0.27 \| P = 0.02) | None |
|  | E809Q | 0.165 | - | - |
|  | H812Q | 0.45 | - | - |
|  | P813L | 0.71 | Altered Ordered interface (Pr = 0.26 \| P = 0.02) | None |
|  | P813S | 0.651 | Altered Ordered interface (Pr = 0.25 \| P = 0.03) | ELME000085\|ELME000336 |
|  | G814E | 0.619 | Loss of Strand (Pr = 0.28 \| P = 8.5e-03); Altered Ordered interface (Pr = 0.25 \| P = 0.03); Altered Metal binding (Pr = 0.16 \| P = 0.05) | None |
|  | V816A | 0.44 | - | - |
|  | R818L | 0.772 | Loss of Strand (Pr = 0.27 \| P = 0.02); Altered Ordered interface (Pr = 0.25 \| P = 0.02) | ELME000097\|ELME000120\|ELME000149\|ELME000182 |
|  | R818Q | 0.602 | Altered Ordered interface (Pr = 0.26 \| P = 0.01); Loss of Strand (Pr = 0.26 \| P = 0.04) | ELME000097\|ELME000120\|ELME000149 |
|  | A821G | 0.166 | - | - |
|  | A821S | 0.115 | - | - |
|  | A821T | 0.155 | - | - |
|  | P824R | 0.383 | - | - |
|  | A825P | 0.061 | - | - |
|  | A825T | 0.061 | - | - |
|  | T826K | 0.206 | - | - |
|  | T826S | 0.087 | - | - |
|  | P827S | 0.131 | - | - |
|  | G834D | 0.09 | - | - |
|  | G834C | 0.165 | - | - |
|  | G834S | 0.059 | - | - |
|  | S839C | 0.204 | - | - |
|  | R840P | 0.573 | Loss of ADP-ribosylation at R840 (Pr = 0.28 \| P = 5.3e-03); Gain of Loop (Pr = 0.27 \| P = 0.02) | ELME000012\|ELME000063\|ELME000102\|ELME000108\|ELME000136\|ELME000159\|PS00005 |
|  | R840Q | 0.023 | - | - |
|  | P842S | 0.22 | - | - |
|  | L845V | 0.137 | - | - |
|  | L852F | 0.26 | - | - |
|  | T859A | 0.139 | - | - |
|  | L860P | 0.601 | Altered Stability (Pr = 0.38 \| P = 3.3e-03); Gain of Intrinsic disorder (Pr = 0.34 \| P = 0.02); Altered Coiled coil (Pr = 0.08 \| P = 0.05); Loss of N-linked glycosylation at N857 (Pr = 0.07 \| P = 0.02) | ELME000052\|ELME000070\|ELME000136\|ELME000159\|PS00001 |
|  | S864R | 0.058 | - | - |
|  | I868M | 0.052 | - | - |
|  | I868V | 0.037 | - | - |
|  | V871M | 0.043 | - | - |
|  | L872I | 0.046 | - | - |
|  | D873G | 0.038 | - | - |
